# Gut–X axis

**DOI:** 10.1002/imt2.270

**Published:** 2025-02-26

**Authors:** Xu Lin, Zuxiang Yu, Yang Liu, Changzhou Li, Hui Hu, Jia‐Chun Hu, Mian Liu, Qin Yang, Peng Gu, Jiaxin Li, Kutty Selva Nandakumar, Gaofei Hu, Qi Zhang, Xinyu Chen, Huihui Ma, Wenye Huang, Gaofeng Wang, Yan Wang, Liping Huang, Wenjuan Wu, Ning‐Ning Liu, Chenhong Zhang, Xingyin Liu, Leming Zheng, Peng Chen

**Affiliations:** ^1^ Department of Endocrinology and Metabolism Shunde Hospital of Southern Medical University (The First People's Hospital of Shunde) Foshan City 528308 China; ^2^ State Key Laboratory of Vascular Homeostasis and Remodeling, NHC Key Laboratory of Cardiovascular Molecular Biology and Regulatory Peptides, Beijing Key Laboratory of Cardiovascular Receptors Research, Health Science Center, The Institute of Cardiovascular Sciences and Institute of Systems Biomedicine Peking University Beijing 100191 China; ^3^ State Key Laboratory of Reproductive Medicine and Offsprings Health, Center for Global Health Nanjing Medical University Nanjing 211166 China; ^4^ Department of Plastic and Aesthetic Surgery, Nanfang Hospital Southern Medical University Guangzhou 510515 China; ^5^ Department of Laboratory Medicine, Shanghai East Hospital Tongji University School of Medicine Shanghai 200123 China; ^6^ State Key Laboratory of Bioactive Substance and Function of Natural Medicines Institute of Materia Medica, Chinese Academy of Medical Sciences/Peking Union Medical College Beijing 100050 China; ^7^ Department of Obstetrics and Gynecology, Nanfang Hospital Southern Medical University Guangzhou 510515 China; ^8^ Department of Pathophysiology, Guangdong Provincial Key Laboratory of Proteomics, School of Basic Medical Sciences Southern Medical University Guangzhou 510515 China; ^9^ Department of Medical Biochemistry and Biophysics Karolinska Institute Stockholm 17177 Sweden; ^10^ State Key Laboratory of Systems Medicine for Cancer, Center for Single‐Cell Omics, School of Public Health Shanghai Jiao Tong University School of Medicine Shanghai 200025 China; ^11^ State Key Laboratory of Microbial Metabolism, School of Life Sciences and Biotechnology Shanghai Jiao Tong University Shanghai 200240 China; ^12^ School of Medicine Southern University of Science and Technology Shenzhen China

**Keywords:** bone, brain, gut, heart, kidney, liver, lung

## Abstract

Recent advances in understanding the modulatory functions of gut and gut microbiota on human diseases facilitated our focused attention on the contribution of the gut to the pathophysiological alterations of many extraintestinal organs, including the liver, heart, brain, lungs, kidneys, bone, skin, reproductive, and endocrine systems. In this review, we applied the “gut–X axis” concept to describe the linkages between the gut and other organs and discussed the latest findings related to the “gut–X axis,” including the underlying modulatory mechanisms and potential clinical intervention strategies.

## Highlights

- The gut and gut microbiota modulate the homeostasis of extraintestinal organs.
- The gut microbiota‐derived bioactive substances regulate the development of diseases in the extraintestinal organs.
- Targeting intestinal microbiota is a promising strategy for treating the diseases of extraintestinal organs.

## Graphical abstract

The concept of “gut–X axis”: the intestine and intestinal microbiota are proven to be able to modulate the pathophysiologic progressions of the extraintestinal organs' diseases. The bioactive chemicals and/or intestinal immune cells can translocate into the circulatory system and other organs and influence the immune reactions, metabolic status, cells physiology, and so forth of extraintestinal organs, finally regulating these organs' homeostasis. Meanwhile, other organs may reversely impact the intestine, namely such regulatory axis is bidirectional.

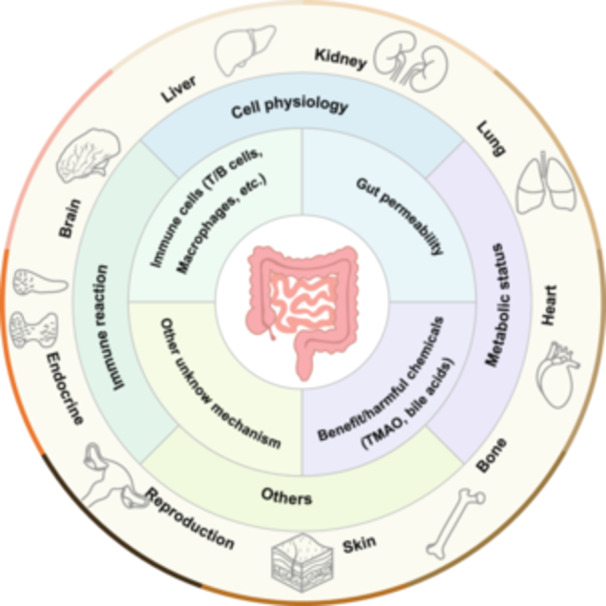

## INTRODUCTION

The gut serves as a key organ in driving the incidence and development of various extraintestinal organ diseases. For example, intestinal bacteria‐derived toxic substance trimethylamine N‐oxide (TMAO) is a well‐established inducer for metabolic and cardiovascular abnormalities. Besides, microbial‐derived beneficial subtracts such as short‐chain fatty acids (SCFAs)—acetate, propionate, butyrate, and indole derivatives—protect against multiple organ injuries. On the other hand, pathological changes in the organ “X” can influence the gut microbiota composition and functions. Thus, the interplay between the gut and extraintestinal organs is complex. Herein, we systemically revisited the current knowledge about the gut–X axis and discussed potential clinical intervention strategies.

## GUT–LIVER AXIS

### Overview

**Figure 1:**
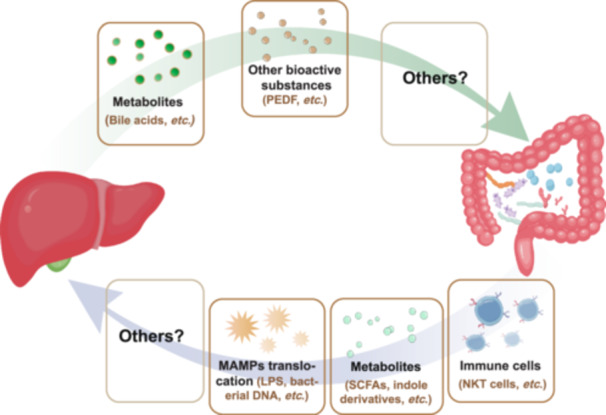
The connection between the liver and the intestine is bidirectional. Specifically, intestinal‐derived substances including MAMPs and microbial metabolites could directly influence the liver, in contrast, liver‐generated factors including primary bile acids and proteins could affect intestinal homeostasis.

Since the intestine and liver have close physiological and pathological links, the concept of the “gut–liver axis” is now widely accepted. The connection between the gut and liver has several important components. Bile acids undergo an enterohepatic circulation, and the components of the bile, including the primary bile acids and bilirubin, can be metabolized by the gut microbiota. The products of these reactions can modulate the functions of both the intestine and the liver. Furthermore, gut microbiota‐derived products, including bioactive metabolites, microbial‐associated molecular patterns (MAMPs), outer membrane vesicles (OMVs), and bacterial debris, can enter the portal vein and influence liver pathophysiology during disease development. In addition, immune cells from the gut can translocate to the liver and modulate the local immune microenvironment. Finally, both the intestine and liver can influence systemic immune and metabolic homeostasis and have indirect effects on each other during disease progression (Figure 1). In this section, we discuss the latest findings regarding the gut–liver axis in the context of specific liver diseases and future perspectives.

### Fatty liver disease

Fatty liver disease, comprising alcoholic liver disease (ALD) and nonalcoholic fatty liver disease (NAFLD), is currently the predominant condition affecting the liver worldwide. It is well established that the intestine substantially affects both ALD and NAFLD progressions.

Chronic alcohol abuse leads to intestinal bacterial overgrowth, enteric dysbiosis, and gut barrier disruption, which facilitate the translocation of MAMPs to the liver via the portal vein. The overgrowth of certain bacterial species, such as *Enterococcus*, activates toll‐like receptor (TLR)‐2 in hepatic macrophages and exacerbates liver inflammation and damage during ALD [1]. Furthermore, intestinal antimicrobial peptide deficiency increases liver inflammation and promotes ALD progression [2]. Hepatic macrophages are critical for bacterial clearance, and ethanol reduces the expression of complement receptors of the immunoglobulin superfamily in macrophages, thereby decreasing their capacity to clear bacteria and facilitating liver damage [3]. Abnormal expression or distribution of IgA and polymeric immunoglobulin receptors promotes bacterial translocation and worsens ALD [4].

Dysbiosis‐associated intestinal inflammation is one of the primary causes of greater intestinal permeability [5]. Alcoholic liver disease‐associated dysbiosis can be characterized in several ways. For example, many bacterially derived toxins are produced in larger quantities. Cytolysin, an exotoxin produced by *Enterococcus faecalis*, directly participates in the liver damage induced by chronic alcohol consumption [6]. Interestingly, bacteriophages that specifically target cytolytic *E. faecalis* markedly ameliorate ALD in mice. Whereas, the exotoxin candida lysin produced by *Candida albicans* damages hepatocytes and worsens alcoholic hepatitis [7]. In addition to bacteria and fungi, viruses present in the gut may also be involved in ALD [8]. However, it is difficult to propagate viruses in culture, and therefore, the effects of such viruses must be investigated in the future once these technical limitations have been resolved.

The intestine itself participates in the progression of ALD. Intestinal immune cells, including natural killer T cell (NKT) and Th17 cells, migrate to the liver during alcohol administration, where they have harmful effects on hepatocytes [9, 10]. The IL‐22 produced by intestinal immune cells is important for the maintenance of gut homeostasis, and the progression of ALD is associated with lower IL‐22 secretion. Importantly, restoration of the IL‐22 concentration ameliorated ALD in mice [11]. The intestinal aryl hydrocarbon receptor (AhR), which recognizes many bioactive substances, including indole derivates, is believed to protect against ALD [12], and the engineered bacteria generating AhR agonists alleviated ALD [13]. Thus, both the gut and gut microbiota have important effects on ALD progression. However, the mechanisms involved should be characterized in more detail.

Nonalcoholic fatty liver disease/nonalcoholic steatohepatitis (NAFLD/NASH, currently also known as metabolic dysfunction‐associated steatotic liver disease (MASLD)/metabolic dysfunction‐associated steatohepatitis [MASH]) is a long‐term hepatic disorder characterized by a complex pathogenesis involving the gut and gut microbiota. Similar to ALD, enteric dysbiosis and gut barrier disruption have been documented in NAFLD patients as well. It is known that intestinal barrier dysfunction develops during the early stages of NASH, and the maintenance of the gut barrier ameliorates NASH‐associated bacterial translocation and liver damage [14]. The development and severity of NAFLD/NASH are associated with alterations in the composition and functions of gut microbiota. For example, the expression of genes involved in the generation of pro‐inflammatory microbial products is markedly upregulated in pediatric patients with NAFLD/NASH [15]. The metabolic functions of the gut microbiota differ in NAFLD patients [16]. Moreover, the gut microbial metagenomic signature could be used for the noninvasive diagnosis of NAFLD‐associated liver fibrosis in humans [17]. Notably, the specific bacterial taxa showing an abnormal abundance and function in NAFLD/NASH differed among the cohorts studied, possibly because of variations in ethnicity and disease severity.

Many bacterial taxa and their products affect NALFD. Odoribacteraceae induces the expansion of a specific subtype of macrophages in the liver, where it participates in NASH progression [18]. Classic probiotics such as *Akkermansia* and *Bifidobacterium* prevent NAFLD in mice [19, 20]. In addition, secondary bile acids produced by gut bacteria, such as hyodeoxycholic acid (HDCA), ameliorate NAFLD by increasing the abundance of the probiotic *Parabacteroides distasonis* and lipid catabolism [21]. A novel bile acid, 3‐succinylated cholic acid, has recently been shown to increase the abundance of *Akkermansia muciniphila* and ameliorate NAFLD [22]. Besides, the gut microbiota degrades nicotine in the intestine and mitigates the nicotine‐associated progression of NAFLD [23]. Apart from the bacteria, the intestine itself participates in NAFLD. For example, intestinal peroxisome proliferator‐activated receptor (PPAR) alpha affects the uptake of dietary fat in the gut and promotes NASH progression [24]. Since the fatty liver is closely associated with cardiovascular diseases, it is noticeable that the modulation of NAFLD through the intestine may also influence metabolic and cardiovascular abnormalities.

### Toxin‐induced acute liver injury

The liver is the most important detoxification organ, and it is exposed to many endogenous and exogenous toxins during daily life that can cause damage. Carbon tetrachloride (CCl_4_) toxin is most widely used to establish animal models of chemically induced hepatotoxicity. CCl_4_ disrupts intestinal homeostasis [25], and targeting the gut microbiota may be an effective means of mitigating CCl_4_‐induced acute liver injury [26]. This may be due to a rapid disruption of liver function by CCl_4_ affecting communication between the liver and gut. The maintenance of enteric eubiosis would likely support hepatic anti‐inflammatory and antioxidative responses and protect against acute hepatotoxicity.

Acetaminophen (APAP; paracetamol) overdose is recognized as the leading cause of acute liver failure in developed countries. Damage‐associated molecular patterns (DAMPs) have long been recognized as the principal contributors to liver inflammation and subsequent hepatocyte damage. However, recent studies identified APAP‐mediated rapid disruption of the gut barrier [27, 28]. The gut microbiota also participates in APAP‐induced acute liver failure. For example, *A. muciniphila* maintains enteric bacterial function and reduces hepatic glutathione depletion and liver inflammation, leading to protection against APAP‐induced liver damage [29]. In addition, bacteria‐derived phenylpropionic acid has been shown to determine the susceptibility of mice to APAP‐induced acute liver damage [30], possibly through an effect on cytochrome P450 2E1 (CYP2E1) expression. We recently found the role of *Bifidobacterium*‐derived indole‐3‐carboxylic acid (I3C) in directly targeting CYP2E1 and suppressing its activity, which also protects against APAP‐induced acute liver injury [31]. Thus, microbial products may have significant effects on the liver pathophysiology induced by APAP. However, liver injury induced by other drugs may also be influenced by the gut microbiota. For example, deglucuronidation of tacrine is affected by the gut microbiota, and a higher level of deglucuronidation in the gut leads to greater systemic exposure to tacrine, which increases enterohepatic recycling and worsens liver injury in rats [32].

Aflatoxin B1 (AFB1), a fungal metabolite and food contaminant, is highly hepatotoxic. AFB1 disrupts intestinal farnesoid X receptor (FXR) signaling, but beneficial interventions such as melatonin treatment increase intestinal FXR expression and reduce both the gut and liver abnormalities following AFB1 exposure [33, 34]. Moreover, probiotic administration ameliorates the toxic effects of AFB1 in humans [35], but the underlying mechanisms require further investigation. These findings imply the contribution of intestinal and gut microbiota in influencing toxin‐induced acute liver damage.

### Cholestatic liver diseases

Cholestasis can be induced by many hepatic insults. Autoimmune reactions are one of the main causes of cholestatic liver diseases (CLDs). Primary biliary cholangitis (PBC) and primary sclerosing cholangitis (PSC) are the predominant forms of CLD induced by autoimmune dysfunctions. It is known that the overall gut microbial composition of PBC patients is abnormal, and specifically, *Faecalibacterium* is much less abundant in patients with certain types of PBC. Ursodeoxycholic acid, a functional secondary bile acid, is the most effective drug for the treatment of CLD, which partially restores the dysbiosis that characterizes PBC [36]. Primary sclerosing cholangitis patients also show enteric dysbiosis, involving changes in both microbial composition and metabolites [37]. These data imply the key role of gut microbiota in the pathogenesis of PBC and PSC.

Dysbiosis in CLD patients can independently cause liver injury, as evidenced by fecal microbial transplantation experiments, and therefore, disruptions in the normal biota may drive the CLD progression [38]. Primary biliary cholangitis‐associated dysbiosis increases NOD‐like receptor pyrin domain containing 3 (NLRP3)‐mediated hepatic inflammation, but the mechanisms involved are complex. Moreover, *Lactobacillus gasseri* from the gut can induce hepatic and systemic inflammation, characterized by an increase in γδ‐TCR^+^ cells and IL‐17 production, causing severe liver damage and fibrosis [39]. In addition, in mice, *Enterococcus faecalis* is harmful to the liver, which accelerates CLD progression, while Lachnospiraceae administration reduces liver inflammation and fibrosis [40]. These observations demonstrate the complexity of interactions between the gut microbiota and CLD. Apart from PBC and PSC, intrahepatic cholestasis of pregnancy (ICP) is another commonly diagnosed CLD in clinics. Intestinal *Bacteroides fragilis* inhibits signaling through FXR, which is responsible for an increased bile acid synthesis and ICP initiation in mice [41]. Thus, the gut microbiota has a substantial influence on CLD development, possibly due to bacteria‐mediated bile acid metabolization or the effect of bile acids on bacterial replication and functions. In addition, the intestinal microbiota affects the systemic and hepatic immune responses and determines the immune microenvironment.

### Liver fibrosis/cirrhosis

Liver fibrosis is a consequence of chronic or repetitive liver damage, resulting in liver failure or cancer. Theoretically, acute insults contributing to liver damage may also influence fibrogenesis. As for other types of liver disease, liver fibrosis/cirrhosis is accompanied by gut dysbiosis. Interestingly, many oral bacteria are found more distally in the gut of cirrhosis patients [42]. The underlying mechanism may be complex. One possibility is diminished intestinal antimicrobial capacity during cirrhosis, affecting mouth bacterial clearance. However, the implications of this finding are unknown. Furthermore, the fungal diversity of cirrhosis patients is also abnormal, and the Bacteroidetes/Ascomycota ratio can be used to predict the risk of 90‐day hospitalization [43].

The gut microbiota comprises commensals that prevent liver fibrosis, as evidenced by the greater fibrosis observed in germ‐free mice [44]. This phenotype may be attributed to microbiota‐mediated training of the host immune system. However, intestinal abnormalities may subsequently augment liver fibrosis. In experimental fibrosis, intestinal FXR signaling is impaired, which exacerbates the gut–vascular barrier dysfunctions and bacterial translocation, which promotes liver damage [45]. Hepatic stellate cells (HSCs) play a pivotal role in the accumulation of extracellular matrix, and the gut microbiota may directly or indirectly modulate the activity of HSCs. For example, the microbial metabolite 10‐hydroxy‐cis‐12‐octadecenoic acid suppresses Smad3 signaling in HSC and inhibits its fibrogenic activity [46]. In contrast, 3‐indole propionic acid activates HSCs through ROS‐dependent MAPK signaling pathway [47]. Thus, the effects of the gut microbiota on liver fibrosis are complex.

Targeting the gut microbiota may be an effective approach to treat liver fibrosis. *Lactobacillus rhamnosus* GG (LGG) administration restores intestinal FXR signaling and reduces hepatic concentrations of bile acids, causing amelioration of experimental cholestatic fibrosis [48]. In addition, fecal microbiota transplantation (FMT) is a promising means of attenuating liver fibrosis. For example, feces from cirrhosis patients may augment neuroinflammation [49], whereas feces from a healthy donor improves the cognitive function of patients suffering from hepatic encephalopathy [50]. However, before the utilization of this approach in the clinics, its long‐term safety and efficacy require further careful analysis.

### Liver cancer

Liver cancer most frequently occurs as hepatocellular carcinoma (HCC) and intrahepatic cholangiocarcinoma (ICC). The gut microbiota affects the incidence, development, and therapy of both HCC and ICC. Fatty liver‐associated HCC is closely linked to gut dysbiosis, characterized by depletion of probiotics. Importantly, eubiosis restoration prevents HCC, implying dysbiosis is an inducer of HCC [51]. Dysbiosis may lead to systemic inflammation, and the plasma concentrations of IL8, IL13, and CCL3 are high in HCC patients [52]. Similarly, as ICC progresses, patients demonstrate greater abundance of certain bacterial taxa (Ruminococcaceae) in the gut, abnormal microbial metabolism, and altered cytokine expression [53]. Thus, the assessment of gut microbial composition may represent a noninvasive diagnostic method for detecting early HCC [54].

The gut microbiota is also involved in liver cancer pathogenesis. Gut microbiota‐derived secondary bile acids affect hepatic CXCL16 expression and NKT cell accumulation, which inhibits liver cancer progression [55]. In addition, the Gram‐positive bacterial component lipoteichoic acid suppresses antitumor immunity via the COX2/PGE2 axis and accelerates HCC progression [56]. In addition, *Lactobacillus reuteri*‐derived tryptophan metabolites inhibit liver tumorigenesis through the AhR/SREBP2 axis [57]. Intestinal bacteria can also affect the abundance of myeloid‐derived suppressor cells (MDSCs) and liver cancer progression; for example, Gram‐negative bacteria may cause the accumulation of MDSCs and promote liver cancer [58]. However, the Gram‐negative bacterial species *A. muciniphila* reduces the abundance of MDSCs [59]. Thus, not all Gram‐negative bacteria have the same effects on MDSCs. *Enterococcus faecalis* colonization of the gut influences TLR4 signaling and accelerates HCC progression [60]. In addition, microbially generated acetate induces a hyper‐O‐GlcNAcylation state and accelerates HCC development [61]. However, another study showed suppression of the IL‐6/JAK1/STAT3 signaling by acetate binding to G‐protein‐coupled receptor 43 (GPR43), preventing HCC progression [62]. These contradictory findings indicate the complex contribution of microbiota and microbial products to liver cancer. Therefore, further understanding of the etiologic mechanisms of liver cancer is required.

Novel treatments for liver cancer may be obtained by targeting the gut microbiota. For example, Prohep, a probiotic mixture, benefits the intestinal microbial community and slows HCC progression [63]. *Bifidobacterium longum* accelerates recovery of liver functions in postoperative patients [64], and *A. muciniphila* participates in response to anti‐PD‐1 immunotherapy in HCC patients [65]. Thus, normalization of the abundance of these bacterial species may increase the efficacy of immunotherapy. Indeed, butyrate synergistically improves the effects of sorafenib [66]. However, inappropriate manipulations of the gut microbiota, such as using an overdose of prebiotics (soluble fiber inulin), may predispose toward HCC [67]. Therefore, further candidate treatments should be investigated to optimize the outcomes of liver cancer patients.

### Section summary

In conclusion, the intestine and gut microbiota may affect the progression of almost all types of liver disease. The causal role played by the gut microbiota is now clear: They may represent upstream modulators of liver dysfunctions. However, the mechanisms involved have not been well characterized. Future studies should aim to further characterize and explore the molecular mechanisms of the gut–liver axis by identifying additional beneficial intestinal molecules/cells and microbial species/products. Such findings require rapid translation to permit improved treatments for liver diseases. We expect that more effective approaches targeting the gut and gut microbiota will be identified soon. Finally, the gut–liver axis shows bidirectional regulation; that is, the liver may also modulate intestinal homeostasis. For example, a pigment epithelium‐derived factor (PEDF) produced in the liver inhibits intestinal stem cell hyperproliferation and may participate in the progression of colitis [68]. However, the studies performed to date have mainly focused on how the gut influences the liver, and therefore, future studies should also focus on the effect of the liver on intestinal diseases. Besides, with the development of advanced technologies, we need to integrate these useful approaches (i.e., bacteria single‐cell sequencing, engineered microbiota, and phage technology) into the investigation of the gut–liver axis. Finally, the successful translation into clinic is quite limited; hence, clinic trials based on promising basic research are urgently required.

## GUT–KIDNEY AXIS

### Overview

**Figure 2:**
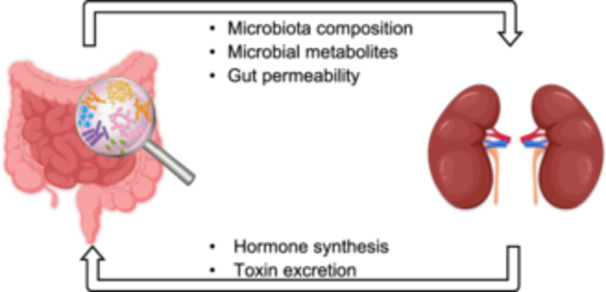
Gut–kidney axis crosstalk mechanisms. Intestinal homeostasis can affect the kidney in multiple ways, including microbial composition, gut barrier permeability, and the gut microbiota derived‐functional metabolites (bile acids, short‐chain fatty acids, neurotransmitters, uremic toxins, and inflammatory factors, etc.). Meanwhile, the kidney can excrete gut‐derived toxins and synthesize hormones to regulate intestinal homeostasis and form a two‐way crosstalk mechanism.

The interactions between the gut microbiota and the host can affect the functions of other organs as well. The gut–kidney axis refers to the bidirectional regulatory mechanism between the gut microecosystem and the kidneys through the production of metabolites by microorganisms. Gut microbiota affects the kidney by producing bile acids, SCFAs, neurotransmitters, uremic toxins (UTs), and inflammatory factors. Meanwhile, the kidneys can excrete gut‐derived toxins and synthesize hormones to regulate intestinal homeostasis [69, 70] (Figure 2). When the gut microecosystem of nephropathy patients is disturbed, nephrotoxin production will increase, causing damage to renal functions. Since then, the accumulation of toxins damages the intestinal barrier, eventually forming a vicious cycle between the gut and the kidneys.

### Gut microbiota and kidney diseases

#### Chronic kidney disease

Chronic kidney disease (CKD) is one of the major public health problems, affecting 10%−15% of the global population [71]. The main manifestation of gut microbiota in CKD patients is a decrease in microbial diversity, with a reduced level of SCFA‐producing bacteria such as Bifidobacteriaceae, *Lactobacillus*, *Blautia*, and *Roseburia* [72], which implies that a high‐fiber diet may be helpful for CKD treatment. Protein‐decomposing bacteria like Enterobacteriaceae, Enterococcaceae, and *Klebsiella* are increased. These bacteria can produce UTs such as *p*‐cresol sulfate (PCS) and indoxyl sulfate (IS), causing damage to the intestinal barrier and kidneys [73]. This suggests that high‐protein diets may impair kidney functions and increase the risk of end‐stage renal disease (ESRD) [74]. In addition, the choline‐derived gut microbiota metabolite TMAO can aggravate fibrosis in CKD patients by activating the NLRP3 inflammasome [75]. Moreover, high levels of TMAO can increase mortality in CKD patients by 2.8 times and predict a poor prognosis for treatment [76].

Since UTs cannot be removed by dialysis, the accumulation of UTs in the body will accelerate the incidence of ESRD. Different from CKD patients, serum secondary bile acid (SBA) level is significantly increased in ESRD patients, and the abundance of SBA‐synthesizing genes of *Eggerthella lenta* and *Fusobacterium nucleatum* is increased with a significant decrease in *Faecalibacterium prausnitzii* [77]. With the progression of CKD, the composition and functions of gut microbiota will also change, accelerating the transformation to ESRD. This suggests that the gut microbiota is a significant biomarker that needs to be monitored in CKD patients. It also provides important tips for the treatment strategy based on gut microbiota. Regulation of gut microbiota is expected to become a complementary therapy for dialysis.

#### Diabetic nephropathy

Diabetic nephropathy (DN) is one of the most common complications of diabetic microangiopathy and an important cause of ESRD. The imbalance of gut microbiota is one of the key triggers of DN, mainly manifesting as an increased level of *Escherichia*, *Citrobacter*, and *Klebsiella* with a decrease in *Roseburia* [78]. However, not only the gut microbiota but also the gut virome of DN patients are changed. The virus abundance and diversity are significantly reduced, especially the function of phages lysing host bacteria [79]. These results suggest that combining gut viral and bacterial markers may be a good diagnostic method for DN. Changes in the intestinal microecological composition will inevitably cause changes in metabolites. Hu et al. found that circulating acetate in DN patients upregulates GPR43 expression, increases cholesterol accumulation in the renal tubular epithelial cells, and disrupts cholesterol homeostasis, leading to proteinuria and renal tubular interstitial injuries [80]. This also hints at SCFAs' different roles in various diseases. At the same time, apart from promoting CKD pathogenesis, aromatic amino acids are also risk factors for DN. For example, tyrosine is converted into phenylsulfate (PS) under the action of a series of enzymes like tyrosine lyase produced by the gut microbiota, which promotes proteinuria [81]. Exosomes as a tool for intracellular communication will become pathogenic factors in the disturbed intestinal environment. For instance, gut microbiota‐derived outer membrane exosomes are increased in DN rats, which damaged the intestinal vascular barrier and activated the caspase‐11/1 pathway, triggering inflammation in kidneys [82]. This observation provides new insights into the relationship between gut microbiota disturbance and DN pathogenesis, making gut microbiota a promising therapeutic target for DN.

#### Immune‐associated nephropathy

Since the gut is an important immune organ of the body, the relationship between gut microbiota and autoimmune diseases has also received much attention. Lupus nephritis (LN) is the most common complication of systemic lupus erythematosus (SLE), and there is no effective treatment in clinic [83]. LN patients have dysregulated gut microbiota, with a significant change in diversity and complexity [84]. Among them, an increase in *Ruminococcus gnavus* (Rg) is most obvious. Rg specifically expresses cell membrane lipoglycan and induces the host to spontaneously produce high levels of antigen‐specific IgG antibodies, one of the pathogenic factors in SLE [85]. At the same time, a low‐fiber diet and other dietary habits that destroy intestinal homeostasis will also accelerate LN pathogenesis by increasing intestinal leakage and activating adaptive immunity [86].

IgA nephropathy (IgAN) is the most common primary glomerulonephritis and is classified as a type of autoimmune disease. Intestinal homeostasis imbalance may mediate IgAN prevalence. In IgAN patients, *Escherichia*, Veillonaceae, *Akkermansia*, and *Bacteroides* are significantly upregulated, while *Dialister* is significantly downregulated [87]. Mechanistically, deglycosylation of IgA by an upregulated *Akkermansia* exposes neoepitopes for serum IgG binding, which leads to immune complex formation and IgAN development [88]. In addition, intestinal mucosal immunity may also be involved in IgAN pathogenesis. For example, a lower level expression of gamma‐aminobutyric acid transporter‐2 (GAT‐2) in B cells changes intestinal IgA^+^ B cell response and gut microbiota composition, activates GABA‐mammalian rapamycin complex 1 target (mTORC1) axis, promotes germinal center B cell differentiation, and induces colon SIgA production [89]. In summary, these studies provide a deeper understanding of the relationship between gut microbiota and autoimmune nephropathy and offer new ideas to explore the mechanisms behind gut and immune diseases.

#### Other kidney diseases

In addition to chronic diseases, changes in the intestinal bacteria composition after acute renal injuries (kidney stones and ischemia‐reperfusion) have also been reported. Some studies also pointed out the effects of gut microbiota on the response of renal cell carcinoma patients to immune checkpoint inhibitors [90]. However, most of these studies stay at the correlation level and lack the elucidation of deep causal mechanisms. Even then, this provides a new direction for the follow‐up studies on the gut–kidney axis.

### Gut microbiota and treatment of kidney diseases

#### Medication

All the above studies have proved that gut microbiota can be used as a key target for treating kidney diseases with enormous clinical development potential. Drugs targeting gut microbiota are becoming a potential strategy for treating kidney diseases. Traditional Chinese medicines (TCM), especially those that are difficult to absorb orally, will inevitably interact with the gut microbiota [91]. The active components of TCM may protect kidneys by regulating gut microbiota [92]. Berberine (BBR) is one of the most studied original natural drugs in China in recent years, and its improvement of metabolic diseases like atherosclerosis and diabetes [93, 94, 95, 96] through gut microbiota has been confirmed by multiple studies. BBR has the efficacy of alleviating CKD by inhibiting *Clostridium_sensu_stricto_1* (*p*‐cresol producing bacteria) abundance and TyrB, a key enzyme in the tyrosine‐*p*‐cresol pathway, activity causing a reduction in the level of p‐cresyl sulfate (PCS). BBR can also increase the abundance of butyrate‐producing bacteria and butyric acid levels. These mechanisms together contribute to the renal protective activity of BBR [97, 98]. This study was rated as an important medical development (pharmacy & pharmacology field) in China during 2023, providing new evidence for studying the mechanisms of TCM through the gut–kidney axis. Similarly, flavonoids are also a hot spot in the field of gut microbiota. Isoquercitrin inhibits hydrogen proton potential by regulating gut microbial electron transport chain and tryptophan transport and reduces indole biosynthesis [99]. In addition to inhibiting gut microbiota‐derived UTs, Magnesium salvianolate B alters the intestinal bile acid metabolism and delays DN progression [100]. Macromolecular polysaccharides present in TCM are also closely related to gut microbiota. Moutan polysaccharide improves intestinal barrier functions by increasing SCFAs and reducing branched‐chain amino acids (BCAAs) in DN rats, thereby inhibiting inflammation and improving kidney injuries [101]. In addition to TCM, chemical drugs also contribute to kidney protection through the gut–kidney axis. Depletion of intestinal microbiome by oral broad‐spectrum antibiotics in mice reduced the level of TMAO, which alleviated the transition from acute kidney injury to chronic kidney disease [102]. Therefore, targeted elimination of specific pathogenic bacteria might be used to control the development of kidney diseases. Sodium‐glucose cotransporter‐2 (SGLT2) inhibitors are novel hypoglycemic agents, which can also reduce the circulating UT levels by decreasing the abundance of phenylalanine and tryptophan metabolizing gut microbiota [103].

However, in addition to therapeutic effects, some drugs can induce nephrotoxicity through gut microbiota. For example, long‐term consumption of *Rhizoma alismatis* causes structural disturbances of gut microbiota, leading to an imbalance of amino acid and phospholipid metabolism in kidneys [104]; After oral administration of matrine, an active ingredient of *Sophora flavescens*, the intestinal metabolite hippuric acid is significantly reduced, which causes an imbalance of renal energy metabolism [105]; Cisplatin, as a widely used chemotherapeutic drug, aggravates intestinal barrier damage resulting in a high level of endotoxemia forming a micro‐inflammatory environment, which facilitates its renal toxicity [106]. This also suggests that gut microbiota needs to be fully considered in the rational use of clinical drugs.

#### Probiotics

As a new intervention to regulate gut microbiota, probiotics have received more attention in the treatment of CKD [107]. *F. prausnitzii* produces butyric acid and exerts renal protective activity by acting on the G‐protein‐coupled receptor‐43 [108]. Prophylactic supplementation with *Lactobacillus casei* Zhang elevates SCFAs and niacinamide and reduces inflammatory responses in the renal macrophages and tubular epithelial cells [109]. *Bacteroides fragilis* upregulates renal glucose transporter SGLT2 expression, increases reabsorption of 5‐anhydroglucitol, and eventually activates bile acid receptor TGR5 to inhibit oxidative stress in CKD mice [110]. *Lactobacillus paracasei* HII01 promotes enterotoxins excretion by improving the organic anion transporter 3 (OAT3) transporter function in the kidney [111]. Probiotics have a good therapeutic efficacy in not only CKD but also other kidney diseases. *Lactobacillus* reduces IL‐6 in the gut while increasing IL‐10, tilts the Treg‐Th17 balance in favor of T regulatory cells (Tregs), thereby improving LN [112]. Oral administration of *Oxalobacter formigenes* prevents calcium oxalate crystal deposition in rat kidneys and reduces kidney stone formation [113]. Thus, probiotic therapy facilitates exploring the molecular mechanisms of disease development further and expands the horizon for clinical treatment. A long‐term intervention of gut microbiota as a therapy is the direction for future research.

#### Diet

Diet intervention can also delay or even improve renal disease through gut microbiota modulation. Sulfur‐containing amino acids in dietary proteins can affect protein functions through posttranslational modifications. For example, the thiolation of the tryptophan enzyme (TnaA) in the gut bacteria inhibits their ability to produce indole and reduces UT generation [114]. In addition, dietary habits like fasting, consumption of diets having 80%−100% sulfur‐containing amino acids (SR80/100), and calorie restriction diets may also exert renal protection by regulating cysteine oxidation and hydrogen sulfide‐dependent catabolism [115]. These results demonstrate the influence of microbiome intervention on the host and have reference value for the study of intestinal microbiome–host interactions and the development of dietary interventions to alleviate kidney diseases.

### Section summary

**Figure 3:**
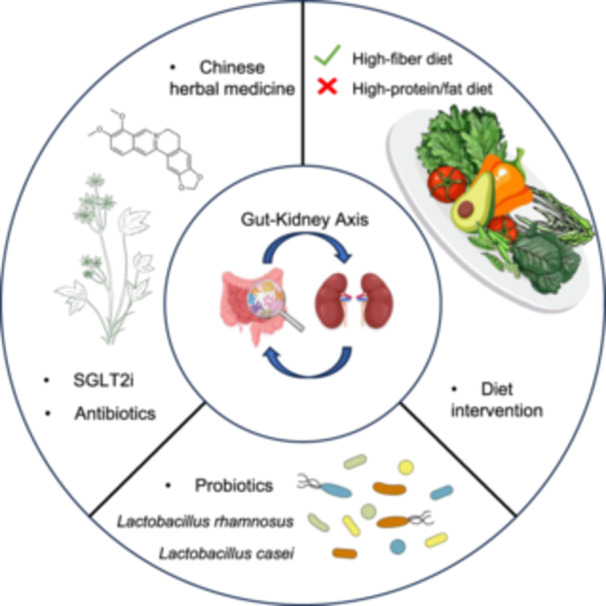
Intervention strategies based on the gut–kidney axis. There are three main ways to intervene kidney disease by gut microbiota, including diet, probiotics, and drugs. High‐protein/fat diets aggravate kidney disease by disrupting the intestinal microbiome, while high‐fiber diet can restore gut microbiota homeostasis to help protect the kidneys. Probiotics like *Lactobacillus* alleviate kidney disease by producing beneficial metabolites. Drug therapy, represented by natural drugs derived from Chinese herbal medicine and antibiotics, mainly produces renoprotective activity by inhibiting UT production and anti‐inflammatory and anti‐oxidative mechanisms.

The widespread use of multiomics technologies has greatly deepened our understanding of the role of gut microbiota in health and disease [116]. With the progression of kidney disease, the intestinal microecological imbalance is difficult to compensate for, and UT production increases, which not only accelerates the loss of kidney functions but also increases the incidence of cardiovascular events and mortality. This also indicates that the homeostasis imbalance of the gut–kidney axis can also affect the normal functioning of other axes. By restoring intestinal homeostasis, kidney diseases can be controlled. Most nephropathy therapies targeting intestinal microecology are aimed at restoring intestinal bacterial composition and functions to reduce UT production such as drugs, probiotics, and dietary therapies (Figure 3). The application of gut microbiome research in the treatment of kidney disease has a great prospect, but there are still a series of challenges to be overcome in the clinical translation process, such as individual differences in efficacy, the optimal dosage and formulation of probiotics and prebiotics, ethical requirements, and deeper mechanism exploration. These obstacles require more clinical studies to further optimize treatment strategies and also provide deeper insights into precision medicine.

## GUT–LUNG AXIS

### Overview

**Figure 4:**
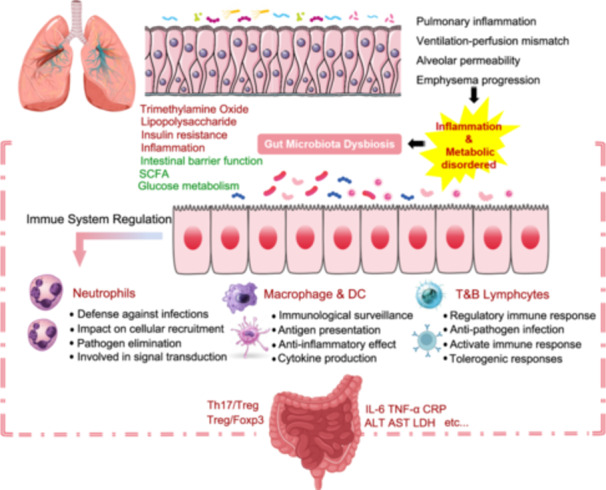
The gut–lung axis is mediated by immune and endocrine pathways. The gut–lung axis affects pulmonary inflammation, ventilation‐perfusion mismatch, alveolar permeability, and emphysema through immune cell migration and gut microenvironment changes. ILC2 and ILC3 cells can damage airway epithelial cells, increasing mucus and accelerating inflammation. Conversely, dendritic cells and monocytes can reduce lung inflammation. Gut microenvironment alterations release cytokines like IL‐17 and TNF‐*α*, which travel to the lungs to reduce infection risk. Gut microbiota stimulates B cells to produce IgA, enhancing lung immunity and slowing dysbiosis. This boosts neutrophil responses, reduces cytokine production, decreases mast cell activity, and promotes tolerance in lymphocytes.

The gut–lung axis is gaining increasing attention, as the stability of the gut microecology (homeostasis of the gut microbiota) plays a crucial role in maintaining pulmonary health. Dysbiosis of gut microbiota can lead to a variety of lung diseases through the gut–lung axis, including viral pneumonia, asthma, tuberculosis, and chronic obstructive pulmonary disease (COPD) [117]. Clinical evidence has confirmed the existence of a pathophysiological connection between the lungs and the large intestine. The structural homology between the lungs and intestines constitutes the foundation of the “gut–lung axis,” with the lungs, trachea, respiratory tract epithelium, and intestines all originating from the endoderm during embryonic development [118, 119]. The lower lobe of the lungs is adjacent to the transverse colon, and both are connected through the diaphragm [120]. Physiologically, the lungs and large intestine also share functional links. The lungs primarily facilitate the inhalation of oxygen and the exhalation of carbon dioxide, while the large intestine is mainly responsible for the absorption and excretion of water and electrolytes. Thus, their functions complement each other, collectively maintaining the body's normal physiological functions. This includes typical mucosal structures, secretory IgA, cytokines, innate lymphoid cells, B lymphocytes, and other immune cells. The gastrointestinal and respiratory mucosa are integral components of the common mucosal immune system (Figure 4). The lymphocyte homing to the mucosa is selective and a prerequisite for mucosal immunity. When a mucosal site is affected, the local immune responses are activated via the mucosal immune pathway, leading to a generalized immune response with varying degrees of reactivity at different mucosal sites. For instance, intestinal microbes can induce the production of innate lymphoid cells (ILCs) type 2 and 3, which migrate to the respiratory tract through the lymphatic and circulatory systems, enhancing immune activities in the respiratory system [121]. This interplay may also exist in diseases, as segmented filamentous bacteria (SFB)‐specific Th17 cells with dual T cell antigen receptors, one specific for SFB and another for self‐antigens, can migrate to the respiratory tract and cause pulmonary damage [122]. Conversely, microbial infections in the respiratory tract can reduce SFB numbers and increase *Escherichia coli* population in the gut, thereby stimulating an increased expression of IL‐15 in intestinal epithelial cells, promoting Th17 cell polarization, and potentially leading to intestinal immune damage [123]. Moreover, the endocrine pathways of the gut–lung axis are also crucial. Intestinal and pulmonary communication is primarily mediated through the circulatory transport of soluble microbial components and metabolites. Intestinal microbiota components, like PAMPs, interact with the innate immune system's pattern recognition receptors, such as Toll‐like receptors (TLRs), to trigger inflammatory responses, thereby activating the immune system [124]. Activation of intestinal TLR signaling triggers the pulmonary immune system [125, 126, 127]. Furthermore, innate immune cells from the intestinal mucosa can directly migrate to the respiratory tract through the peripheral circulation and participate in the maintenance of respiratory immune homeostasis. ILCs residing at the mucosal surfaces can enhance immune responses, maintain mucosal integrity, and preserve tissue homeostasis. IL‐25 or helminth‐induced inflammatory ILC2s can migrate into the lymphatic and blood circulation in a sphingosine 1‐phosphate (S1P)‐dependent manner, eventually accumulating in the lungs to combat helminth infections and promoting tissue repair [128].

The gut microbiota not only regulates the immune responses in the gastrointestinal tract but also affects the health and disease of distant organs like the respiratory system. Through regulation of the gut microbiome, the lungs and intestines are physiologically interconnected and can pathologically influence each other. This section discusses the relationship between gut microbiota and respiratory diseases, mechanisms of action of the gut–lung axis, and clinical applications.

### The role of gut–lung axis in the pathogenesis of respiratory disorders

#### Chronic obstructive pulmonary disease (COPD)

COPD progression in patients is closely associated with gut microbiomes [129]. COPD patients exhibit significant differences in the diversity and composition of gut microbiota compared to healthy controls (Table 1), predominantly characterized by phyla Firmicutes, Bacteroidetes, Proteobacteria, Actinobacteria, and Verrucomicrobia at the phylum level [130]. The pulmonary microbiome, consisting of genera such as *Prevotella*, *Veillonella*, *Pseudomonas*, *Acinetobacter*, *Fusobacterium*, *Sphingomonas*, *Rothia*, *Staphylococcus*, and *Streptococcus*, constitutes the “core microbiome” distinguishing COPD patients from the normal population [131]. Additionally, genera like *Achromobacter*, *Gemella*, and *Capnocytophaga* have been identified in the lower respiratory tract of COPD patients [132]. Further, a correlation between a decline in lung function in COPD patients and multiple genera, including *Streptococcus*, *Streptomyces*, and *Veillonella*, has been identified [133].

**Table 1:** Dysbiosis in the lungs and gut influences COPD, asthma, and lung cancer.

Diseases	Lungs			Gut		
COPD	Bacteria		Fungi	Bacteria		Fungi
	Increase	Decrease	Increase	Increase	Decrease	Increase
	*Hemophilus*	*Firmicutes*	*Candida*	*Streptococcus parasanguinis_B*		*Pheumocystis*
	*Afipia*	*Acitinobacteria*	*Aspergillus*	*Streptococcus salivarius*		*Malassezia*
	*Brevundimonas*	*Streptococcus*	*Candida*	*Firmicutes*	*Acitinobacteria*	
	*Curvibacter*		*Phialosimplex*	*Prevotella*	*Bacteroidetes*	
	*Moraxella*		*Penicillium*	*Rothia*	*Roseburia*	
	*Neisseria*		*Cladosporium*	*Romboutsia*	*Lachnospira*	
	*Undibacterium*		*Eutypella*	*Intestinibacter*		
	*Corynebacterium*		*Aspregillus*	*Escherichia*		
	*Capnocytophaga*					
	*Leptolyngbya*					
Asthma	*Streptococcus*	*Bifidobacterium*	*Rhodotorula*	*Haemophilus*	Mogibacteriaceae	*Pheumocystis*
	*Bacteroides*	*Akkemrmansia*	*Candida*	*Moraxella*	Lactobacillales	*Malassezia*
		*Faecalibacterium*	*Pichia Kudriavzevii*	*Neisseria*		*Alternaria*
		*Lachospira*	*Wallemia mellicola*	*Fusobacterium*		*Cladosporium*
		*Veillonella*	*Aspergillus amstelodami*	*Porphyromonas*		*Fusarium*
		*Rothia*	*C. parapsilosis*	*Klebsiella*		
		*Ruminococcus gnavus*	*Epicoccum nigrum*	Sphingomonadaceae		
			*C. albicans*			
Lung cancer	*Prevotella*		*Aspergillus sydowii*	*Bacteroides*	*Firmicutes*	*Saccharomyces*
	*Streptococcus*		*Candida*	*Proteobacteria*	*Actinomycetaceae*	*Aspergillus*
	*Veillonella*		*Aspergillus niger*	*Enterococcus*	*Saccharobacteria*	*Apiotrichum*
	*Neisseria*		*Aspergillus fumigatus*	*Lachnospira*	*Escherichia–Shigella*	
	*Haemophilus*			*Fusobacter*	*Kluyvera*	
	*Clostridium*				*Enterobacter*	
	*Porphyromonas*				*Dialister*	
	*Megasphaera*				*Alistipes*	
	*Capnocytophaga*					

Dysbiosis of the gut microbiota, microbial translocation, and a reduction in SCFAs are associated with the severity of emphysema in rats [134]. A high‐fiber diet significantly increases the abundance of Bacteroidetes, the primary producers of SCFAs, which are the main metabolic products of the gut microbiota. SCFAs play a pivotal role in anti‐inflammatory mechanisms, including those mediated by the linoleic acid pathway, in halting the development of COPD and alleviating inflammation and alveolar damage [135]. A recent study using nontargeted fecal metagenomics and metabolomics reported significant differences in the composition of the gut microbiome and metabolome between COPD patients and healthy controls, with an increased abundance of *Streptococcus parasanguinis_B* in COPD [133]. In early studies on the acute exacerbation of chronic obstructive pulmonary disease (AECOPD), an increase in the abundance of *S. parasanguinis_B* and *Streptococcus salivarius* was found in fecal microbiota, while only *S. parasanguinis_B* was increased in sputum [136]. Furthermore, in a study comparing gut and lung microbiota, samples collected from 15 patients during a 14‐day antibiotics and steroids treatment period for COPD exacerbation, differences in the abundance of certain phyla were observed between the two sample types. However, there were no differences in the diversity of the core microbiota [130]. These studies collectively suggest that the potential crosstalk along the gut–lung axis may play a crucial role in COPD pathophysiology.

#### Asthma

Bronchial asthma is a chronic airway inflammatory disease characterized primarily by airway hyperresponsiveness as the principal pathophysiological alteration [137]. Multiple immune cells, including mast cells, eosinophils, and T lymphocytes, are involved in the pathogenesis of allergic asthma. The microbiome is a key regulator of immunity, metabolism, and cellular functions, responding to asthma‐associated inflammatory signals and potentially mediating asthma susceptibility, severity, and phenotypes [138].

Strachan's “hygiene hypothesis,” which highlights the non‐negligible role of the gut microbiome in the pathogenesis of asthma (Table 1), and Rook et al.'s “old friends theory” suggests that restoring a healthy microbial milieu could reduce the risk of allergic diseases [139, 140]. A reduction in gut microbiome diversity is associated with an increased risk of atopic diseases [141], while delayed maturation can trigger a genetic predisposition to asthma, characterized by a decrease in microbial diversity and an overgrowth of opportunistic pathogens [142]. Stiemsma et al. observed that the gut microbiota dysbiosis in children diagnosed with asthma was already evident at 3 months [143]. Colonization of the oropharynx in 1‐month‐old infants with bacteria such as *Haemophilus influenzae*, *Streptococcus pneumoniae*, or *Moraxella catarrhalis* is significantly associated with an increased risk of childhood asthma [144]. *Clostridium difficile* colonization in 1‐month‐old infants is linked to the occurrence of wheezing and asthma within the first 6 years of life. In infants at risk for asthma, genera such as *Veillonella*, *Rothia*, *Bacteroides*, and *Faecalibacterium* are typically diminished within the first 100 days after birth. Early colonization of the upper respiratory tract by the *Moraxella genus* is associated with respiratory infections, and in children aged 6 to 17 with asthma, nasal secretions show a significant activation of eosinophils, particularly in the presence of *Moraxella* colonization [145]. Asthma patients may experience alterations in mucosal immunity due to corticosteroid interference, leading to a reduction in beneficial bacteria such as *Bifidobacterium* and *Lactobacillus* and an overgrowth of pathogens [142]. Early gut microbiota may prevent asthma airway inflammation by modulating the Th1/Th2 balance [146]; murine studies have shown that commensal bacteria like *Lactobacillus*, *Bifidobacterium*, and *Bacteroides* can alleviate certain tissue inflammatory responses, including those of the airways, by inducing the production of Treg cells, reducing the release of Th1 pro‐inflammatory factors, and enhancing the production of Th2 and Th17 cytokines [147, 148]. Supplementation with beneficial gut microbiota can correspondingly improve lung diseases [148, 149]. Furthermore, feeding pregnant mice a high‐fiber or acetate diet can protect their offspring from allergic airway diseases through the Foxp3/Treg pathway [150, 151].

#### Respiratory infections

Respiratory infections have consistently posed a significant threat to global human health and incurred considerable medical expenses [152]. Germ‐free rodents exhibit weakened responses to the influenza virus [153, 154]. Mice lacking SFB in the gastrointestinal tract had higher bacterial loads, lung inflammation, and mortality rates compared to those harboring them [155]. Collectively, these findings demonstrate the influence of the gut microbiome against respiratory infections.

The misuse of antibiotics leading to dysbiosis of the gut microbiota may facilitate the translocation of potential pathogens from the intestines to the oropharynx, potentially causing respiratory tract infections [156]. A recent study of the relationship between gut microbiome imbalance and Severe Acute Respiratory Syndrome Coronavirus 2 (SARS‐CoV‐2) infection found that the gut microbiota of children with COVID‐19 had significantly higher uniformity than the control group, with an increase in the phyla Bacteroidetes and Firmicutes and a decrease in Proteobacteria. Compared with healthy controls, children with COVID‐19 had a higher relative abundance of conditional pathogens and environmental bacteria, significantly reduced bacterial diversity, and a lower relative abundance of beneficial symbionts [157]. Alterations in the composition of the gut bacterial community, rather than overall diversity, were observed in patients infected with the H1N1 influenza virus, with an increased abundance of Bacteroidetes and a corresponding decrease in Firmicutes [158]. Grayson et al. found that ingestion of the nonabsorbable antibiotic streptomycin led to a significant decrease in gut microbial diversity but no significant impact on lung microbiota; however, decreased diversity in the gut microbiome was associated with a marked increase in mortality from respiratory viral infections [159]. Oral administration of rats with metronidazole sulfate caused an imbalance in anaerobic bacteria, followed by an infection with influenza virus [160], suggesting that an imbalance in gut anaerobic bacteria exacerbates the pathological damage caused by the influenza virus. Moreover, significant differences in microbial communities were observed between patients infected with the H7N9 influenza virus and healthy individuals, with significant reductions in the abundance of the genera *Eubacterium*, *Ruminococcus*, *Bifidobacterium*, and *Lactobacillus* [161, 162].

Furthermore, a balanced gut microbiota enhances the anti‐infective ability of pulmonary tuberculosis patients and reduces the susceptibility to *Mycobacterium tuberculosis* (MTB) [163, 164]. It is currently believed that this may be due to a significant decrease in appetite of the patients, reducing the overall intake of dietary fiber, resulting in the selection of *Bacteroides* genus that can utilize host mucopolysaccharides more effectively, and reducing the number of *Prevotella* genus in the gut [165]. The relative abundance of the *Prevotella* genus positively correlating with the number of activated CD4+ and CD8+ cells indicates that the enriched *Prevotella* in the gut reflects a certain level of immune function in the body [166]. *Prevotella* can use pyruvate as a substrate to synthesize SCFAs through the acetyl‐CoA pathway, which is processed in the gastrointestinal tract and transported to the liver for metabolism [167]. The final metabolites are partially transported to the lungs through the gut–lung axis via peripheral circulation, affecting the differentiation and maturation of immune cells in the lung tissue and participating in the regulation of lung inflammation [168]. Hu et al. used metagenomic sequencing to analyze the characteristics of the gut microbiota in pulmonary tuberculosis patients and identified 39 pathways related to biosynthesis, confirming the significant changes in the gut microbiota and metabolic functions [169]. There is a trend of a significant decrease in SCFA‐producing bacteria such as *Prevotella* and a significant absence of five related pathways in pulmonary tuberculosis patients. At the same time, the decrease in the abundance of these bacteria reflects the aggravation of systemic inflammation and impairment of host immunity [170]. However, the mechanisms still await further in‐depth investigations.

#### Lung cancer

Studies have demonstrated that the gut microbiota plays a pivotal role in the pathogenesis, progression, and modulation of immune responses to therapy in lung cancer. Compared to healthy counterparts, lung cancer patients exhibit a distinct gut microbial composition with significant reductions in the Firmicutes, Actinobacteria, and Bacteroidetes and notable increases in other phyla, including Proteobacteria [171]. Multiple investigations have confirmed significant differences in the gut microbiota between lung cancer patients and healthy subjects, suggesting the possibility of gut microbiome being a biomarker for diagnosis and treatment in precision oncology [172, 173, 174, 175]. Common cancer‐associated bacteria such as *Clostridium*, *Escherichia coli*, *Fusobacterium*, *Porphyromonas*, and *Bacteroides* were identified in lung cancer patients, with an overabundance of *Clostridium* thought to activate carcinogens and be associated with tumorigenesis [176]. Lung cancer patients display dysbiosis with reduced relative abundance of Actinobacteria and *Bifidobacterium* and an increased relative abundance of *Enterococci*, with metabolomic analyses revealing a decline in the normal gut microbial functions among these patients [177, 178].

Furthermore, gut microbiota composition varies between different pathological types of lung cancer, with non‐small cell lung cancer (NSCLC) patients showing a higher relative abundance of *Rikenellaceae*, *Prevotella*, *Streptococcus*, *Lactobacillus*, *Bacteroides*, *Treponema*, and *Enterobacteriaceae* compared to healthy controls [179]. NSCLC patients also exhibited a reduction in butyrate‐producing bacteria, which are beneficial gut microbes, and butyrate produced by them can impact endothelial angiogenesis in the intestinal microvascular system, inhibiting tumor cell growth [180]. Adenocarcinoma and squamous cell carcinoma of the lungs harbor unique microbial signatures, with the genus *Gemmiger* and *Erysipelatoclostridium* enriched in the guts of adenocarcinoma patients and the genera *Enterococcus*, *Veillonella*, and *Eubacterium eligens* enriched in squamous cell carcinoma patients [181, 182]. Another study has identified and validated 13 biomarkers predictive of lung cancer, establishing a specific gut microbial signature curve for the prediction of early‐stage lung cancer (area under the curve = 97.6%) [176]. *Aspergillus sydowii* promotes lung adenocarcinoma progression by inducing myeloid‐derived suppressor cell (MDSC) expansion and activation through IL‐1*β* secretion [183]. SFB colonizing the gut can modulate CD4+ T lymphocyte polarization during pulmonary fungal infections and enhance the antifungal response of lung Th17 cells [184]. Additionally, therapeutic strategies targeting the gut microbiome are increasingly considered effective in cancer prevention and treatment improvement, with an increased abundance of *Bacteroides ovatus* and *Bacteroides xylanisolvens* enhancing the efficacy of the targeted drug erlotinib in a lung cancer mouse model [185]. The gut microbiome can regulate the effectiveness of immune checkpoint inhibitors in tumor patients [186]. *Bifidobacterium* can also recruit and activate antitumor T cells, promote the production of interferons and pro‐inflammatory cytokines, and facilitate the maturation of dendritic cells, thus enhancing the efficacy of anti‐PD‐L1 monoclonal antibody therapy [187, 188].

### Therapeutic strategies targeting the gut–lung axis

#### Fecal microbiota transplantation (FMT)

**Figure 5:**
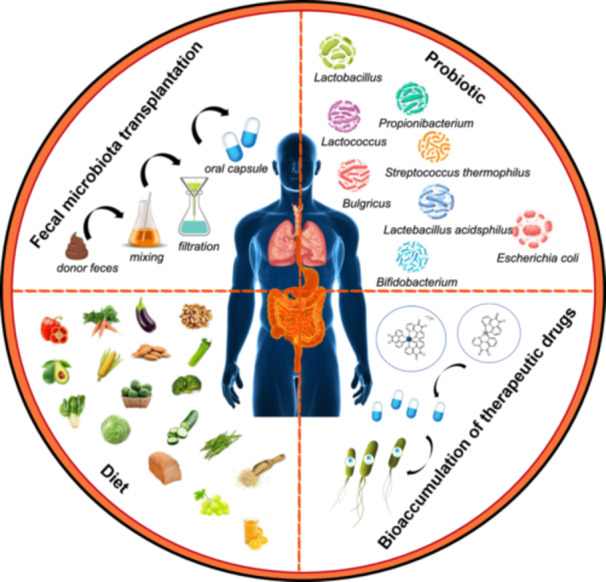
Therapeutic strategies targeting the gut–lung axis. The prospective therapeutic strategies targeting the gut*–*lung axis include FMT, probiotics, diet, and bioaccumulation of therapeutic drugs.

FMT has a promising impact on modulating the intestinal microbiota of the recipients (Table 2) having inflammatory bowel disease (IBD), irritable bowel syndrome (IBS) [189, 190, 191], or nonalcoholic steatohepatitis [192, 193]. Notably, a decrease in the symptoms of pulmonary hypertension was noted in rats following FMT administration after antibiotic treatment [194]. Interestingly, the encapsulated form of FMT is a promising approach for treating pulmonary hypertension [195]. Nevertheless, FMT bears a risk of severe infection, particularly in immunocompromised patients, with recorded incidents of aggressive *E. coli* infections; one case resulted in death. Consequently, stringent screening and testing of donors, as well as meticulous processing and preservation of FMT materials, are imperative for different disease contexts (Figure 5). Therefore, FMT application to treat diseases requires careful deliberation [195, 196].

**Table 2:** Therapeutic strategies targeting the gutlung axis.

Therapeutic strategy	Target disease	Underlying mechanisms	Effect and outcomes	PMID
FMT	COVID‐19	Intestinal epithelial cells have been identified to express ACE2 receptors. Furthermore, certain gut microbiota can enhance protection against SARS‐CoV‐2 infection through the activation of a CD8 + T cell‐mediated immune response.	The symbiotic relationship of specific gut microbial taxa presents potential for the development of a host‐directed, broad‐spectrum prophylactic strategy against COVID‐19.	36103991, 34560321, 38635321, 38408636, 38292322, 38034050
	Lung cancer	Fecal microbiota transplantation (FMT) has been shown to modulate the host's response to immune checkpoint inhibitors.	Clinical trials phase 1/2.	36177041, 38572783, 38061593, 36891304, 35735103, 35217892
	COPD	FMT alleviated hallmarker feature of COPD including inflammation, alveolar destruction, impaired lung function.	Effective in COPD Ⅲ‐Ⅳ mice.	38331563, 38459479, 32681029
	Asthma	The administration of fecal material from mice fed with either curcumin or tetrahydrocurcumin resulted in a shift in the intestinal bacterial composition, characterized by a decreased Firmicutes to Bacteroidetes ratio and lower relative abundances of pro‐inflammatory bacterial taxa such as Proteobacteria, Intestinimonas, unclassified Ruminococcaceae, and Lachnospiraceae.	FMT may have therapeutic potential for asthma treatment.	34116562, 38687096, 38513836
Diet	COPD	Fruits and vegetables are rich in vitamins C, D, and E and *β*‐carotene, all of which have antioxidant and anti‐inflammatory properties and protect against oxidative stress.	A diet with pro‐inflammatory characteristics may be associated with an elevated risk of early chronic obstructive pulmonary disease (COPD) onset and further deterioration of pulmonary function, highlighting the role of dietary interventions in promoting respiratory health and potentially mitigating COPD progression.	35889798, 3880263, 38794757, 38724980, 38687147, 38674827, 38613061, 3859152, 38531087, 38474867
	Asthma	Dietary intake of fiber‐rich foods has been associated with the suppression of airway inflammation, decreased numbers of GATA3 + Th2 cells, and a reduction in FcɛRI*α*+ eosinophils, suggesting a therapeutic potential in the management of asthma.	Asthma exacerbations may be ameliorated by dietary interventions that incorporate an increased intake of natural fibers, such as pectins.	38757128, 38844482
	Lung cancer	A positive association has been observed between the consumption of red and processed meats and an elevated risk of lung cancer incidence.	An inverse association has been reported between the dietary intake of fruits, vegetables, breakfast cereals, and fiber and the risk of lung cancer.	34582558, 34558660, 31319002
	COVID‐19	Adoption of a plant‐based diet has been associated with enhancements in immune function, including increased antibody production, lymphocyte proliferation, and a reduction in oxidative stress markers.	A plant‐based diet index has been correlated with a reduced risk of hospitalization among patients with COVID‐19.	38798209, 38744929, 38741446, 38618542
Probiotics	Lung cancer	Antimutagenic property; heavy metal detoxification; modulating immune system; managing lung diseases.	Oral probiotics supplements in combination with checkpoint inhibitors may improve the outcome in lung cancer patients.	38755052, 38736182, 38566102, 38504436, 38471270, 38336927, 38196128, 38018652, 37999101
	COPD	Immunomodulation: TGF‐*β*/IL‐4/IL‐10 (decreased); IL‐17/TNF‐*α*/IL‐2/IL‐10/IFN‐γ (increased).	Genetically modified probiotics secrete some beneficial molecules that might be utilized to treat COPD and oral probiotics prevent AECOPD.	38794746, 38635003, 38464560, 38457591, 38444395, 38249987, 37429232, 37164760, 36353491
	Asthma	Probiotic supplementation has been shown to modulate the Th1/Th2 cell equilibrium, augment regulatory T cell populations, attenuate inflammatory responses, and regulate the composition of the gut microbiota.	The adjunctive administration of probiotics has been associated with the alleviation of asthmatic manifestations, potentially through modulation of the gut microbiome and alterations in the serum metabolomic profile.	8510161, 38710644, 38687096, 38687061, 38674852, 38254421, 38235259, 38054607, 1127958, 26424567, 17204726, 32504615
	COVID‐19	*Lactobacillus rhamnosus* enhanced the T cell‐mediated immune response in infected mice; next‐generation probiotics products are linked to the regulation of intracellular calcium levels.	Probiotics have demonstrated potential in reducing mortality, ameliorating gastrointestinal and systemic clinical manifestations, and decreasing the incidence of respiratory failure in patients with COVID‐19.	34508775, 37402856, 36991513, 35487886, 32673604, 38732597

#### Diet

The dietary pattern is a critical determinant in modulating gut microbial communities (Table 2). Both clinical and experimental evidence underscores the profound effect of diet on gut microbial diversity. The swift response of gut microbiota to dietary shifts is noteworthy, with individuals experiencing marked variations in microbial composition within a day after transitioning between animal‐ and plant‐based diets [197, 198, 199]. While dietary habits are known to influence the *β* diversity of gut microbes, *α* diversity seems less affected and shows a substantial individual variation among those on diets rich in animal products [200, 201]. Epidemiological data suggests that diets high in pro‐inflammatory components are often linked to increased smoking habits, COPD, and impaired lung functions [202, 203]. Dietary components can influence the composition of the gut microbiota; for example, cheese may increase Bifidobacteria and decrease *Bacteroides* and Clostridia [204]; artificial sweeteners increase Proteobacteria and *E. coli* and decrease *Bacteroides*, Clostridia, and total aerobic bacteria [205]; foods high in polyphenols increase Bifidobacteria, *Lactobacillus*, *F. prausnitzii*, *Roseburia*, *Bacteroides vulgatus*, and *A. muciniphila* and decrease *E. coli* and *Enterobacter cloacae* [206]; and high‐fat diet increases Firmicutes and decreases Bacteroidetes [207]. Diets heavy in calories have been associated with exacerbated LPS‐induced pneumonia through disruption of gut microbiota and Th17/Treg balance [208]. Conversely, dietary fiber from fruits, grains, and cereals shows a potential protective role against lung cancer, with whole grains demonstrating a similar association [209]. It is anticipated that future preventative and supportive disease management could hinge on dietary adjustments by modifying the intake of specific foods (e.g., unprocessed dairy or fibrous foods) or lifestyle alterations (e.g., reducing sedentary behavior, obesity, and tobacco use; Figure 5).

#### Probiotics

Emerging research highlights the significance of gut microbiota alteration in managing and mitigating pulmonary conditions (Table 2). Investigations have shed light on the promising benefits of probiotic regimens in conjunction with anti‐PD‐1 treatment for progressive or relapsed non‐small cell lung cancer [210]. Probiotics stimulate immune cells (macrophages, dendritic cells, neutrophils, and NK cells) and enhance phagocytosis, cytokine release, and Th1/Th17 polarization in the gut mucosal regions [211, 212]. Conversely, certain probiotic strains help in upregulating Tregs, IL‐10, and TGF‐*β* and augmenting IgA production, as well as reinforcing the intestinal barrier by modulating dendritic cells [213, 214, 215]. Future research will pivot on the supplementation period, delivery method, dosage, and follow‐up duration for specific probiotic strains [216, 217].

#### Bioaccumulation of therapeutic drugs

Numerous pharmaceuticals tend to concentrate on specific gut bacterial species, which remain chemically unaltered by these microbes. Such bioaccumulation can decrease drug potency, alter gut microbiome composition and functionalities, and regulate the physiological state and metabolic exchanges in the host. Drug bioaccumulation by gut bacteria influences therapeutic outcomes in two primary ways: first, by diminishing drug bioavailability and, second, by altering metabolite profiles [218].

Moreover, there is a growing body of evidence indicating the effect of gut microbiota on host responses to chemotherapy and other cancer treatments, leading to three core clinical implications: enhancing drug effectiveness, negating anticancer benefits, and inducing drug‐related toxicity [219, 220, 221, 222, 223, 224]. Gut microbes have shown a strong correlation with the pharmacological impacts of various chemotherapeutic agents (like 5‐fluorouracil, cyclophosphamide, and methotrexate) and emerging immunotherapies such as anti‐PD‐L1 and anti‐CTLA‐4 treatments [188, 225, 226, 227, 228, 229]. It is anticipated that clinical practitioners and translational researchers will make substantial advancements in this domain, potentially integrating these insights into forthcoming clinical trials.

### Section summary

**Figure 6:**
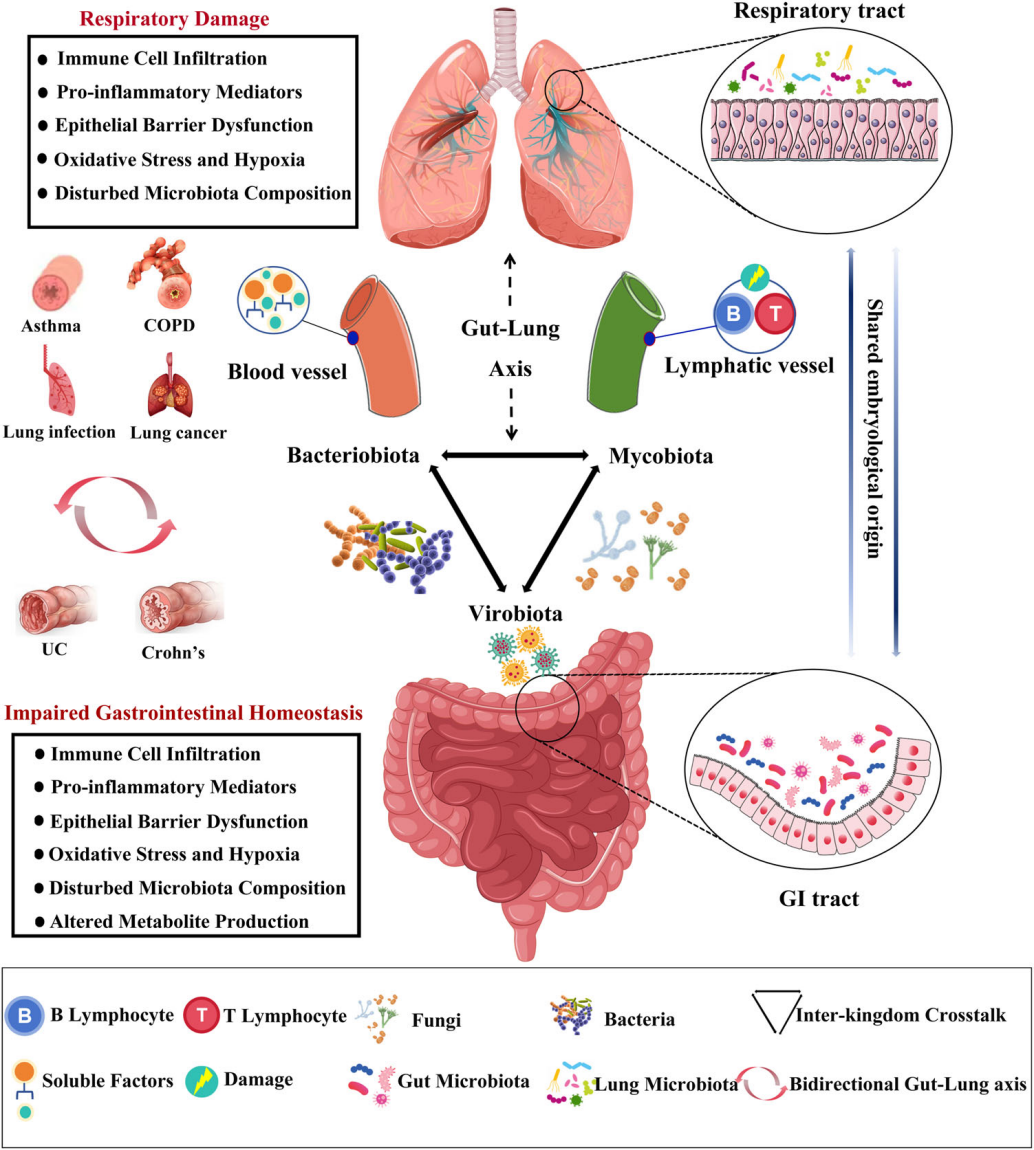
The gut*–*lung axis: A bidirectional interplay in health and disease. The gut*–*lung axis features complex interactions among bacteria, fungi, and viruses across organs. Links between the gut and lungs are evident in COPD, asthma, lung infections, and cancer. COPD involves lung inflammation, immune cell infiltration, and elevated pro‐inflammatory mediators like TNF‐*α* and IL‐6, leading to oxidative stress and hypoxia. These mediators and cigarette smoke can affect the gut, worsening immune infiltration and epithelial damage. Chronic gut inflammation alters microbiota, reducing beneficial bacteria. Impaired gut function raises pro‐inflammatory mediator levels, diminishes nutrient absorption and antioxidant capacity, and weakens pathogen defense, worsening lung diseases.

There exists a significant and intricate interplay between the gut and respiratory systems, with dysbiosis of the gut microbiota implicated in the etiology and progression of common respiratory diseases such as asthma, COPD, lung cancer, and respiratory infections (Figure 6). Interventions targeting the gut–lung axis provide effective strategies in the prevention and treatment of various respiratory diseases. Our understanding of the mechanisms underpinning the gut–lung axis is still in its infancy, and the causal relationship between pulmonary diseases and gut microbiota requires further elucidation.

As of now, research on the gut–lung axis microbiota faces two primary challenges: differentiating between causal relationships and mere associations, and accurately delineating the temporal dynamics involved. Furthermore, although culture‐independent techniques have significantly improved microbial identification, they have not obviated the need to isolate and culture potential opportunistic pathogens or beneficial probiotics for impact assessment. Many components of the microbiota remain difficult to culture effectively, complicating these analyses. As a result, discerning whether observed alterations in the microbiota are causative factors or consequences of disease processes remains challenging. Most experimental evidence to date has concentrated on the role of gut microbiota in the onset of lung diseases, with less emphasis on its impact during disease progression. This underscores the urgent need for comprehensive longitudinal studies in both human and animal models to correlate microbiota dynamics with the progression of chronic lung diseases. Investigations into microbiota interventions during lung disease are crucial for enhancing our understanding and facilitating the development of novel therapeutic strategies. Increasingly, microbiota research is focused on identifying functional clusters within microbial communities. Given the significant taxonomic diversity across various sites and among individuals, as well as the extensive range of species within the microbiota, it is likely that species overlap occurs in terms of microbial interactions and the metabolic by‐products they produce. Consequently, advanced “omics” techniques are essential to identify these functional clusters, thereby advancing our understanding of the gut–lung microbiota interplay and its collective impact on human health and disease progression.

## GUT–HEART AXIS

### Overview

Cardiovascular disease (CVD) is one of the most notable diseases threatening human health globally. CVD mainly consists of atherosclerosis, hypertension, heart failure, and cardiomyopathy. It can be influenced by multiple factors such as lifestyle, genetics, and age [230].

In recent years, gut microbiome has been shown to have a strong link to cardiovascular diseases. Similar to cardiovascular diseases, the intestinal microbiome is also influenced by diet, lifestyle, and age [231]. These influences can alter the composition of intestinal bacteria and their metabolites, which impact the cardiovascular system [232]. Hazen et al. identified an enterobacterial metabolite, TMAO, predicting cardiovascular disease risk through metabolomics [233]. When changes in the gut microbiome of aged and young mice were analyzed, the composition of the gut microbiome was found to change with increasing age. These altered microbiomes impair cardiovascular functions through TMAO [234] and aggravate cardiovascular disease. The circulating levels of TMAO correlate with age, and high TMAO levels induce an imbalance in oxidative stress, leading to endothelial senescence [235].

Thus, the gut–heart axis is an important pathway through which gut microbiome influences CVD. This axis is influenced by age, diet, and other factors affecting CVD. It is an important direction in CVD research. Herein, we discuss the latest studies elucidating the effects of gut microbiome and its metabolites on CVD development, mechanisms, and therapeutic approaches.

### The role of the gut–heart axis in various CVDs

**Figure 7:**
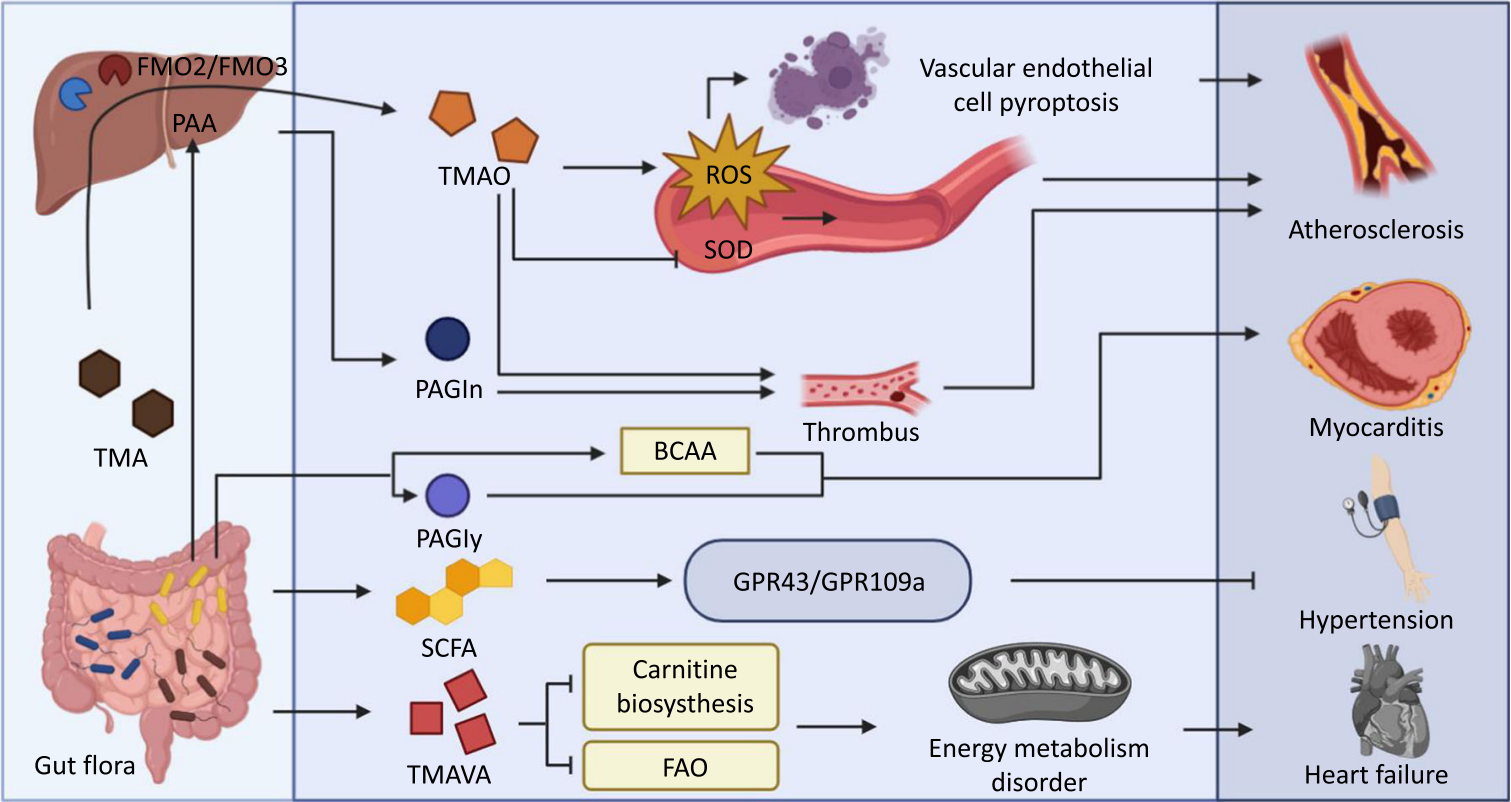
Gut microbiome metabolites (TMAO, PAGIn, SCFA, and TMAVA) influence CVD development through various mechanisms. TMA produced by the gut microbiome is converted in the liver to produce TMAO, which aggravates AS by promoting vascular endothelial cell pyroptosis through the induction of oxidative stress and the production of NLRP3. PAGIn is another gut metabolite that accelerates thrombosis and thus exacerbates AS. PAGIy and BCAA are two bacterial metabolites that are strongly associated with cardiomyopathy. In addition, SCFA produced by the gut microbiome was found to influence the progression of hypertension via GPR43/GPR109a. Finally, the bacterial metabolite TMAVA, which can lead to impaired energy metabolism and thus affect heart failure. Created with BioRender.com.

Microbiome metabolites like TMAO, phenylacetylglutamine (PAGIn), and SCFAs are strongly associated with CVD [236]. We summarize some of the mechanisms by which these metabolites, including N,N,N‐trimethyl‐5‐aminovaleric acid (TMAVA), affect CVD in Figure 7 and enumerate the studies related to the gut–cardiac axis in atherosclerosis (AS), heart failure, hypertension, and cardiomyopathy.

### Atherosclerosis

The intestinal microbiome of atherosclerosis (AS) patients has significant changes compared to healthy individuals [237]. The gut microbiome is an important regulator of AS. Gut microbiome metabolites like TMAO, PAGIn, and SCFA are the main regulators of cardiovascular effects. TMAO levels, mainly influenced by dietary and genetic factors, strongly and positively correlate with AS disease course and are involved in macrophage cholesterol accumulation and foam cell formation [233, 238]. Recently, a metabolomic approach was used to screen the three metabolites of phosphatidylcholine (TMAO, choline, and betaine) associated with CVD [233]. Dietary supplementation of phosphatidylcholine is converted into trimethylamine (TMA), the precursor of TMAO, by the gut microbiome. TMA is subsequently oxidized in the liver by the flavin monooxygenases (FMO1 and FMO3) to form TMAO, and elevated levels of TMAO accelerate AS development [238]. Treatment with TMA‐producing *L. saccharolyticum* resulted in elevated serum levels of TMAO and worsening of AS symptoms in *ApoE* ^−/−^ mice [239]. TMAO promotes AS development through a variety of mechanisms. It stimulates reactive oxygen species (ROS) production in the vascular endothelium, induces vascular inflammation, inhibits superoxide dismutase (SOD2), and activates NLRP3 inflammasomes, thereby exacerbating endothelial inflammation and contributing to AS development [240]. In addition, the pro‐AS effect of TMAO is associated with vascular endothelial cell pyroptosis by upregulating succinate dehydrogenase complex B subunit (SDHb) and causing increased ROS levels [241] (Figure 7). TMAO also affects bile acid synthesis, which inhibits intestinal cholesterol retrograde transport, leading to its accumulation in macrophages [242]. This is associated with the exacerbation of AS. Proline/serine‐rich coiled‐coil protein 1 (PSRC1) is a key factor associated with the regulation of immune responses and coronary artery disease (CAD) development. Deficiency of PSRC1 alters the abundance of intestinal TMA‐producing bacteria and hepatic FMO3 expression, which results in elevated plasma TMAO levels, exacerbating atherosclerosis [243]. However, antibiotic treatment clearing the gut microbiome diminished the effect, suggesting a gut microbiome‐dependent regulatory pathway. In addition to the microbiome associated with TMA production, some microbiome, such as *Enterobacter aerogenes* ZDY01, utilize TMA as a nutrient and reduce plasma TMAO levels [244]. It also promoted bile acid absorption and inhibited the intestinal FXR‐FGF15 axis. This promoted hepatic expression of cytochrome P450 family 7 subfamily A member 1 (Cyp7a1) gene, which accelerated the conversion of cholesterol into bile acids and facilitated reverse cholesterol transport. Thus, *E. aerogenes* ZDY01 altered plasma TMAO and cholesterol levels and reduced the extent of AS [244].

Apart from the role of TMAO in the communication between gut microbiome and AS, several other gut microbiome metabolites contribute to this process. PAGln has also been closely linked to AS. Its production is related to phenylalanine metabolism by gut microbiome converting phenylalanine via phenylpyruvate decarboxylase (PPDC) and phenylpyruvate: ferredoxin oxidoreductase (PPFOR) pathways to phenylacetic acid (PAA), which enters the liver and combines with glutamine to form PAGln [245]. PAGln is involved in the development of adverse cardiovascular events such as AS by promoting platelet reactivity and thrombosis through adrenergic receptors [246]. Similar to PAGIn, TMAO can also lead to the formation of a thrombus, thus affecting AS. SCFAs, produced by gut microbiome metabolism, are also important signaling molecules in the gut–heart axis. Propionate (PA) acts as a mitigating factor in AS by increasing IL‐10 expression and decreasing the expression of small intestinal cholesterol transporter Niemann‐Pick C1‐like 1 (NPC1L1) [247], leading to a decrease in plasma cholesterol levels. Similarly, butyrate‐producing bacteria also have a protective role in AS [248]. Thus, altering gut microbiome metabolism can ameliorate AS development through the gut–heart axis.

### Myocardial injury

Cardiomyopathy is a myocardial lesion caused by different factors, mainly myocardial hypertrophy and ventricular dilatation. It is an important risk factor for heart failure. The gut microbiome of patients with ischemic cardiomyopathy and dilated cardiomyopathy differs significantly from that of healthy individuals [249], suggesting a definite link between cardiomyopathy and gut microbiome. Both of these heart diseases are caused by myocardial lesions due to different reasons. In this research, 16S rRNA gene sequencing revealed a significant increase in strains of Proteobacteria in patients with ischemic cardiomyopathy and dilated cardiomyopathy. In contrast, previous studies have shown that Proteobacteria is strongly associated with several CVDs, including AS [250]. Current studies have not clarified the key causative organisms and pathogenic mechanisms of these two cardiomyopathies, which remains a worthy research question. In diabetic cardiomyopathy (DCM) mice, a decrease in the diversity of the gut microbiome and an increase in the abundance of Lachnospiraceae and Clostridiales, associated with the production of BCAAs, were found [251]. It has been shown that increased intestinal BCAAs in type I diabetic mice with diabetic cardiomyopathy led to increased expression of the cardiac BCAA transporter protein LAT1 via an intestinal‐hepatic‐heart axis. This results in the presence of excess BCAA in the heart, which leads to mTOR signaling‐mediated mitochondrial damage and cardiomyocyte apoptosis [252]. Myocardial ischemia/reperfusion (I/R) injury is also a risk factor for cardiac dysfunction. Myocardial I/R injury leads to gut barrier dysfunction, which increases the likelihood of bacterial translocation, leading to an increased inflammatory response, and myocardial I/R injury, which is attenuated after antibiotic treatment [253]. This evidence suggests a strong link between gut microbiome and myocardial injury.

### Heart failure

Heart failure (HF), primarily characterized by a decrease in the pumping function of the heart, can be caused by a variety of myocardial injury factors. The development of heart failure is closely linked to the gut microbiome. Heart failure causes the heart to pump less blood, leading to gut ischemia. Reduced intestinal blood flow affects nutrient absorption and causes gut dysfunction [254]. Using 16S rRNA gene sequencing, a reduction in intestinal SCFA and tryptophan‐producing bacteria was observed after cardiac stress loading in mice. Dysregulation of the gut microbiome caused cardiac remodeling and dysfunction in a T cell‐dependent manner, whereas tryptophan and cardiac AhR exhibited significant cardioprotective effects [255].

Microbial‐derived metabolites are also strongly associated with the progression of heart failure. TMAO level is strongly associated with the prognosis of heart failure [256]. In a mouse model, TMAO induced myocardial fibrosis and cardiac hypertrophy through the TGF‐*β*1/Smad3 pathway [257], suggesting a role for TMAO in heart failure. Myocardial hypertrophy is an important risk factor for heart failure. In patients with heart failure, the level of the intestinal epithelial secretory factor, FGF19, is elevated, which promotes myocardial hypertrophy, causing heart failure [258]. TMAVA has also been associated with the prognosis of patients with heart failure. TMAVA inhibits carnitine synthesis and fatty acid oxidation (FAO), which leads to metabolic reprogramming of the cardiomyocytes and causes impairment of myocardial energy metabolism [259] (Figure 7). Similar to TMAVA, TMAO can cause mitochondrial dysfunction and impaired myocardial energy metabolism, leading to the development of heart failure [260]. Heart failure with preserved ejection fraction (HFpEF) is one type of heart failure. Using metabolomics, reduced levels of indole‐3‐propionic acid (IPA) in a mouse model of HFpEF were observed, suggesting a potential protective role for IPA in HFpEF. The heart failure‐protective effects of IPA are mainly mediated by AhR stimulation of sirtuin3 (SIRT3) expression as well as inhibition of NNMT (nicotinamide N‐methyltransferase) with an increase in NAD+ levels [261]. In addition, gut microbiota‐derived kynurenine, a metabolite associated with heart failure, also contributes to the progression of myocardial fibrosis through AhR activation [262]. Myocardial fibrosis after myocardial infarction is a risk factor for heart failure, and butyrate‐producing bacteria in the gut play an important role in this process. Butyrate inhibits histone deacetylase (HDAC) activity, which promotes tissue repair after myocardial infarction [263]. However, the reduction of butyrate‐producing bacteria promotes myocardial fibrosis and cardiac dysfunction after myocardial infarction.

### Hypertension

Hypertension is a multifactorial cardiovascular disease. A recent study compared the gut microbiome composition in 41 healthy controls, 56 prehypertension patients, and 99 essential hypertension patients. The results showed a reduced abundance of SCFA‐producing bacteria *F. prausnitzii* and *Roseburia* in prehypertensive and hypertensive patients. Hypertensive patients' feces‐transplanted germ‐free mice developed elevated blood pressure [264], indicating an important role for the gut microbiome in hypertension development [265]. High‐salt diet is one of the risk factors for hypertension. The abundance of *Lactobacillus murinus* is reduced in people and mice given a high‐salt diet [266]. *Bacteroides fragilis* inhibited high salt diet‐induced intestinal‐derived corticosterone production through its metabolite arachidonic acid, which attenuated hypertension symptoms [267]. Modulating steroid hormones could be one of the ways how gut microbiome affects hypertension. In addition, other gut microbiome metabolites like TMAO and SCFA also affect hypertension symptoms. Plasma TMAO levels are elevated in hypertensive patients, and TMAO contributes to Ang II‐induced hypertension by promoting vasoconstriction [268]. SCFAs are produced by the fermentation of fibers by the gut microbiome, and there is a close link between them and the cardiovascular system. The cardioprotective effects of SCFAs are mediated through their receptor GPR43/GPR109a, and the reduction of SCFAs leads to a weakening of this signaling pathway causing hypertension [269]. Thus, the link between SCFAs in feces, SCFAs absorbed into the bloodstream, and cardiovascular disease deserves further clarification. In addition, cardiovascular regulation by gut microbiome involves modulation of the immune responses. Celiac disease (CeD) is an autoinflammatory bowel disease that leads to IL‐17 release from intestinal cells and activation of NLRP3 inflammasomes [270]. The entry of such inflammatory factors into the circulatory system leads to several problems, including increased cardiovascular risk and arterial hypertension.

### CVD treatment strategies based on the gut–heart axis

Currently found therapeutic means for this axis are mainly through the dietary approach, probiotics, drugs, and other factors affecting the gut microbiome and its metabolites to achieve the purpose of treating cardiovascular diseases. Dietary modifications are one of the factors that influence the gut microbiome composition and the production of metabolites. It is well known that high‐fat and high‐salt diets are important risk factors for cardiovascular disease. Treatment of cardiovascular diseases by improving diet is promising because of the cardiovascular‐protective properties of the Mediterranean diet [271]. The intake of high‐fiber foods coupled with the metabolic effects of the gut microbiome leads to an increase in the production of several SCFAs with cardioprotective effects. After high‐fiber food feeding, the abundance of acetate‐producing bacteria in the intestine of mice was increased, and symptoms of cardiac hypertrophy and hypertension were ameliorated [272]. Moreover, cardiac hypertrophy symptoms and hypertension were also significantly improved in mice through acetate supplementation [272]. Similarly, exogenous supplementation with propionic acid has significantly improved aortic aneurysm development [273]. In addition to altering ingested foods, fasting was proven to be cardiovascular protective. Intermittent fasting (IF) reduces blood pressure by altering the gut microbiome, and the effect is mainly mediated through bile acid signaling [274]. The effects of fasting also include immunomodulatory effects on Th1 cells and dendritic cells, which exert a blood pressure‐lowering effect [275]. Dietary Approaches to Stop Hypertension (DASH) diet is currently an internationally recognized diet for the treatment of hypertension. The DASH diet has a better antihypertensive effect when combined with fasting [276].

Probiotics are used to produce cardiovascular protective effects, mainly by altering the gut microbiome composition. *Lactobacillus rhamnosus* and *Bifidobacterium lactis* are two probiotics with antihypertensive effects. They exert their blood pressure‐lowering effects by affecting lipid metabolism, vascular smooth muscle contraction, and steroid hormone synthesis [276]. Treatment of hypertensive rats with *Lactobacillus* has significantly decreased blood pressure [277]. Interestingly, through fecal histology studies, *F. prausnitzii* was found to be significantly associated with CVD [278], and it has significant anti‐atherosclerotic effects [278]. Therefore, *F. prausnitzii* supplementation is a promising probiotic therapy for atherosclerosis. *A. muciniphila* is significantly reduced in mice with aortic aneurysms, and its oral administration attenuated aortic aneurysm development [279]. The main mechanisms are inhibition of inflammation, restoration of structural diversity of the gut microbiome, and regulation of *Lactobacillus* functions [279]. Oral administration of *Saccharomyces boulardii* improved the ejection function of the heart in patients with heart failure [280].

### Section summary

The gut–heart axis is a signaling axis for the interactions between the gut microbiome and CVD. The development of CVD affects the composition of the gut microbiome, and in turn, the gut microbiome modulates CVD progression. In a healthy condition, the gut microbiome and cardiovascular system maintain homeostasis, which is disrupted in the presence of certain pathogenic factors like high‐fat, high‐salt diets and smoking, leading to the development of various CVDs. Gut microbiome affects CVD development mainly through its metabolites, immunomodulation, and hormonal regulation. Gut microbiome and its metabolites could be either beneficial or harmful. For example, TMAO is harmful to the cardiovascular system, while SCFAs are cardioprotective. Therefore, investigating how gut microbiome regulates CVD will help in the development of novel therapeutic strategies. The main treatments for CVD targeting the gut–heart axis include dietary interventions and probiotic supplementation. This would allow for the intervention of CVD in patients through dietary improvements or probiotic beverages. However, most of the probiotics or medications currently available for CVD are in the research or clinical trial stage. Therefore, further research is needed to determine the effectiveness and safety of these interventions. The development of therapeutic strategies for CVD targeting the gut–heart axis is important and promising.

## GUT–BONE AXIS

**Figure 8:**
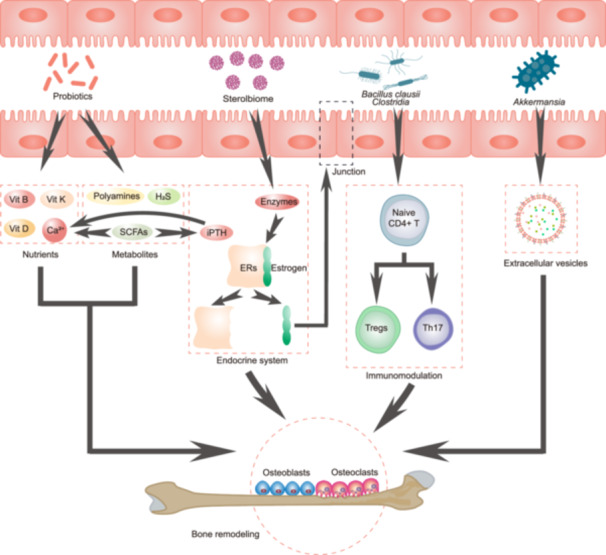
Potential mechanisms by which gut microbiota regulates bone metabolism. The gut microbiota, particularly probiotics, promotes the production and/or absorption of nutrients (such as calcium, and vitamins D, B, and K) to maintain bone health. Additionally, the gut microbiota generates short‐chain fatty acids (SCFAs), polyamines, hydrogen sulfide (H_2_S), and extracellular vesicles; influences the balance between Tregs and Th17 cells; releases estrogen from estrogen receptors (ERs) through sterolbiome enzymes; and mediates the effects of intermittent parathyroid hormone (iPTH) via SCFAs. All of these mechanisms regulate bone remodeling by affecting the differentiation of osteoblast and osteoclast precursors, ultimately modulating bone metabolism.

Bone homeostasis relies on an equilibrium between bone‐resorbing osteoclasts and bone‐forming osteoblasts, a process known as “bone remodeling” [281]. Various factors contribute to osteoporosis, with one of them being the gut microbiota [282]. The gut microbiota regulates bone metabolism by influencing multiple bone‐related factors to operate the “gut–bone” axis [283] (Figure 8).

### Gut microbiota is closely related to bone

For over a decade, the gut microbiota has been found to be closely related to bone. Germ‐free mice exhibit a higher bone mass and fewer osteoclasts than conventionally raised mice; introducing normal gut microbiota normalizes the increased bone mass [284]. Moreover, gut microbiota depletion improves bone mass, bone microstructure, and bone strength in ovariectomized (OVX) mice. This effect is mediated by the G‐protein‐coupled bile acid receptor [285]. In the China Multi‐Ethnic Cohort study, “altitude‐microbiota‐quantitative ultrasound index” analysis revealed a negative correlation among high‐altitude exposure, gut microbiota, and bone mineral density (BMD); additionally, it identified a mediating effect of *Catenibacterium* in the correlation [286]. A Mendelian randomization analysis using data from the TwinsUK, LifeLines‐DEEP, and UK Biobank cohorts showed a causal link between gut microbiota and bone development, highlighting a specific causative bacterial taxa (order_Clostridiales and family_Lachnospiraceae) [287]. Other reviews have explored the relationship between gut microbiota and bone [288, 289], which will not be further elaborated here.

### Potential mechanisms by which gut microbiota regulates bone metabolism

#### Nutrient absorption

Modification in the abundance, diversity, and composition of gut microbiota impact dietary intake, particularly nutrient absorption, and subsequently influences bone metabolism via the gut–bone axis [290]. For instance, calcium plays a critical role in maintaining optimal bone health in humans [291]. Supplementation with probiotics, especially *Lactobacillus* strains, enhanced calcium transport and uptake in mice [292]. Moreover, a human trial involving postmenopausal women revealed an increase in serum calcium levels after consuming *Lactobacillus helveticus* fermented milk [293]. Supplementation with galactooligosaccharides (prebiotics) heightened calcium and magnesium absorption with an improved BMD [294]. The mechanisms through which probiotics and prebiotics enhance calcium absorption and bioavailability include the upregulation of calcium transporter expression, increased cell density, intestinal crypt depth, and blood flow, as well as alterations in the intestinal microbiota composition and integrity [291].

The gut microbiota also regulates vitamin D metabolism, which in turn significantly influences the absorption of calcium and phosphorus in the gut, as well as bone metabolism [295]. Germ‐free mice exhibit impaired vitamin D metabolism, but after gut microbiota colonization, levels of 1, 25‐dihydroxy vitamin D and calcium were restored [296]. Oral supplementation with probiotic *Lactobacillus reuteri* increases circulating levels of 25‐hydroxyvitamin D [297]. Vitamin D receptor is prominently expressed in the gastrointestinal tract, where vitamin D activates its receptor to maintain the intestinal epithelial barrier and promotes gut microbiota eubiosis [298].

The gut microbiota members are essential for synthesizing vitamins B and K as well [299], both of which are vital for maintaining bone health [300]. Vitamin K is instrumental in bone metabolism by contributing to the γ‐carboxylation of tissue‐specific vitamin K‐dependent proteins like osteocalcin [301], which stimulates the xenobiotic receptor on osteoblasts and influences bone remodeling and bone mineralization processes, ultimately leading to an increased bone strength [302]. Inadequate intake of vitamin B has been observed in hip fracture patients; several observational studies have also demonstrated a link between various B vitamins (B2, B6, folate, or B12) and a reduced risk of osteoporosis or hip fractures [303].

#### Metabolites

The gut microbiota beneficially influences distal organs by generating metabolites known as “postbiotics.” Changes in gut microbiota‐related metabolites are associated with osteoporosis development and progression [304]. For example, the gut microbiota ferments indigestible carbohydrates in the diet, leading to the production of SCFAs (propionic acid, butyric acid, and valeric acid) in the intestinal lumen [305]. The SCFAs can lower pH in the intestine, thereby increasing the solubility and subsequent absorption of minerals like calcium, phosphorus, and magnesium. Propionate and butyrate reduce the expression of osteoclast‐related genes TRAF6 and NFATc1, suppress osteoclast differentiation and bone resorption, and notably enhance bone mass in OVX mice [306]. Moreover, valeric acid serves as a protective agent against postmenopausal bone loss by impeding the NF‐κB signaling pathway to inhibit osteoclast differentiation [307].

Polyamines, another kind of metabolite, are mainly synthesized by the gut microbiota through transamination of amino acids, particularly arginine, with the aid of catalytic enzymes [308]. The polyamines enhance the expression of osteogenic genes, such as alkaline phosphatase (ALP), runt‐related transcription factor 2 (RUNX2), osteopontin, and osteocalcin (OCN), thereby promoting extracellular matrix mineralization and osteogenesis in human bone marrow‐derived mesenchymal stem cells (BMSCs) [309]. Furthermore, polyamines act as inhibitors of osteoclastogenesis; for example, regular consumption of polyamine‐rich *Saccharomyces cerevisiae* S631 prevents osteoclastic activation and bone loss in OVX mice [310]. Arginine, as one of the precursors for polyamine synthesis, is associated with the “gut–bone” axis [311]. Lachnospiraceae contributed to bone mechanoadaptation in mice by producing l‐citrulline and converting it into l‐arginine [311]. The l‐arginine boosts bone mechanoadaptation by activating a positive feedback loop involving nitric oxide and calcium in osteocytes [311].

Hydrogen sulfide (H_2_S) is another vital metabolite involved in gut microbiota‐mediated bone remodeling. It is generated through the breakdown of cysteine by sulfate‐reducing bacteria [312]. H_2_S is essential for bone formation and development of the postnatal skeleton, as it supports the self‐renewal and osteogenic differentiation of BMSCs via the Wnt/*β*‐catenin signaling pathway. Insufficient levels of H_2_S result in a persistent osteoporotic phenotype characterized by compromised BMSCs and impaired bone formation [313].

### Immunomodulation

The communication between the gut microbiota and the host immune system starts during infancy. According to the theory of “Osteoimmunology” [314, 315], a balance between Tregs and Th17 cells is crucial for maintaining bone homeostasis. Treg cells suppress osteoclast formation from monocytes and induce CD8^+^ T cells to generate the Wnt ligand, Wnt10b, which promotes osteoblast generation [316]. In contrast, Th17 cells induce pro‐inflammatory cytokines and inhibit anti‐inflammatory factors, which promote osteoclast formation and thus increase bone loss [316].

The gut microbiota plays a crucial role in influencing the differentiation of naive CD4^+^ T cells into Tregs or Th17 cells, thereby regulating bone metabolism [316]. For example, *Bacillus clausii* significantly enhances the population of CD4^+^ Foxp3^+^ Treg cells while reducing the proportion of CD4^+^ Rorγt^+^ Th17 cells in the bone marrow, thereby preventing bone loss induced by OVX in mice [317]. On the other hand, SFB promotes the expansion of Th17 cells and the production of IL17 in the gut [318], while Clostridia has shown significant advantages in enhancing Treg cell populations [319]. In addition, various bacterial species have the potential to modulate the Tregs and/or Th17 cells, as mentioned in other published reviews [283, 320].

### Endocrine system

The term “sterolbiome” describes a cluster of gut microbiota that alters cholesterol‐derived compounds, thereby playing a direct role in regulating the levels of sex steroids in the host [321, 322]. The sterolbiome contains enzymes such as *β*‐glucuronidase, *β*‐glucosidase, hydroxysteroid dehydrogenase, and sulfatase, which release estrogen from its receptors and enhance its reabsorption in the gut; consequently, the gut microbiota modulates estrogen metabolism and impact both local and systemic estrogen levels [283]. Estrogens have a direct and indirect role in regulating bone metabolism and functions [323]. Lack of estrogen results in gut microbiota‐driven reduced expression of intestinal tight junction proteins, increased intestinal permeability, and inflammation [324].

Parathyroid hormone (PTH) is also essential for postnatal skeletal development as it regulates calcium balance [325]. Continuous parathyroid hormone (cPTH) is a prevalent factor contributing to osteoporosis and fractures, mediated by interactions with the gut microbiota. SFB facilitates cPTH to amplify the inflammatory effects, leading to elevated levels of TNF^+^ T cells and Th17 cells in the intestine. These cells subsequently migrate from the intestine to the bone marrow, ultimately contributing to bone loss [326]. On the contrary, intermittent parathyroid hormone (iPTH) induces bone anabolism effects, which depend on the gut microbiota‐produced butyrate. Through its binding to GPR43 on dendritic cells, butyrate facilitates the differentiation of Tregs; subsequently, the Tregs promote Wnt10b expression in CD8 + T cells within the bone marrow, leading to the activation of Wnt‐dependent mechanisms promoting bone formation [327].

### Extracellular vesicles

Extracellular vesicles (EVs) released by bacteria serve as a form of inter‐species communication and display distinct characteristics [328]. The gut microbiota acts on bones through these vesicles [283]. For example, introducing gut microbiota from children or the bacterium *A. muciniphila* prevents OVX‐induced osteoporosis in mice. This protective effect is facilitated by the secretion of EVs [329], which penetrate and gather in bone tissues, suppressing osteoclastogenesis and stimulating osteogenesis [329]. Similarly, EVs from *Proteus mirabilis* protect against bone loss by promoting mitochondria‐dependent apoptotic pathways in osteoclasts [330].

### Probiotics and osteoporosis therapy

Probiotics confer positive health effects when consumed in sufficient amounts. Numerous studies in animal models have demonstrated the potential of probiotics as a therapeutic approach for osteoporosis [331]. For example, products fermented with *Lactobacillus*, such as kefir and soy skim milk, positively impact bone health [332]. *Bifidobacterium* prevents OVX‐induced bone loss by hindering the differentiation of pre‐osteoclasts in vitro and expediting the remodeling of callus cartilage in mice with fractures [333, 334].

Only a few studies have reported that probiotics can directly or indirectly influence bone metabolism in humans. For example, supplementation with calcium and short‐chain fructo‐oligosaccharide (a type of prebiotic) in postmenopausal women has been shown to reduce levels of C‐telopeptide of type I collagen (CTX‐I), a marker of bone turnover that reflects bone resorption [335]. *Lactobacillus reuteri* has been found to reduce bone loss in older women with low BMD [336]. Additionally, *Lactobacillus casei Shirota* in milk can improve fracture‐related symptoms (such as grip strength, pain, and active range of motion) in elderly patients with distal radius fractures [337]. Furthermore, multispecies probiotic supplementation has favorable effects on bone biomarkers, leading to decreased levels of bone‐specific alkaline phosphatase, CTX‐I, TNF*α*, and PTH in osteopenic postmenopausal women [338].

### Future prospects

The effects of gut microbiota on bone health are influenced by factors such as the host's gender, aging, menopause, and growth [339]. Therefore, integrated analyses and machine learning using multiomics data will provide opportunities to explore the mechanisms by which gut microbiota affects bone health and to advance precision medicine in this area.

Additionally, several novel gut microbiota‐related therapies (e.g., FMT, postbiotics, next‐generation probiotics, and resistant starch) have been reported to be associated with osteoporosis treatment in animal models. For instance, administering postbiotics (cell lysates and supernatants derived from probiotics) to OVX rats can help prevent bone loss [340]. Polysaccharides from resistant starch could enrich the gut microbiota to regulate bone metabolism [341]. *A. muciniphila*, recognized as a next‐generation probiotic, has been linked to bone physiology and bone formation [329]. These approaches warrant further exploration for their potential application in osteoporosis patients in the future.

## GUT–SKIN AXIS

### Overview

Microorganisms colonizing the gut and skin play a key role in barrier homeostasis, and gut microbial disorders affect the health of the skin. Herein, we discuss the relationship between the gut microbes and the skin and summarize the effect of gut microorganisms on different skin diseases such as atopic dermatitis (AD), psoriasis (Ps), acne vulgaris, rosacea, dandruff and seborrheic dermatitis, hidradenitis suppurativa, and skin cancer. We reviewed the different mechanisms by which the gut affects the skin to improve our understanding of the gut–skin axis. Finally, we provided an outlook on how to improve skin health by regulating gut microbes based on existing findings, which will inspire the treatment of skin diseases in the future.

### Skin and microbiome

The skin, as the largest organ of the organism, serves as a vital interface with the external environment. It encompasses the entire external surface of the organism and establishes connections with the digestive system through oral and anal mucous membranes [342]. It is primarily composed of two layers: epidermis and dermis. The epidermis consists of five layers of keratinocytes: the basal layer, the stratum spinosum, the stratum granulosum, the stratum pellucidum, and the stratum corneum. The dermis is mainly fibrous‐collagenous‐elastic tissue [343]. The skin is the first line of defense against infections and injuries [344]. It possesses a powerful filtering effect, with the surface colonized by commensal microbiota inhibiting the invasion of pathogens [345]. The integrity of the skin and its appendages is the key to maintaining skin barrier homeostasis and preventing infections [346]. As a barrier between the body's internal and external environments, the skin is affected by external factors such as air pollution, ultraviolet rays, sanitary conditions, and so forth, and it is also affected by internal factors such as inflammation, trauma, aging, and systemic diseases [347, 348, 349].

Skin symbiotic microbiota change over time, with neonatal skin microorganisms changing rapidly during the first 6 months of life and slowing down after 12 months. During puberty, the skin microbiome changes considerably due to the action of sex hormones until it stabilizes in adulthood. Adult skin microorganisms are relatively stable and vary greatly from site to site. Skin microbes vary significantly between individuals with different body mass index (BMI) and dietary habits, which may affect the therapeutic effects of skin diseases [350, 351, 352, 353, 354, 355, 356, 357, 358, 359, 360, 361, 362, 363, 364, 365, 366]. Therefore, the effect of the gut on skin microbial homeostasis may provide new clinical solutions for the treatment of skin diseases.

**Figure 9:**
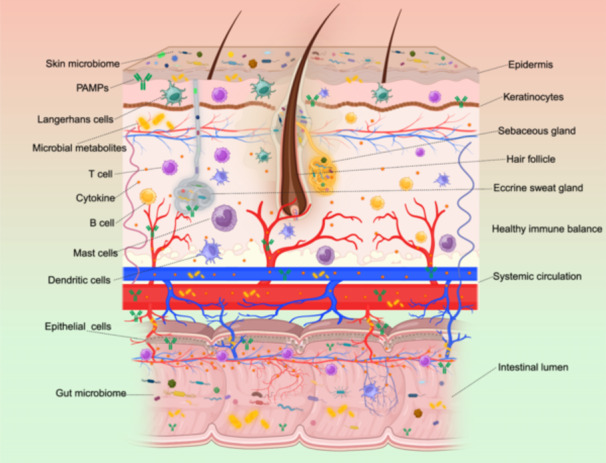
The crosstalk between gut and skin. The gut and skin epidermal barriers are connected through systemic circulation. Gut (gut microbiome and gut inflammation) and skin (immune imbalance, skin microbiome, and proliferation of keratinocytes) dysbiosis are interconnected and reciprocal.

**Figure 10:**
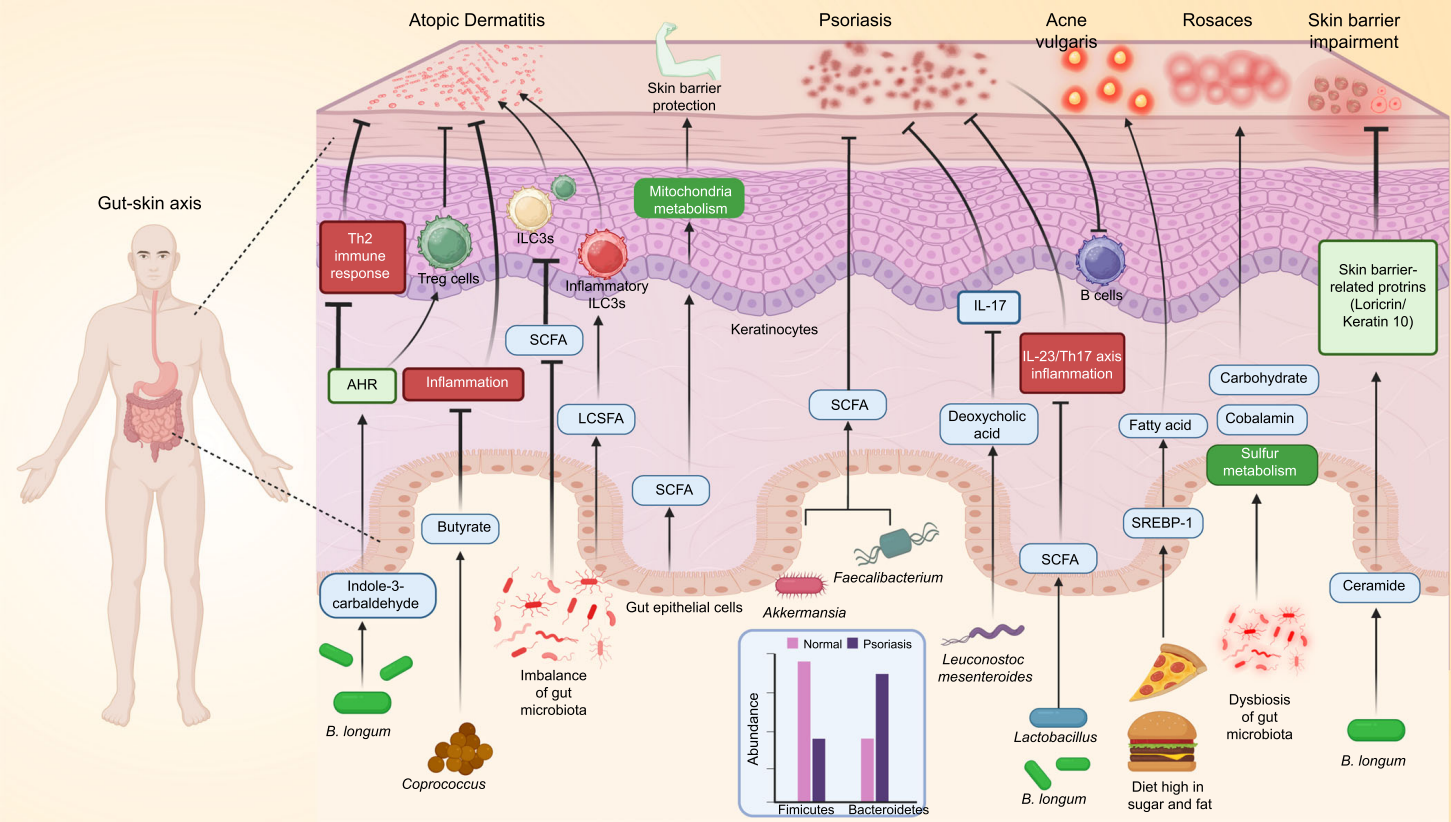
Links between skin diseases and the intestinal system. Detailed summaries are described below in the text, categorized by different diseases (AD, psoriasis, acne vulgaris, rosacea, and other skin barrier impairment).

Both gut and skin are rich in blood vessels and neural tissues, facilitating communication between the body and the external environment [367]. In clinical practice, gastrointestinal diseases often coincide with skin disorders [368, 369]. Similarly, conditions like psoriasis may be linked to comorbidities like obesity or IBD [370, 371, 372, 373]. Damage to the intestinal barrier leads to the colonization of pathogenic microorganisms, and their metabolites affect the skin's epidermal barrier through circulation [361, 374, 375, 376, 377, 378, 379]. This interconnected and intimate relationship between the gut microbiota and skin is known as the “gut–skin axis” (Figures 9 and 10).

The gut microbiota consists of bacteria, fungi, viruses, and protozoa [380]. Both internal and external factors influence the balance of individual gut microbiota homeostasis, which subsequently affects the health of the skin [381]. For instance, diet affects the host's skin microbiota by influencing the abundance of certain gut microbes [382]. Exogenous supplementation of probiotics ameliorates skin barrier damage caused by excessive phenols produced from gut microbial disorders [361]. Consuming high‐fat diets or alcohol disrupts the equilibrium of gut microbes and causes impaired skin wound healing [383]. Moreover, *Bifidobacterium longum* modulates tryptophan metabolism and attenuates atopic dermatitis (AD) symptoms. It also contributes to ceramide synthesis and ameliorates skin barrier damage in aging mice [384, 385].

Skin microorganisms consist of bacteria, fungi, viruses, and phages [386]. Each individual's skin hosts a unique microecology [387], which affects skin structure and functions without penetrating the epidermis [388]. In recent years, there has been a growing focus on the role of skin microbes in preserving the skin barrier [389, 390, 391], and several skin diseases can be exacerbated by the imbalance in gut microbes [392, 393, 394]. However, further research is necessary to comprehend the intricate interplay between the gut and skin microbiota.

AD patients exhibit changes in gut microbial diversity and species composition, characterized by a decrease in probiotics and an increase in pathogenic bacteria [395]. Supplementation with exogenous *Bifidobacterium longum* enhances indole‐3‐carbaldehyde release, which activates AhR receptors. This activation suppresses aberrant Th2 immune responses and alleviates AD symptoms [384]. AhR receptor activation also induces oxidative stress and regulates Tregs. Targeting AhR receptor activation is a strategy employed for the treatment of AD [396]. In addition, an excessive intake of fatty acids along with reduced consumption of fruits and vegetables affects AD progression, influencing Treg cells and Th2‐type immune responses [397]. The reduced abundance of *Coprococcus* in the gut of AD patients results in a decrease in butyrate production and its anti‐inflammatory effects [398]. Dysbiosis of gut microbiota caused by antibiotics exacerbates AD by reducing the production of SCFAs, resulting in fewer Treg and ILC3 infiltration [399]. Conversely, long‐chain saturated fatty acids (LCSFA) induce an increase in inflammatory ILC3s in the intestine, which migrate to the skin and trigger AD [400]. Furthermore, SCFAs derived from the gut enhance mitochondrial metabolism in keratinocytes, thereby protecting the skin barrier in experimental AD [401].

Psoriasis, a systemic disease characterized by scales and thickening of the skin at any site, is classified as a Th17‐mediated disorder [402]. Apart from skin lesions, inflammation also occurs in other organs. For example, psoriasis patients often show a higher BMI and have hypertension, type 2 diabetes, or IBD [370]. Dysbiosis of gut microbes may trigger immune responses, leading to susceptibility to psoriasis. Psoriasis patients exhibit an increased abundance of Firmicutes and a decreased abundance of Bacteroidetes in their gut [403]. A decreased abundance of *Akkermansia* and *Faecalibacterium* in psoriasis patients leads to reduced production of SCFAs, which function as anti‐inflammatory factors and maintain the integrity of the epithelial barrier [404, 405]. Supplementation with *Leuconostoc mesenteroides* decreases IL‐17 production and alleviates psoriasis by increasing serum deoxycholic acid. Intake of *Bifidobacterium* or *Lactobacillus* produces SCFAs and inhibits the IL‐23/Th17 axis‐associated inflammatory factors [406]. In addition, psoriasis decreases gut B‐cells in mice, causes dysbiosis in the gut, and exacerbates enteritis [407].

Acne vulgaris is a multifactorial disease associated with diet, host immune status, and skin microbial homeostasis. The occurrence of acne vulgaris is highly correlated with dietary factors. Acne vulgaris patients have reduced gut microbial diversity and species abundance [408]. Moderately severe acne is strongly associated with a high‐fat and high‐sugar diet. This connection may be explained by the activation of sterol regulatory element‐binding protein 1 (SREBP‐1) due to high fat and glucose, leading to increased fatty acid synthesis in sebum. Therefore, disruption of gut microbes caused by dietary factors affects the progression of acne vulgaris [409, 410].

Rosacea is a chronic inflammatory skin disease characterized by neurovascular dysregulation and impaired immune functions [411]. The main causes are the release of abnormal inflammatory factors and antimicrobial peptides. There is a certain correlation between rosacea and gut microorganisms, as patients with rosacea are more susceptible to *Helicobacter pylori* infection, which in turn contributes to the development of gastrointestinal disorders [412]. Moreover, gut microbes' abundance is reduced in rosacea patients, which may affect the disease progression through sulfur metabolism, cobalamin utilization, and carbohydrate transport [413]. Specific foods and beverages exacerbate rosacea symptoms, possibly due to the activation of transient receptor potential cation channels, leading to neurogenic vasodilation [414].

The association between dandruff, seborrheic dermatitis, and gut dysbiosis is a topic to debate, with some evidence suggesting that a diet high in fat and sugar may worsen the scalp condition [415]. Additionally, probiotic supplementation alleviates dandruff and seborrheic dermatitis [416]. Diet‐induced imbalance of gut microorganisms is associated with the progression of hidradenitis suppurativa [373], which can lead to IBD development [417]. Ceramide produced by *Bifidobacterium longum* enhances the skin barrier integrity in aging mice via the gut–skin axis [385]. Furthermore, dietary modifications and probiotic supplementation have beneficial effects on melanoma immunotherapy outcomes [418]. Interestingly, coffee consumption is inversely associated with the risk of skin basal cell carcinoma development [419]. The role of gut microbes in these processes warrants further investigation.

### Clinical applications

Skin health can be regulated by improving gut microbes, and the consumption of probiotics and prebiotics for efficient repairing of the skin barrier has been validated in cohort studies [420]. Additionally, a healthy diet is beneficial for skin diseases [421]. Recent clinical investigations have demonstrated a significant alleviation in AD symptoms after FMT [422], and feeding probiotics resulted in better hair quality in mice [423]. Hence, targeting gut microbiota represents a novel approach to managing skin issues.

The gut–skin axis offers a novel perspective on the pivotal role of gut microbes in skin health. In the future, employing specific microbial metabolite tagging techniques in mouse skin will yield compelling evidence regarding the direct impact of gut microbes on skin physiology. Meanwhile, the application of identified probiotics and prebiotics in daily skin care products has immeasurable clinical translational value. For example, a variety of metabolites of gut microbes are currently being added to cosmetic products [424, 425, 426]. Further clarification of the specific roles of gut microbes in the skin barrier and making personalized therapeutic measures for skin health management will have a very promising clinical application in the near future.

In conclusion, exploring the factors influencing skin health, clarifying the specific mechanisms for achieving improved skin health through modulation of gut microbes, and translating these findings into specific therapeutic recommendations to unleash the regenerative potential of the skin will have far‐reaching implications for the clinical management of skin problems.

## GUT–REPRODUCTION AXIS

### Overview

The homeostasis of the gut microbiota plays a pivotal role in reproductive health by interacting with sex hormones like estrogen at every stage and level of female reproduction. Anomalies in the composition and functions of the microbiota contribute to the onset and progression of many reproductive diseases; these include polycystic ovarian syndrome, premature ovarian insufficiency, endometriosis, and pregnancy‐associated complications. Even though the associations between perturbations of the gut microbiota and reproductive disorders have been demonstrated within numerous studies, cause–effect relationships between gut microbiota dysbiosis and reproductive dysfunction remain elusive. Future studies need to be undertaken that would unveil the precise pathologic effects of gut microbiota dysbiosis and the molecular mechanisms underlying the association between imbalanced gut microbiota and reproductive diseases, which would greatly facilitate the development of novel therapeutic and preventive strategies.

### Human gut–sex hormone axis

#### Human gut–estrogen axis

The gut microbiota is crucial to the regulation of hormonal levels in the host, especially estrogen. The robust link between the gut microbiome and estrogen levels is confirmed by the use of antibiotics, which significantly decreased circulating estrogen levels in women [427]. Moreover, the gut microbiota participates in the regulation of estrogen levels through the estrobolome, characterized by the genes that encode estrogen‐metabolizing enzymes in the gut microbiome. The *β*‐glucuronidase enzyme secreted from gut microbiota metabolizes conjugated estrogens to deconjugated forms, affecting active estrogen levels in the circulation. Dysbiosis and decreased gut microbiota diversity reduce the activity of *β*‐glucuronidase and lead to alterations in systemic estrogen levels that mediate the onset of estrogen‐dependent diseases like cardiovascular diseases and bone metabolism‐related diseases [428, 429, 430]. When *β*‐glucuronidase activity is elevated, the level of estrogen in the peripheral circulation increases. This cascade of events is involved in the development of endometriosis and estrogen‐dependent tumors [431]. In addition, the gut microbiome is involved in estrogen metabolism by synthesizing SCFAs. Butyric acid, one of the SCFAs primarily synthesized by the gut microbiome, can regulate estrogen synthesis in porcine granulosa cells [432]. Collectively, these results indicate an indispensable role for the “gut–estrogen axis” in the mediation of reproductive health and physiological functioning of women. Therefore, the role of gut microbiome dysbiosis in the development of female reproductive disorders received considerable attention.

#### Human gut–progestin axis

Tetrahydrocorticosterone (THP), one of the progestins, has a key role in many physiological processes in women (especially those involving the menstrual cycle and pregnancy) and can be generated through the conversion of the abundant biliary corticoids (tetrahydro deoxycorticosterone, THDOC) of the host through 21‐dehydroxylation facilitated by human gut bacteria like *Gordonibacter pamelaeae* and *Eggerthella lenta*. Additionally, the process of 21‐dehydroxylation of THDOC can be promoted by the hydrogen gas produced by human gut microbes like *E. coli* Nissle 1917 (EcN). THP level was significantly increased in the feces of pregnant women, along with an increased abundance of *G. pamelaeae* and *E. lenta*, compared to those of nonpregnant controls [433]. The above results indicate the essential role of gut microbiota in progestin metabolism, confirming the pivotal effects of the gut–progestin axis on female reproductive functions.

### Gut microbiome and polycystic ovary syndrome (PCOS)

PCOS is one of the most common reproductive endocrine and metabolic disorders, affecting approximately 6%–18% of women of reproductive age worldwide. PCOS is manifested as hyperandrogenism (hirsutism and/or hyperandrogenemia) and ovarian dysfunction (anovulation, oligo‐ovulation, and/or polycystic ovary) after eliminating other specific diagnoses such as hyperprolactinemia and atypical congenital adrenal hyperplasia [434, 435]. PCOS patients often exhibit systemic metabolic syndromes such as insulin resistance, obesity, and systemic inflammatory states, as well as other cardiovascular risk factors, and it is a key cause of ovulatory infertility. PCOS is also a major contributing factor to the development of early‐onset type 2 diabetes mellitus and psychiatric disorders [436]. The pathogenesis of PCOS is complex, and its clinical phenotype is characterized by significant heterogeneity. PCOS is a multigenic, epigenetic, and environmentally influenced disease whose pathogenesis has not yet been fully elucidated [435]. The role of gut microbiome dysbiosis in metabolic and endocrine‐related diseases has led to increasing efforts to explore the pathogenic role of the gut microbiome in PCOS.

Fecal microbiomes of PCOS patients showed a lower microbial diversity and different phylogenetic composition compared to healthy controls [437]. Comparison of the fecal microbiomes of healthy controls and PCOS patients demonstrated a significant reduction in the *β*‐diversities of microbiomes, with a significantly elevated abundance of *Bacteroides vulgatus* in the patients [438]. Meta‐analysis of published studies showed a significant diminution in evenness and phylogenetic abundance of the gut microbiota in PCOS patients compared to healthy controls, while diversity indices remained generally unchanged. Further analysis reflected a difference in the gut microbiota between PCOS patients and healthy controls. A decreased abundance in *Lachnospira* and *Prevotella* with enrichment in *Bacteroides*, *Parabacteroides*, *Lactobacillus*, *Fusobacterium*, and *Escherichia/Shigella* was observed in PCOS patients; these alterations featured a reduction in SCFAs‐producing bacteria. SCFAs produced exerted anti‐inflammation effects favoring the establishment of a pro‐inflammatory state in PCOS patients [439].

To further validate the link between gut microbiota alterations and PCOS, some researchers induced a PCOS rat model with letrozole and found a lower abundance of *Lactobacillus*, *Ruminococcus*, and *Clostridium*, with an elevated abundance of *Prevotella*. Furthermore, after transplantation of feces from healthy rats to rats with induced PCOS, the estrous cycle of the latter returned to normal and showed hallmarks of ovarian functions, including a significant improvement in ovarian morphology and attenuated androgen biosynthesis [440]. In addition, transplantation of *Lactobacillus* alone significantly improved the estrous cycle of rats, suggesting that a single genus may be important in PCOS pathogenesis [440]. Fecal microbiota transplantation from women with PCOS or *B. vulgatus* transfer into recipient mice led to the development of PCOS clinical phenotypes such as insulin resistance, altered bile acid metabolism, decreased IL‐22 secretion, and infertility [438], and these changes were chiefly mediated by agmatine, a metabolite derived from *B. vulgatus*. Agmatine activates intestinal epithelial FXR signaling and inhibits the secretion of glucagon‐like peptide‐1 (GLP‐1) in a non‐bile acid‐dependent manner [441]. These studies revealed the critical action of the “gut–ovary axis” in PCOS pathogenesis and emphasized the importance of the gut microbiome, especially a single genus, in PCOS development. Therefore, interventions targeting gut microbiome dysbiosis may comprise a novel therapeutic target in improving symptomology and blocking PCOS progression.

### Gut microbiome and premature ovarian insufficiency (POI)

Ovarian aging is often identified as the gradual decline in ovarian functions with age, characterized by a decrease in the number and quality of oocytes, accompanied by irregular menstruation, infertility, and ultimate cessation of menstruation [442]. Menopause is a sign of natural ovarian aging and occurs mostly between the ages of 49 and 52. In contrast to natural menopause, some women exhibit a significant decline in ovarian functions before the age of 40, referred to as premature ovarian insufficiency (POI). The principal clinical features of POI are abnormal menstruation (amenorrhea or scanty/frequent menses), elevated levels of gonadotropins (follicle‐stimulating hormone [FSH] > 25 U/L), and decreased levels of estrogen before 40 years of age [443, 444]. The global prevalence of POI is approximately 3.7%, with a prevalence of 0.1% in women under 30 years of age [445]. This has drawn increasing concern because of the severe fertility impairment and significant rise in the risk of CVD, cognitive impairment, osteoporosis, and reduced life expectancy in POI patients [444]. POI is also a highly heterogeneous disease with a complicated pathogenesis. A growing number of studies have found that the gut microbiota–ovarian axis contributes to ovarian functions, and gut microbiota dysbiosis may play a key role in the initiation and progression of POI.

The abundance of SCFA‐producing *Blautia*, *Clostridium*, *Faecalibacterium*, *Roseburia*, and *Ruminococcus* fell significantly in the women with POI compared to healthy controls [446]. SCFAs exert anti‐inflammatory and immunomodulatory functions by regulating the immune cells [447], and a reduction in SCFA‐producing bacteria may affect POI progression by influencing the synthesis of SCFAs [448]. Hormone‐replacement therapy (HRT) is a common and helpful clinical treatment for POI patients. HRT treatment reversed the *β*‐diversity, and the abundance of *Eggerthella* in POI patients indicates the critical contribution of sex hormones to gut microbiota composition in POI patients [448]. These results suggest that gut microbiome dysbiosis and microbial metabolites may be central to POI; however, the precise functions and underlying mechanisms of action still necessitate further investigation.

### Gut microbes and endometriosis

Endometriosis affects approximately 10% of women of reproductive age and is defined as the presence of endometrial glands and stromal tissue outside the uterine cavity. The main clinical features of endometriosis are chronic pelvic pain, dysmenorrhea, and infertility. The pathogenesis of endometriosis is complex and associated with genetic, immunological, and environmental factors; however, the exact etiology of endometriosis has yet to be fully clarified [449, 450]. Since the gut microbiota functions as a pivotal contributor to estrogen metabolism, inflammation, and immune functions, the role of gut microbiota in the onset and progression of endometriosis has attracted recent attention. Earlier, an abundance of *Shigella*/*Escherichia* was found to be significantly higher in stage III or IV endometriosis patients compared to healthy controls [451]; however, other authors discerned no difference between the gut microbiota of endometriosis patients and healthy controls during the proliferative and secretory phases of the menstrual cycle [452]. The *α*‐ and *β*‐diversity were significantly lower in fecal samples from endometriosis patients compared to healthy controls [453]. The abundance of two genera of bacteria from class Bacteroidia (*Bacteroides* and *Parabacteroides*) and two from Clostridia (*Oscillospira* and *Coprococcus*) were higher in endometriosis patients, while the abundance of two bacterial genera from the Bacteroidia (*Paraprevotella* and one unidentified) and Clostridia (*Lachnospira* and one unidentified) decreased in stool samples of endometriosis patients. However, larger studies are needed to verify the exact differences in the gut microbiota between endometriosis patients and healthy controls [453].

Besides, stool samples from endometriosis‐induced mice showed reduced diversity and abundance of gut microbiota relative to controls. Furthermore, an elevated Firmicutes‐to‐Bacteroidetes ratio in stool samples from mice with endometriosis was also reported [454, 455]. Dysregulation of the gut microbiome promoted the growth of lesions in endometriosis‐induced mice, and SCFA levels (especially butyrate) were significantly reduced in the stools, while butyrate supply has significantly reduced the size of endometriotic foci [456, 457]. These results suggest that gut microbiota imbalance and microbial‐derived SCFAs have an important role in endometriosis development. However, a definitive cause‐and‐effect relationship between the gut microbiota and the onset and progression of endometriosis in humans remains unclear, and the specific role of intestinal metabolite imbalance in the development of endometriosis requires further analysis.

### Pregnancy and gut microbiota disorders

Pregnancy is a crucial reproductive stage in a woman's life that is accompanied by significant physiological changes in hormone levels, immune status, and metabolism, supporting fetal development [458, 459]. Pregnancy‐related complications, such as gestational diabetes mellitus (GDM) and pre‐eclampsia (PE), are serious risks to the pre‐ and long‐term health of both mother and fetus [460, 461]. The gut microbiota (bacteria and fungi) undergoes significant alterations during the pregnancy period and is primarily influenced by modulations in physiological processes in pregnant women, particularly changes in hormones [462, 463, 464]. Altered gut microbiota contributes to the regulation of endocrine, immune response, and metabolic activities in pregnant women for a successful pregnancy [465]. Gut microbiota dysregulation in pregnant women mediates the onset and progression of several pregnancy‐related complications, such as GDM and PE. GDM is often defined as abnormal glucose tolerance diagnosed or recognized during pregnancy, and it does not meet the diagnostic criteria for overt diabetes mellitus. Although GDM is regarded as a transient hyperglycemia during pregnancy, it is strongly associated with perinatal and long‐term health risks for both mother and fetus [466]. Growing evidence has identified significant disturbances in the gut microbiota of women with GDM. Although there is still variation in the results from various studies regarding gut microbiota dysbiosis of GDM patients, there is a general increase in *Enterobacteriaceae*, *Desulfovibrio spp*., *Aspergillus farinaceus*, Ruminococcaceae, *Prevotella spp*., and *Collinsella spp*., with a decrease in *Alistipes*, *E. faecalis*, and *Bifidobacterium spp*. in GDM patients [467, 468]. In addition, gut microbiota dysbiosis precipitates in the development of GDM mainly through a reduction in microbial‐derived SCFAs [469]. PE is a pregnancy‐specific disorder defined as new‐onset hypertension after 20 weeks of gestation with at least one concomitant complication, such as proteinuria or maternal organ dysfunction, a serious complication jeopardizing the health of the mother and fetus [470]. Numerous studies have confirmed disturbances in the gut microbiome of PE patients relative to healthy pregnant women. Our group noted a significant drop in *α*‐diversity in the gut microbiota of PE patients that was accompanied by a significant elevation in pathogenic taxa such as *Clostridium difficile* (also some beneficial taxa except for *Clostridium butyricum*), *Dialister*, *Veillonella*, and *Fusobacterium* and a significant diminution in probiotic genera such as *Lachnospira*, *Akkermansia*, and *Faecalibacterium*. These microbial changes were strongly correlated with clinical markers of PE, such as blood pressure and biomarkers of liver and renal functions. The gut microbiota of PE patients induced pre‐eclampsia‐like symptoms in pregnant rats, associated with an impaired intestinal barrier and a dysregulation of Th17/Treg ratio, resulting in augmented systemic inflammation and impaired placentation [471]. The gut microbiota of PE patients had a specific loss of SCFA‐producing bacteria, causing a drop in localized propionic acid and butyric acid in the placenta, whereas the PE‐like symptoms were reversed by supplementation with *A. muciniphila*, propionic acid, or butyric acid in mice by promoting placental macrophage autophagy and prohibiting M1 phenotypic transition [472]. In addition, *A. muciniphila* supplementation has significantly ameliorated the clinical phenotype of pre‐eclamptic mice through secreted OMVs, suggesting OMVs as key contributors to the initiation and progression of PE by interacting with placental target cells [473]. Dysbiosis of paternal gut microbiota increased the probability of offspring having low birth weight, severe growth restriction, and premature mortality by interfering with testicular metabolism; this affected the composition of small RNAs in spermatozoa, ultimately resulting in the impairment of placental development [474]. The importance of the gut microbiota on successful pregnancy was emphasized, and the importance of paternal gut microbiota dysbiosis on placental function and outcome of pregnancy was further highlighted; these indices are sensitive to environmental factors, which require additional investigations.

### Section summary

The gut microbiota, a dynamic ecosystem, displays an important function in women's reproductive health and is recognized as a metabolically active “organ” in different phases of female reproductive activities. Dysbiosis in the gut microbiota contributes to the onset and progression of female reproductive diseases through direct interactions with the host or via metabolites/OMVs released from the gut microbiota. A regulatory “gut–germline axis” in males acts as the key interface between the paternal preconceptive environment and intergenerational health in mice, highlighting the importance of environmental factors on reproductive health through the gut microbiota. In addition, specific roles and underlying molecular mechanism(s) of action for certain bacteria with significantly altered abundance within the gut microbiota in patients with reproductively related diseases (such as PCOS) have been identified and are receiving increasing attention. Gut microbiota‐derived metabolites or OMVs act as important indicators for the diagnosis, early prediction, and prognostic assessment of female reproduction‐related diseases. Dietary intervention or probiotic supplementation may improve reproductive disorders by modulating the gut microbiota. Thus, the use of probiotics or fecal transplants to improve the dysbiosis of the gut microbiota may constitute a novel option for the prevention or treatment of reproductive diseases.

The alterations and functions of the gut microbiota in reproductive disorders remain contentious, as various studies present conflicting findings. This inconsistency may be attributed to variations in sample size, study methodologies, ethnicity, geographical factors, and dietary habits. Given the complexities inherent in the pathogenesis of reproductive diseases, the exact roles and mechanisms through which dysbiosis‐associated metabolites and OMVs of the gut microbiota influence disease progression are not yet fully understood. To effectively control potential confounding factors and reveal the definitive pathogenic effects of specific microbe–microbe and microbe–host interactions in reproductive diseases, it is necessary to optimize bioinformatics techniques such as metabolomics, transcriptomics, and single‐cell sequencing, as well as conduct large‐scale randomized clinical trials. Additionally, further research is needed to determine the role of specific bacterial species and their metabolites/OMVs in the onset and progression of reproductive diseases. Furthermore, the controversy surrounding the existence of a placental microbiome and its regulatory effects on pregnancy needs to be addressed. Improved quality and reproducibility of the research approach are required to avoid contamination and be consistent with DNA extraction, both in terms of timing and methods. Larger birth cohorts with normal birth mothers should be established to enable the identification of the existence of the placental microbiome and to track the bacteria that translocate to the gut of infants. Such analyses will further elucidate the relationship between the gut microbiota and reproductive disorders, thus identifying their potential therapeutic value.

## GUT–ENDOCRINE AXIS

### Overview

The gut–endocrine axis involves complex interactions among the gut microbiota, enteroendocrine cells, and endocrine organs, playing an indispensable role in human health and disease. The gut microbiota produces a variety of metabolic products, such as SCFAs, bile acid metabolites, and indole derivatives, which influence systemic endocrine organs through the bloodstream. Enteroendocrine cells, part of the intestinal epithelium, are distributed throughout the digestive tract. They secrete various hormones, such as GLP‐1, peptide YY (PYY), and gastric inhibitory peptide (GIP), which regulate appetite, insulin secretion, and gastrointestinal motility, and their functions are influenced by the gut microbiota [475, 476]. The vagus nerve connects the gut and brain, transmitting information about the gut state to the central nervous system, thereby affecting the functions of the endocrine system [477]. The enteric nervous system, known as the “second brain,” regulates the gut functions independently of the central nervous system and communicates with it through complex neural networks [478]. Hormones and metabolites secreted by the gut microbiota and enteroendocrine cells affect the hypothalamic–pituitary–adrenal (HPA) axis, regulating stress responses and cortisol secretion [479]. The gut microbiota, through metabolites and neurotransmitters, may also influence the hypothalamic–pituitary–thyroid (HPT) axis, regulating thyroid hormone secretion [480]. Additionally, hormones and metabolites secreted by the gut microbiota and enteroendocrine cells affect the hypothalamic–pituitary–gonadal (HPG) axis, regulating sex hormone secretion [481]. Thus, the gut–endocrine axis is a complex biological network involving interactions among the gut microbiota, enteroendocrine cells, the nervous system, and endocrine organs, significantly impacting the development of various endocrine diseases. The following section will specifically elucidate the impact of the gut microbiota on the development of diabetes.

### Diabetes

Numerous studies have demonstrated significant differences in the gut microbiota composition between type 2 diabetes (T2D) patients and healthy individuals [482, 483, 484]. Compared to healthy individuals, T2D patients exhibit a reduced abundance of butyrate‐producing bacteria such as *Roseburia intestinalis*, *F. prausnitzii*, and *Eubacterium rectale*, while there is an increased abundance of opportunistic pathogens like *Bacteroides caccae*, *Clostridiales*, *E. coli*, and sulfate‐reducing bacteria such as *Desulfovibrio* [482, 485]. The reduction in butyrate‐producing bacteria is also a characteristic feature of the gut microbiota in prediabetic individuals. Functionally, the gut microbiome of T2D patients shows an increase in genes associated with sugar transport, BCAA transport, sulfate reduction, and oxidative stress response, along with a decrease in genes related to butyrate biosynthesis [482, 485]. The metabolic profile of the gut microbiota in T2D patients also differs from that of healthy individuals, including reduced levels of SCFAs, altered bile acid composition and concentration, and elevated BCAA levels [486, 487]. Additionally, the concentrations of gut microbiota‐derived metabolites such as imidazole propionate, phenolic compounds like 3‐phenylpropionic acid, and 3‐indole‐lactic acid are higher in T2D patients compared to healthy individuals [487, 488].

The primary mechanisms by which the gut microbiota influences the development of T2D include their effects on energy absorption and balance. The gut microbiota regulates the secretion of host appetite hormones, influencing energy intake. SCFAs, the main end products of gut microbiota fermentation of dietary fiber, act on GPR41/43 receptors on intestinal epithelial cells, promoting the secretion of GLP‐1 and PYY [489]. These hormones suppress appetite and increase satiety [490]. Butyrate reduces the activity of appetite‐promoting neurons in the hypothalamus, decreasing food intake [491]. Secondary bile acids and certain proteins secreted by gut bacteria, such as *E. coli* secreted ClpB and *A. muciniphila* secreted P9 protein, can also stimulate GLP‐1 secretion [492, 493, 494]. Conversely, some metabolites, such as deoxycholic acid and high concentrations of acetate, inhibit GLP‐1 secretion or promote ghrelin secretion, leading to an increased appetite [495, 496]. Indole, a tryptophan metabolite, promotes GLP‐1 secretion in the short term but subsequently inhibits its secretion [497]. The gut microbiota also regulates the thermogenic activity of host adipose tissue and liver, promoting energy expenditure. Oral administration of butyrate in mice promotes the expression of thermogenesis‐related genes, PPAR‐γ coactivator 1*α* (PGC‐1*α*), and uncoupling protein 1 (UCP1) in brown adipose tissue, enhancing energy expenditure and fat oxidation [491, 498]. Acetate upregulates the expression of genes related to fatty acid oxidation and thermogenesis in the liver, inhibiting fat accumulation in adipose tissue and the liver [499]. Succinate and P9 protein can also promote UCP‐dependent thermogenesis in brown adipose tissue [494, 500]. Lithocholic acid binds to the TGR5 receptor in adipose tissue, promoting the browning of white and brown fat to stimulate thermogenesis [501]. On the other hand, this process regulates glycogen synthesis and breakdown, thereby influencing host energy metabolism. The activation of intestinal gluconeogenesis leads to a reduction in hepatic glucose production associated with improved glucose homeostasis. Butyrate, propionate, and succinate activate the expression of genes related to intestinal gluconeogenesis [502, 503]. In contrast, hydrogen sulfide stimulates gluconeogenesis and glycogenolysis in rodent hepatocytes, reducing glucose utilization, decreasing glycogen storage, and disrupting glucose homeostasis [504].

Moreover, the gut microbiota and its metabolites influence insulin secretion and insulin sensitivity. SCFAs regulate glucose metabolism by affecting the functions and secretion of insulin by pancreatic *β*‐cells. SCFAs promote GLP‐1 secretion, which, upon binding to its receptors on the surface of pancreatic *β*‐cells, enhances insulin synthesis and secretion as well as the growth and proliferation of *β*‐cells [505]. On the other hand, SCFAs directly act on GPR41/43 receptors on the surface of *β*‐cells to enhance glucose‐stimulated insulin secretion and improve *β*‐cell functions [506, 507, 508]. Propionate primarily ensures *β*‐cell quality and glucose‐stimulated insulin secretion by inhibiting *β*‐cell apoptosis [506, 507, 508]. However, propionate may also have adverse effects on host glucose metabolism, such as increasing postprandial plasma glucagon, fatty acid‐binding protein, and norepinephrine levels, which can lead to insulin resistance and compensatory hyperinsulinemia [509], thus increasing the risk of T2D [510]. Various products of gut microbiota‐mediated metabolism of proteins and amino acids significantly impact insulin sensitivity and *β*‐cell function. For example, elevated plasma levels of BCAAs are associated with insulin resistance and an increased risk of T2D. In the gut microbiomes of insulin‐resistant individuals, the capability for BCAA synthesis driven by *Prevotella copri* and *Bacteroides vulgatus* is enhanced, while the capacity for BCAA uptake and degradation driven by *Butyrivibrio crossotus* and *Eubacterium siraeum* is diminished [487]. The gut microbiota‐derived metabolite imidazole propionate, a product of histidine metabolism, impairs glucose tolerance and disrupts insulin signaling by activating the mTORC1 signaling pathway [511]. Conversely, the intermediate product of tryptophan metabolism by gut microbiota, 3‐indole propionic acid, is associated with improved insulin secretion and sensitivity, as well as a reduced risk of T2D [512]. Additionally, 4‐methylphenol, a product of protein fermentation by gut microbiota, stimulates insulin secretion and *β*‐cell proliferation [513].

Gut microbiota has a significant role in the impairment of intestinal barrier function and chronic inflammation, which are crucial in the development and progression of diabetes. Damage to the intestinal barrier allows bacteria and toxins from the intestinal lumen to enter the bloodstream, triggering both local and systemic inflammatory responses and insulin resistance, thereby leading to impaired glucose tolerance [514]. The mucus layer, tight junctions, and intestinal epithelial cells form the structural foundation of the intestinal mechanical barrier, all of which are influenced by the gut microbiota. Gut microbiota and its metabolites also regulate the synthesis and secretion of intestinal mucins. Butyrate promotes the proliferation of goblet cells (mucin‐secreting cells) and the expression of the mucin‐2 gene, increases mucin‐2 secretion, and improves the intestinal mucus barrier [515]. Similarly, the beneficial effects of *A. muciniphila* on the intestinal barrier are related to its ability to promote goblet cell proliferation [516]. Gut microbiota also regulates the expression of tight junction protein genes in the intestinal epithelial cells. Butyrate and indole enhance intestinal barrier function by upregulating the expression of tight junction protein genes in the intestinal epithelial cells [517, 518]. The outer membrane protein Amuc_1100 of *A. muciniphila* acts on TLR2, regulating the expression of tight junction protein‐related genes in the intestinal epithelial cells and improving the intestinal barrier [519]. However, gut microbiota dysbiosis leads to an increase in metabolites that are detrimental to the intestinal barrier function, such as ethanolamine and trans‐fatty acids, which downregulate the expression of tight junction protein‐related genes and disrupt the intestinal barrier [520, 521]. Gut microbiota also regulates the number of intestinal epithelial cells. *A. muciniphila* and its membrane protein Amuc_1100 promote the regeneration and repair of intestinal epithelial cells, thereby maintaining the integrity of the intestinal barrier [522]. Dysbiosis and impaired intestinal barrier function may lead to the translocation of bacteria or their metabolites into the host circulatory system, which is a crucial mechanism for inducing low‐grade chronic inflammation in the host. Lipopolysaccharide (LPS), a component of the cell wall of Gram‐negative bacteria, activates the CD14/TLR4 signaling complex on the surface of innate immune cells once it enters the bloodstream, inducing the secretion of pro‐inflammatory cytokines and promoting inflammatory responses [523]. Conversely, butyrate promotes the differentiation of anti‐inflammatory Treg cells, suppressing inflammatory responses in the gut and peripheral tissues, and it can directly act on histone deacetylases to alleviate intestinal inflammation [524, 525]. The presence of low‐grade chronic inflammation in T2D patients may also be related to the reduction of anti‐inflammatory bacteria such as *F. prausnitzii* in their gut. *F. prausnitzii* mitigates inflammatory responses by blocking NF‐κB activation and IL‐8 production [526].

### Gut microbiota‐targeted interventions to alleviate diabetes

Directly Altering the Structure of Gut Microbiota: Transplanting the gut microbiota from lean, healthy individuals to patients with obesity and metabolic syndrome can effectively improve the recipients' insulin sensitivity [527, 528]. Recent studies have also found that FMT has certain therapeutic effects on diabetic peripheral neuropathy [529]. Additionally, supplementing with a mixture of one or more probiotics can enhance insulin sensitivity and reduce insulin resistance in patients with T2D or obesity [530]. Clinical studies have further shown that the combined use of probiotics with prebiotics or medications may have an even better effect on improving glucose metabolism [94].

Indirectly Regulating the Structure of Gut Microbiota: Multiple clinical trials have confirmed that dietary interventions with high dietary fiber can significantly improve glucose and lipid metabolism in patients with obesity and T2D and reduce gut permeability, systemic inflammation, and insulin resistance [483, 531]. This is achieved by selectively enriching beneficial bacteria in the gut, such as SCFA‐producing bacteria, thereby increasing SCFA content in the gut and promoting the secretion of gut hormones like GLP‐1 and PYY. Similarly, the Ma‐Pi 2 diet, which is rich in dietary fiber, effectively improves glucose and lipid metabolism in T2D patients by enriching SCFA‐producing bacteria and inhibiting pro‐inflammatory bacteria in the gut [532].

### Challenges in the field

The progression from a healthy state to prediabetes, the onset of type 2 diabetes, and the development of various complications is a complex multiorgan disease involving metabolic, immune, and nervous systems. Due to the limitations of previous research methodologies and strategies on microbiota, studies on the gut microbiota and diabetes are still in their early stages. The gut microbiota is a complex ecological community, where its members are far from being disorganized or isolated; instead, they function through a network system formed by ecological relationships such as cooperation and competition. More importantly, the functions of individual members within the gut microbiota are not equivalent. Key members influence the host's health and disease states through interactions with the host, and they can determine the stability of the community by regulating other members within the ecological network. These key members constitute the core microbiota of the gut microbiome. Despite significant efforts, characterizing the core microbiota and understanding their interaction mechanisms with the host remain critical scientific challenges in this field.

In earlier studies, the relationship between microbiota and disease was predominantly correlational, but current research is advancing toward causal and mechanistic investigations. The gut microbiota and their metabolites are recognized and sensed by the gut, participating in the regulation of the intestinal epithelial barrier and altering innate and adaptive immune signals/cell functions, potentially affecting systemic inflammation mediated by distant organs. The gut microbiota can regulate gut–brain peptides such as cholecystokinin, ghrelin, PYY, and GLP‐1, which may subsequently impact neuronal function in the brain and the gut–brain axis biofeedback system, thereby influencing energy homeostasis. Thus, the gut microbiota influences the onset and progression of diabetes through interactions with the host's immune and nervous systems. However, the immune system effector molecules, signaling pathways involved in these microbiota interactions, and the functional neurons of the nervous system remain unclear. Given the complexity of diabetes as a multiorgan, multistage disease, elucidating the multidimensional and multilayered molecular mechanisms of key microbiota–host interactions remains a significant challenge.

### Section summary

As one of the most severe chronic diseases in contemporary society, developing more cost‐effective prevention and treatment strategies for T2D is an urgent issue. The gut microbiota, as a potential therapeutic target, offers new hope for the prevention and treatment of T2D. Regulating the gut microbiota through high‐fiber diets and other methods to alleviate T2D has broad application prospects, but it is still in its early stages and faces numerous challenges before clinical application. One challenge is the contradictory findings regarding the association between T2D and specific bacteria in different studies. For example, some studies found a decreased abundance of *Bacteroides intestinalis* in the gut of T2D patients, while other studies have shown an increased abundance [482, 485]. Another challenge is the individual variability in the gut microbiota, which can affect the efficacy of interventions. For instance, the ratio of *Prevotella* to *Bacteroides* in the gut influences the effectiveness of barley kernel bread in improving an individual's blood glucose and insulin secretion [533]. Additionally, differences in the gut microbiota composition among populations impact the effectiveness of fecal microbiota transplantation in improving the insulin sensitivity of patients with metabolic syndrome [528]. Finally, prebiotic interventions do not always improve glucose metabolism and may even have adverse effects [534, 535]. These issues limit the clinical development and application of gut microbiota‐targeted interventions for alleviating T2D. In conclusion, more research is needed to elucidate the mechanisms by which gut bacteria regulate the development and progression of T2D. Additionally, more clinical studies are necessary to achieve precise regulation of the gut microbiota, thereby enabling more effective alleviation and treatment of T2D.

## GUT–BRAIN AXIS

### Overview

The gut microbiota can generate various metabolites that interact with the host through neural, immune, and metabolic pathways, influencing brain function and maintaining systemic homeostasis. This bidirectional communication is called the microbiota–gut–brain axis [536, 537], representing a tightly interconnected and complex network that regulates metabolism, immune homeostasis, and central nervous system functions [538, 539, 540, 541]. Numerous reviews have systematically summarized the research progress of the gut–brain axis in depression, Parkinson's disease, and Alzheimer's disease. Our review will primarily focus on its research advances in neurodevelopmental disorders caused by abnormalities in maternal–infant microbiota transmission.

As a significant physiological process, maternal–infant vertical transmission indicates that maternal microbiota may interfere with gut–brain signaling and thus play a crucial role in offspring's neurodevelopment in numerous unexpected and fascinating ways [542]. Deletion and selective reconstitution of maternal gut microbiota can affect offspring's neurodevelopment. Both germ‐free and antibiotic‐treated maternal embryonic brains show reduced expression of genes associated with axonogenesis, insufficient numbers of thalamocortical axons and impaired thalamic axon growth in mice. In contrast, maternal mice colonized with *Clostridia*‐dominant spore‐forming (Sp) bacteria can elevate the levels of trimethylamine oxide and imidazole propionic acid in the fetal brain preventing defects in fetal thalamocortical axonogenesis [543], suggesting that a structurally and functionally normal maternal microbiome is essential for the fetal neurodevelopment.

**Figure 11:**
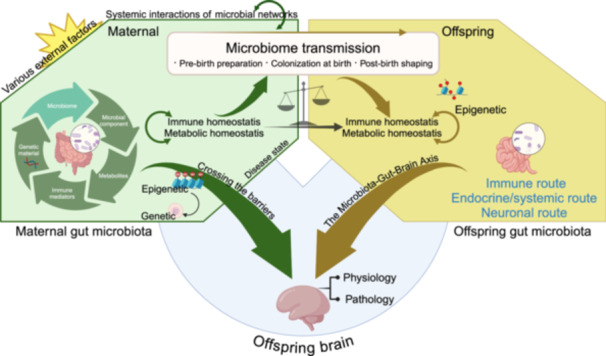
The interaction pathway between the gut and brain in maternal and offspring host systems. The conceptual framework delineates an overview of the multifactorial influences exerted by the maternal gut microbiota and its derived components on a range of physiological processes, including metabolism, immunological functions, embryonic development, and lactation. The maternal microbiome and its derived metabolites intricately modulate offspring neurodevelopment during both prenatal and postnatal periods. These effects may occur independently of, or in conjunction with, mother–infant microbial transmission processes, ultimately shaping the gut microbiota of the offspring. Created with BioRender.com.

A growing body of evidence indicates that a myriad of intricate maternal and postnatal factors can induce structural and functional abnormalities in the developing brain of offspring, with repercussions that extend into adulthood [544, 545]. Some of these effects may even be transmitted longitudinally across germ lines through intergenerational inheritance [546]. Prospective studies have linked maternal infections during pregnancy to an elevated risk of mental disorders in the offspring [547]. High psychological stress during pregnancy is associated with an increased risk of behavioral problems and mental disorders in children [548]. Additionally, maternal metabolic status, dietary habits, and behavior during breastfeeding influence the neurodevelopment of offspring, potentially altering their susceptibility to conditions such as anxiety, depression, and cognitive impairments [549, 550, 551]. Despite these associations, the underlying mechanisms remain largely elusive. Herein, we critically examine the current reports concerning the impact of maternal gut microbiota on offspring neurodevelopment. We aim to provide a comprehensive framework for examining the mechanisms of mother–infant microbiome interactions and their influence on neurodevelopment, thereby offering a multifaceted and progressive perspective in this rapidly advancing field (Figure 11).

### Shifting periods of microbial growth and brain development

Early life represents the most dynamic period for both microbiota maturation and brain development, with microbial colonization and proliferation occurring concurrently with neurodevelopment, both of which take place within a critical window of heightened sensitivity to external influences [552]. During these critical periods, rapid changes in neuronal organization, including, but not restricted to, neurogenesis, axonal and dendritic growth, the refinement of prominent connections, and myelin sheath production, are observed. These processes form the body's functional neural circuits essential for normal cognitive, motor, and emotional development. Simultaneously, the intestinal microbiota undergoes a gradual transition from an unstable state characterized by a loose microecological structure and low maturity to a more established composition featuring complex, three‐dimensional functional pathways [553]. Given the extensive research on the critical role of microbiota–gut–brain axis communication in structural and functional changes, it becomes apparent that altering or interrupting the initial colonization or development of the gut microbiota during this period is highly likely to profoundly affect gut–brain signaling. Therefore, an altered microbiome may raise the risk of neurodevelopmental issues and affect lifelong health. All these theories highlight the importance of the microbiota in early development [554, 555]. Supporting this, studies with germ‐free mice have shown the microbiome's significant role in early neurodevelopment [554, 556, 557].

### Systemic interactions of maternal gut microbiota play an indispensable role in the development of the offspring's gut microbiota

**Figure 12:**
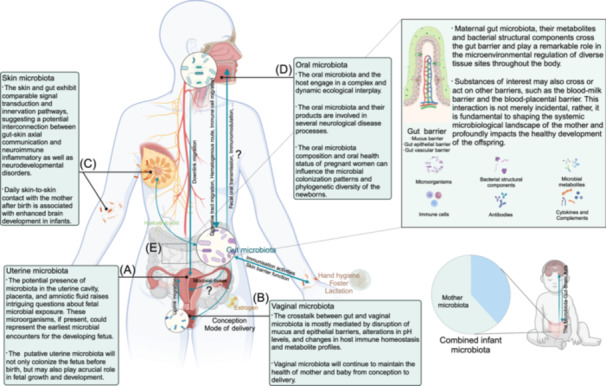
Conceptual framework: Mechanisms through which systemic interactions of maternal gut microbiota influence offspring's neurodevelopment. The human microbiome exhibits a remarkable degree of interconnectedness across various ecological niches, including the gut, oral cavity, vagina, uterine cavity, and skin. These diverse microbial communities often display overlapping structural compositions and functional attributes. This intricate network of maternal microorganisms engages in complex interactions and synergistic activities throughout physiological and pathological processes, potentially exerting profound influences on the neurodevelopmental trajectories of offspring. Moreover, these distinct ecological niches within the maternal body play crucial roles in facilitating the vertical transmission of microbiota from mother to child. This process of microbial transfer is not confined to a single site but rather involves a coordinated effort across multiple maternal habitats, such as Uterine microbiota (A), Vaginal microbiota (B), Skin microbiota (C), Oral microbiota (D), and the major barriers of the body (E), especially the intestinal barrier, are an important link in the systemic interactions of microorganisms. Created with BioRender.com.

Numerous factors can influence the colonization and composition of the early fetal microbiota, including genetic factors, the surrounding environment, maternal diet during pregnancy, exposure to infections, and mode of delivery. Notably, microorganisms from multiple ecological sites in the human gut, oral cavity, vagina, uterine cavity, and skin may have some overlap in structural compositions and functional attributes [558, 559, 560, 561, 562]. Staged development of barrier functions early in life may provide opportunities for bacterial translocation and communication. For example, the oral microbiota has the potential to impact the composition of the gut microbiota via mechanisms such as intestinal migration, hematogenous pathways, and the migration of immune cells [563] (Figure 12). Research on atopic dermatitis suggests that the complement system, neuroendocrine regulation, and AHR‐mediated immune signaling may influence the gut–skin axis, impacting disease progression and host balance [384, 564] (Figure 12). Sequencing studies show that early infant fecal microbes come from various maternal sources [565]. The collective contribution of these diverse microbial ecosystems may underscore the holistic nature of mother‐to‐infant microbiome transmission during the critical window period and its potential long‐term implications for offspring's health and development (Figure 12).

### Prenatal transmission and priming: The role of maternal gut microbiota in shaping offspring's intestinal microenvironment and physiology

**Figure 13:**
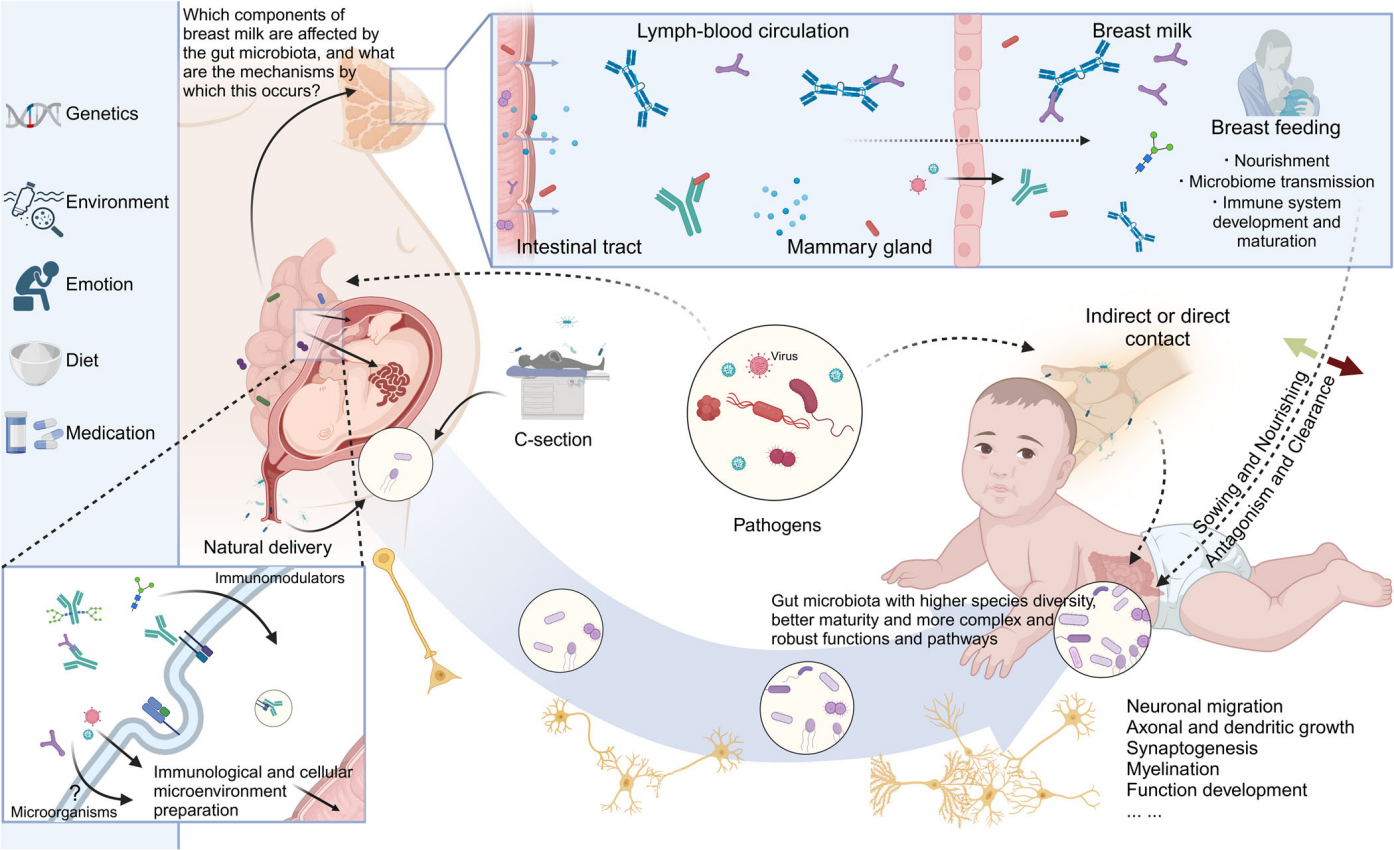
Maternal gut microbiota regulates offspring's neurodevelopment by influencing gut microbial colonization. The maternal gut microbiota exerts a profound influence on offspring's microbial colonization through dual pathways. Primarily, it directly shapes fetal microbiota composition during birth. Additionally, its impact on fetal microbiota colonization may commence as early as the maternal–fetal interface, persisting postnatally through breastfeeding, nurturing, and maternal companionship. The intricate dialogue between maternal gut microbiota and their metabolites at the maternal–fetal interface potentially primes offspring for adaptive responses and microenvironmental preparedness. Postnatal mother–infant contact and mode of delivery determine the microorganisms to which infants are first exposed, setting the tone for the development of gut microbiome colonization in the offspring. Breast milk composition is intricately influenced by maternal gut microbiota, which serves as a continuous inoculum for the offspring's gut microbiome. During breastfeeding and nurturing, the offspring's gut microbiota undergoes maturation and stabilization, concurrently contributing to the refinement of neurological functions. Throughout both prenatal and postnatal stages, a complex interplay of environmental factors, dietary influences, and pathogen exposures modulates the process of maternal gut microbiota transmission to offspring. This transfer plays a substantial role in the offspring's neurodevelopment. Created with BioRender.com.

**Figure 14:**
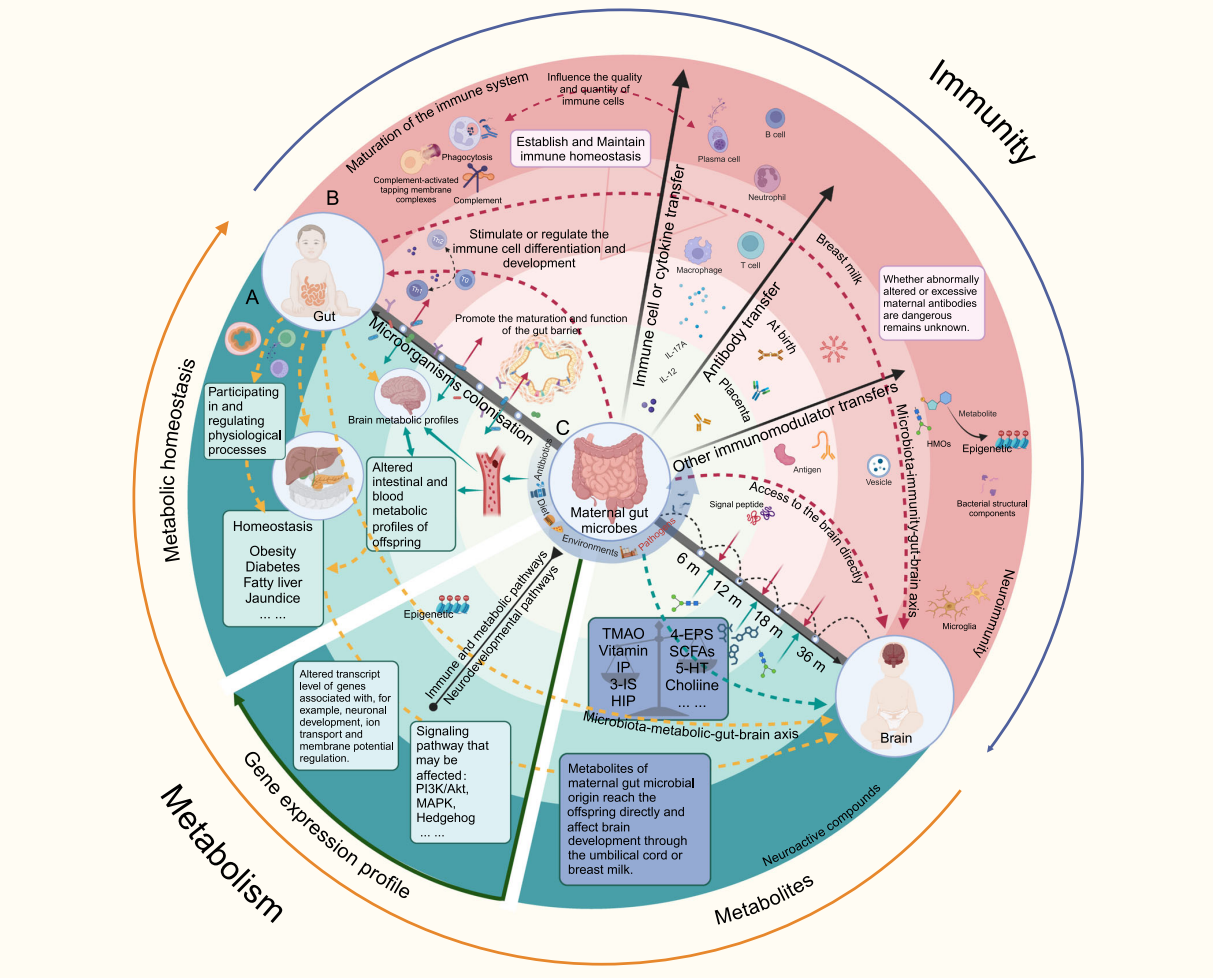
Maternal gut microbiota affects the neurodevelopment of the offspring through the microbiota–metabolic/immunity*–*gut*–*brain axis. (A) The maternal gut microbiota and its metabolites exert a tripartite influence on offspring's metabolic homeostasis, facilitating both direct and indirect communication with the developing brain. (B) Mother‐to‐child immune transfer has profound implications in regulating the structure of the gut microbiota of the offspring, guiding immune maturation, and modulating neuroimmune health. (C) The median axis represents the continuous developmental trajectory of the offspring's gut and brain. This process is subject to persistent and strong interactions between metabolic and immune factors. The intricate interplay of these systems emphasizes the dynamic nature of early development and the long‐lasting impact of maternal influences on offspring. Created with BioRender.com.

Currently, the discussion on the presence of microbes in the prenatal embryonic gut remains controversial due to technical limitations [566, 567, 568], and it is unclear whether maternal gut microbiota can cross the placental barrier to seed the offspring's gut directly (Figure 13). Detection of microbiota in the placenta and uterine cavity of pregnant women challenges the notion of a sterile fetal environment [569, 570, 571] (Figure 12A). Some investigators have detected common oral commensal and periodontal pathogens in the placenta and amniotic fluid [572, 573]. The microecological environment of women's upper and lower reproductive tracts shows a continuous microbial distribution, with the uterine cavity containing a mixture of microbiota from the vagina and fallopian tubes. In this environment, *Pseudomonas aeruginosa* and *Serratia marcescens* are the dominant organisms [574], suggesting an upward migration of vaginal microorganisms to the uterine cavity (Figure 12B). Concurrently, the high degree of bacterial homology observed at the mucosal tissue site implies the possibility of intestinal microbes invading the endometrium by a unique mechanism when the intestinal mucosal epithelial barrier is immature or disrupted [559]. These findings demonstrate a correlation between uterine, oral, vaginal, and intestinal microorganisms, indicating a close interaction between neonates and maternal gut microbiota before birth. This implies the influence of maternal microbiota on infant health within the uterus. Despite the existing uncertainties, it is evident that the maternal microbiome's impact on the microbial colonization of the offspring may commence prenatally through various mechanisms. It is well established that the fetus undergoes one of the most significant environmental transitions as it moves from the protected uterine environment to the external world. The maternal microbiota is well positioned to support the newborn for the long term in meeting this challenge during this crucial life stage [575]. The mother–infant dichotomy maintains a special and intimate connection through the umbilical cord and breast milk [576], serving as a bridge for maternal microorganisms to influence the offspring's development. In utero, microbe‐affected maternal immune cells, IgG, small amounts of IgE, microbial antigenic structures, and other immunomodulators are transferred via the placenta, where they exert effector functions such as neutralization, phagocytosis, and pro‐immune cell activation in the fetus to prepare a suitable immune and cellular microenvironment for fetal microbial colonization at and after birth (Figure 13) and to have an effect on the development of the immune system and the offspring's health (Figure 14). For instance, a selective placental transfer of maternal IgG provides passive immunological protection for the newborn infant [577]. It has been suggested that maternal HIV infection may reduce the efficiency of placental transfer of pathogen‐specific IgG by affecting the ability of maternal IgG to bind to the placenta‐expressed Fc receptors FcγRIIa and FcγRIIIa, thereby reducing the efficiency of placental transfer of pathogens [577, 578]. Thus, the maternal gut microbiota may regulate the characteristics and quantity of antibodies transferred to the offspring in various ways. In addition to antibody transfer, maternal gut microbes also regulate several immune cells, cytokines, and complement components that influence pregnancy outcome and fetal development. For example, under physiological conditions, maternal gut microbes induce Th17 differentiation and promote the insertion and growth of trophoblast cells to support the healthy development of the offspring [579]. Elimination or alteration of maternal gut microbes during pregnancy recalibrates the distribution and function of immune cells in the offspring, leading to an increased probability of disease [580, 581]. Notably, alterations in the microbial structure induced by maternal exposure to stress, dietary changes, and infections during pregnancy produce excessive amounts of immune products or immunomodulatory metabolites that may directly or indirectly mediate brain damage in the fetus. In these events, the link between maternal viral infections and offspring neurodevelopment has a long history. For example, based on ecological and epidemiological studies of certain viruses (rubella, influenza, measles, mumps, chickenpox, and polio), scientists have essentially mapped their association with the prevalence of neurodevelopmental disorders such as autism and schizophrenia [582, 583]. Follow‐up studies using polyinosinic–polycytidylic acid (poly I:C) to simulate pregnancy‐related viral infections in animals have shown offspring with brain and behavioral traits similar to human neurological diseases [584]. Excessive activation of IL‐17a signaling in the gut following maternal pregnancy infection leads to malformations in fetal cortical development and affects chromatin accessibility of naive CD4+ T cells, leading to fetal neurodevelopmental disorders and severe intestinal inflammation [579, 585, 586]. Additionally, other signals generated by maternal immune activation affect the striatal, hippocampal, and cortical volumes of the offspring, alter the transcriptional levels in brain regions like the dorsal–ventral nucleus of the hippocampus and the anterior cingulate gyrus cortex, and cause deficits in the perineuronal matrix network [587, 588]. Moreover, a maternal high‐fat diet leads to the accumulation of endotoxin in fetal tissues, increases macrophage toll‐like receptor 4 signaling, and induces excessive phagocytosis of serotonin (5‐HT) neurons in the dorsal raphe nucleus (DRN) by microglial cells, which increases offspring susceptibility to neurological diseases [589].

In addition to immune signaling, during the embryonic period, a variety of maternal microbial metabolites are detectable within the fetoplacental unit, with transmission to the fetus occurring through the umbilical cord [543, 567, 590] (Figure 14). Some of these metabolites reach the fetal brain or affect axon formation and neuronal connectivity directly, while others, like SCFAs [591], can reach other components of the sympathetic nervous system, gut, and pancreas. These metabolites may be trapped by free fatty acid receptors GPR41 and GPR43, promoting the development of nerve cells, enteroendocrine cells, and pancreatic *β*‐cells, thus affecting the long‐term energy metabolic balance and neurodevelopment of the offspring [592, 593]. Therefore, the dynamics of the intestinal mycobiome during pregnancy are covariant with metabolites in host serum, and interactions between related fungal genera and the host may collectively mediate adverse pregnancy outcomes such as GDM and fetal overgrowth [463]. Furthermore, maternal high‐fat diet‐mediated changes in the gut microbiota elevate maternal plasma levels of kynurenine and modulate the levels of molecules involved in the glutamate–glutamine cycle in the embryonic brain, impairing offspring's behavior [594]. In addition, maternal infections or changes in microbial structure during pregnancy can lead to the gradual buildup of harmful metabolites, bacterial products, DNA, and virulence factors in the fetal environment. This accumulation can affect the embryo's metabolite levels and the bioavailability of essential nutrients, potentially causing direct damage to the fragile fetal brain. Advances in detection methods at the maternal–fetal interface will enhance the ability to trace metabolites from early in life, boosting the potential of this research field [595] and decoding bacterial metabolic mechanisms. The maternal gut microbiota, their metabolites, and maternal immune transfer work together to prepare the fetal gut microbiota and physiology, speeding up the maturation of the fetal immune system during early microbial colonization, which significantly impacts the offspring's neurodevelopment.

### Gut microbiota colonization at birth

While debate persists regarding the direct colonization of the fetal gut by maternal microorganisms before birth, substantial evidence indicates vertical transmission of maternal microbiota from multiple sites during and after birth to the infant. Frequent interactions and sharing between maternal microbiota and infant gut microbiota occur early in life, with the mother contributing most significantly to the offspring [596]. Different delivery modes alter the nature of initial microbiome exposure to the fetus, resulting in different colonization effects (Figure 13). Infants delivered by cesarean section exhibit a markedly different microbial composition and developmental trajectory, as they are primarily exposed to microorganisms from the hospital environment. On the other hand, infants born vaginally are primarily colonized by the bacteria from the maternal reproductive tract [597]. Notably, gut microbes may be involved in this process by participating in the metabolism of key host factors such as estrogen to regulate the vaginal microenvironment and functions [431, 598, 599]. Cesarean delivery has often been associated with a tendency toward stunted gut microbiota, such as a lower abundance of *Lactobacillus* spp. in their fetal stools and a higher rate of colonization by conditionally pathogenic bacteria, significantly affecting the newborn's health, which potentially extends into adulthood [600, 601]. However, this significant alteration in fetal gut microbiota structure caused by cesarean section can be effectively corrected by maternal fecal transplantation after birth [602]. This further supports the role of maternal gut microbiota in shaping the offspring's gut microbiota during development.

In addition, in the early stages of life, the persistence and ecological adaptation of the maternal gut microbiota in the infant's intestine becomes more pronounced during the subsequent breastfeeding and day‐to‐day nurturing period, and they gradually become an important component of the infant's developing gut microbiota [565].

### Postnatal transmission and shaping of microbiome

After birth, the mother's breastfeeding and nurturing behaviors take over the gut microbe‐dependent mother–fetus interface dialogue, promoting the infant's health and long‐term well‐being. Maternal microbial metabolites (SCFAs and oligosaccharides [603, 604, 605]) and vesicles [606], and gut microbe‐associated immune substances (antigenic peptides, immunoglobulins, cytokines, and complement components) reach the mammary glands through lymphatic‐blood circulation, becoming a part of the breast milk (Figure 13). Therefore, breast milk is rich in nutrients and biologically active factors, which strengthens the infant's GI tract [607, 608] and promotes healthy brain development [609]. Microorganisms partially derived from the gut in breast milk have been increasingly reported at the species, genus, and strain level using culture and sequencing methods [610, 611]. These microorganisms may or may not directly seed the offspring's intestinal tract via the intestinal mammary–oral axis [611, 612]. For example, bifidobacterial communities may be transmitted from mother to child through breast milk [613]. Evidence for the role of phages in the mother–infant link has also been documented. Coevolution between phages and their host bacteria may influence the diversity and maturity of the infant gut microbiota. Bifidophages are involved in influencing the composition and function of *Bifidobacteria* in the infant's gut, possibly via vertical transmission from the mother's gut to the infants [613]. Accordingly, the pioneering colonization of some bacteria enhances or limits the fitness of others by rewriting the metabolomic profiles and immune environment in the offspring's intestine [614]. Metabolites derived from maternal gut microbiota, along with IgA that anchors microorganisms, can be transmitted to the infant through breast milk postpartum. This transmission indirectly promotes the colonization and symbiosis of specific fetal microorganisms, supports the development of beneficial intestinal microbiota, and facilitates immune system maturation, thereby protecting against invasive infections [575, 576, 615]. For example, SCFAs (acetate and propionate) and other fermentation metabolites formed by intestinal microorganisms in breast milk not only promote the colonization of bacteria such as *Bifidobacterium* in the offspring's intestinal tract by serving as a cross‐feeding substrate but also exert antimicrobial activity against *Clostridiaceae* and *Peptostreptococcaceae* organisms [616]. On the other hand, high levels of arachidonic acid (AA) in breast milk can lead to gut microbiota dysbiosis and immune disorders in infants [617]. After undergoing digestion by various proteases in the offspring, the proteins in breast milk release thousands of polypeptides in the intestine, performing diverse functions. A related study has validated the peptides with growth inhibitory activity against *Staphylococcus aureus*, providing strong evidence for breast milk proteins in tailoring the development of offspring's gut microbiota [618].

Simultaneously, the nurturing process, replete with daily contact, is an important part of the seeding of the mother's gut microbiota to her offspring. The mother's skin microbiota has been identified as a source of microorganisms for the infant's skin, mouth, gut, and other parts of the body and is involved in building a mature, symbiotic microbial network between multiple body parts of the mother and infant intimately [619, 620, 621, 622, 623]. A randomized controlled trial in the Netherlands identified significant differences in gut microbiota composition and bacterial abundance and predicted functional enrichment in infants who had daily skin‐to‐skin contact with their mothers compared to controls [565, 619, 624]. Although the study cannot exclude the influence of factors like breastfeeding duration, the results support the idea of microbiota transmission and alterations after birth (Figure 13). Daily skin‐to‐skin contact with the mother is associated with better physical conditions and brain development in infants [624, 625, 626]. At 3 years of age, children who received skin‐to‐skin contact interventions also showed fewer internalizing and externalizing behavioral problems [627]. It follows the idea of a healthy maternal gut microbiota co‐shaping the offspring's gut microbiota profile. When gut dysbiosis is induced in mothers exposed to infections during pregnancy and breastfeeding, dietary changes, and stress, it leads to large amounts of inflammatory cytokines (e.g., IL‐17A) or other secondary substances crossing the placenta or entering the mammary gland to directly reach the offspring. This may alter the bioavailability of substances, the immune microenvironment, and the process of initial microbial colonization of the progeny [628] (Figure 13). Dysbiosis of the maternal gut microbiota induces endogenous mastitis development through endotoxemia, leading to a reduction in host anti‐inflammatory enzyme activity in animals [629]. In addition, maternal infections during pregnancy and the postnatal period are likely to directly create the conditions for vertical transmission of susceptible pathogens (Figure 13), leading to a range of adverse outcomes such as neonatal septicemia and meningitis. It is important to note that many viruses may have the ability to cross the placenta and also enter the breast milk during maternal infection [630, 631, 632]. These vulnerable pathogens exhibit diverse pathogenic characteristics and influence the development of neonatal systems through multiple mechanisms. There is no lack of bacteria that can directly attack the newborn's brain during the “window of opportunity” when the body's major barrier functions and saturation are not matched [633]. The virulence factors and secondary inflammatory mediators produced by these bacteria infiltrate the brain without any regulation, leading to severe neurological conditions such as encephalitis and white matter damage. These pathological processes may result in enduring neurological impairments and sequelae [634, 635]. In essence, the impact of maternal microbiota on offspring's microbiota colonization, immune maturation, and intellectual development is obvious. During this critical period, maternal gut microbiota shapes the offspring's early microbiome and immunity, which together regulate normal neurodevelopment through gut–brain communication [636].

### Maternal gut microbiota influences offspring's neurodevelopment through microbiota–metabolites–gut–brain axis

Microbial metabolites are biologically active substances produced by microorganisms through decomposition or synthesis in the process of material and energy transformation. Several gut microbial metabolites have an important role in host neurological health and physiological processes [637]. SCFAs, for example, transfer from the intestinal mucosa to the circulation, interfere with immune regulation and enteric nervous system functions, regulate the secretion of neuropeptides, and affect brain functions [638, 639]. SCFAs cross the blood–brain barrier to interact with nerve cells directly. For instance, direct activation of calcium/calmodulin‐dependent protein kinase II (CaMKII)‐labeled neurons in the bed nucleus of the stria terminalis (BNST) increases their presynaptic glutamate release and fatty acid *β*‐oxidation levels, which affect social behavior [640]. The microbial metabolite 4‐ethylphenol sulfate can also directly impair the maturation of oligodendrocytes in the paraventricular nucleus of the thalamus, thereby altering activity and functional connectivity in specific areas of the brain and triggering anxiety [641, 642]. Healthy gut microbial metabolites in early life assist the body at appropriate levels in various physiological processes, such as promoting intestinal peristalsis and contraction, participating in cellular signaling transduction, neurotransmitter synthesis, and release, and being responsible for establishing metabolic and immune homeostasis. Therefore, the role of the maternal gut microbiota in neonatal microbial colonization and development, as previously described, significantly influences early‐life microbial metabolism and the establishment of a stable and healthy reciprocal microbial–host symbiotic relationship (Figure 14). Conversely, compromised maternal microbiota under conditions such as dietary changes and exposure to infections may disrupt this delicate synergistic relationship. In brief, the role of maternal gut microbiota in relation to the gut microbiota and metabolic homeostasis of the offspring may contribute to the development of metabolic and neurological disorders (Figure 14). For example, offspring with mothers having impaired metabolic health (e.g., impaired glucose tolerance) also tend to develop metabolic disorders, immunological disturbances, and neurodevelopmental deficits [643]. The neonatal gut microbiota in the maternal group with GDM followed the same trend of changes as the mothers. Compared with the healthy control group, the abundance of glycerophosphorylcholine, glycolic acid, and rhamnose in the fecal metabolites of neonates in the GDM group was significantly lower, while the abundance of riboflavin and taurine was significantly higher. These changes were in line with the trends observed in maternal feces, suggesting that GDM mothers may lead to abnormal microbial distribution and function in their offspring, which will adversely affect the metabolic functions and nutrient absorption of the offspring, posing many potential risks to further growth and development [644]. In mice, maternal high‐fat diets induce intestinal microbiota disorders, neurotransmitter alterations, metabolic function abnormalities, and alterations in the transcription of genes related to brain and neuron development, ion transport, and regulation of membrane potential in the offspring, causing neurodevelopmental deficits [645, 646, 647]. Together, these results demonstrate substantial changes in the composition of the maternal gut microbiota, and its metabolites, when the maternal state is altered. Vertical transmission of them into offspring reprograms infant's gut microbiota and metabolic capacity, affecting the metabolic functions of peripheral cells and organs including brain, thereby altering the neural developmental trajectory [648]. The above research underscores the critical importance of maintaining a healthy maternal microbiome and metabolic state during early life, as it has far‐reaching consequences for the offspring's metabolic health and neurodevelopment.

The mother–infant interactions increase rapidly after childbirth, accompanied by many sensory, perceptual, and behavioral changes. Unsurprisingly, the absence of these sensory perceptions and maternal behaviors affects the physical and psychological development of the offspring as it grows up [649, 650]. In comparison, maternal infection or colonization by specific strains of bacterium triggers a reduction in maternal care (licking, grooming, sniffing, etc.) and nursing of the offspring in mice, which impairs the offspring's access to essential nutrients. Mechanistically, the pups have attenuated serine/threonine kinase Akt signaling and impaired secretion and stability of circulating insulin‐like growth factor 1 (IGF1) in serum, resulting in delayed and impaired development of the offspring's systems [651]. Of course, to some extent, it remains unknown whether dynamic changes in gut microbiota mediate changes in maternal behavior. In addition, the study of metabolites/neurotransmitters that are highly involved in microbiota and behavior, such as serotonin (5‐HT), in the behavior of offspring close to the mother has provided new ideas and perspectives for exploring how microbiome‐derived neuroactive compounds affect offspring's neurodevelopment [652].

On the other hand, maternal gut microbial metabolites during breastfeeding can skip the offspring's phylogeny and directly interact with the brain cells, affecting the development of fetal brain structure and functions. Interestingly, more and more microbial metabolites are used in clinical trials as potential therapeutics for complex diseases [653]. Breast milk is the best natural food for infants, containing almost all the nutrients needed for infant growth and development, including proteins, fats, carbohydrates, vitamins, hormones, and other biologically active substances that are closely related to the development of the immune system and the nervous system [654, 655]. Long‐chain fatty acids, iron, choline, folic acid, and sphingolipids in breast milk are significantly associated with early myelin formation in many brain regions of the offspring, providing the basis for the brain links that will support the development of language, cognitive, and behavioral functions [550]. Mechanistically, myo‐inositol, a component of human milk, enhances the ability of neurons to respond to transsynaptic interactions that induce synapses, promoting neuronal connectivity [656]. Notably, 20‐*α*Hydroxycholesterol in human milk induces oligodendrogenesis in mice through a Gli‐dependent pathway, thereby reversing white matter damage [657]. However, the heterogeneous composition of breast milk due to geographic differences in mothers, dietary habits, health conditions, gestational age at delivery, and other factors, as well as the lack of clarity about which components of breast milk are affected by intestinal microbes, poses a serious challenge for research. Most of the current progress in this area is still limited to emphasizing the metabolic, immunomodulatory, and nutritional support effects of dietary components of maternal gut microbial metabolism on infants via breast milk [658, 659, 660]. Significantly, the impact of breastfeeding on neurodevelopment during crucial developmental periods in infants has yet to be adequately explored. To fully understand the role and potential detrimental impacts of changes in gut microbial metabolites present in breast milk on infants' developmental pathways across various life stages and health statuses, more comprehensive histological studies are needed.

### Maternal gut microbiota influences offspring's neurodevelopment through the microbiota–immunity–gut–brain axis

The early postnatal periods are critical for the immune system development. Beyond genetics and host biology, gut microbes play a substantial and irreversible role in the immune maturation and overall health of infants [590, 661]. Interactions between the gut microbiome and the local immune system lead to functional changes even beyond the gastrointestinal tract, affecting systemic or central immune status and altering neurodevelopmental trajectories. Gut microbial components or soluble mediators released into the environment elicit host adaptive immune responses, stimulate or regulate immune cell differentiation and development, modulate the inflammatory response, and promote barrier integrity [662, 663]. These substances may be involved in the inflammatory and neurodevelopmental axis, thereby affecting or altering neurodevelopmental trajectories. Additionally, there is no shortage of reports related to their direct regulation of the maturation and function of neural cells in the brain, such as microglia, the primary brain‐resident immune cells [664]. In contrast, newborns, with their immature immune system, heavily rely on mother‐derived microorganisms, metabolites, and immune transfers to establish organismal homeostasis in the face of a new external environment (Figure 14B). Maternal immunity and microbial metabolites influenced by gut microbes at the mother–fetus interface, microbiota transfer during pregnancy and lactation, and transfer of immune substances, microbial components (LPS, peptidoglycans, and DNA [665]), and metabolites through breastfeeding are important sources of microorganisms and immune training in the early stages of the offspring's life [459]. The maternal‐derived microbiota acquired by the offspring during delivery can stimulate the activation of intestinal epithelial cells to acquire immune tolerance and regulate natural defenses and innate immune recognition [666]. Microorganisms in breast milk contribute to infant gut microbial colonization and stimulate the activation and differentiation of T cells and IgA‐producing B cells in the neonatal immune system [667]. Meanwhile, the involvement of certain microbial‐derived metabolites in orchestrating the development of the offspring's immune system has been increasingly documented. In mouse models, maternal dietary soluble fiber intake increases the effect of maternal gut microbial metabolites on the offspring's systemic immune responses [668]. The abundance of human milk oligosaccharides and lactoproteins in breast milk are more involved in the regulation and maturation of the offspring's intestinal barrier as the substrate in the early life period, which promotes the intestinal cell differentiation and mucus production and shapes the contour of the immune microenvironment in the intestinal lumen [669, 670]. Also, milk‐derived extracellular vesicles (mEVs) may regulate the intestinal microbiota structure and intestinal immunity of the offspring through inflammatory signaling pathways and activation of inflammatory vesicles [671, 672]. Nevertheless, a comprehensive understanding of the neurodevelopmental outcomes in offspring affected by these factors remains elusive; hence, there is an urgent need to delve into the communication mechanisms and pathways that underlie the microbiota–immunity–brain axis during the early developmental stages. Concerning the transfer of immunological substances, numerous studies have elucidated the critical role of maternal antibody transmission in promoting the healthy development of offspring [673, 674, 675]. For instance, IgA in mouse milk influences the binding of IgA to commensal bacteria in the offspring's intestine, which in turn determines the number of RORγ+ Tregs through a reciprocal inhibitory relationship, influencing intestinal Treg differentiation and functions as well as immune regulatory tone in mouse generations [578, 676]. However, studies on the effect of maternal microbes on the type and efficiency of antibody transfer across the placental cell barrier and through the mammary gland are still limited. Setting aside the complex role of antibody transfer in the immune homeostatic‐neurodevelopmental exchange in early life, it remains unknown whether microbial‐mediated aberrant alterations or excessive amounts of maternally derived antibodies damage brain structure and functions when the blood–brain barrier is incompletely developed in the offspring. This suggests that while focusing on the maternal and infant immunization link, we may also need to evaluate the association between gut microbes and autoimmune disease with additional care [677], particularly by examining antibodies that are known to reach or present in the offspring's brain. Similarly, in addition to antibody transfer, maternal gut microbiota also modulates some of the immune cells, cytokines, and complement components that influence infant development through breast milk. Single‐cell sequencing and flow cytometry assays have shown that immune cells (e.g., B cells, plasma cells, and macrophages) influenced by maternal gut microbiota are also present in human and mouse milk [678]. Complement in breast milk cleaves specific members of the Gram‐positive intestinal commensal bacteria directly through a C1‐dependent, antibody‐independent mechanism, leading to the deposition of membrane attack complexes and subsequent bacterial lysis to protect the gut from pathogens [679]. However, the role of these immune cells and associated immune mediators in the neurodevelopmental processes of offspring during early life remains to be further elucidated.

### Section summary

Early life serves as a critical window for the development of the central nervous and immune systems, as well as the establishment of the gut microbiota, which is sensitive and vulnerable to neurodevelopment. Frequent interactions between a healthy maternal microbiota and the offspring's immune, metabolic, and nervous systems are crucial for guiding brain development in the offspring. Herein, we summarized the possible pathways by which maternal gut microbiota regulates offspring's neurodevelopment from the perspectives of microbial colonization, gene expression, immunity, and metabolism. Despite extensive research on the interactions between the gut microbiota and the nervous system and their transgenerational effects, the communication within the microbiota–gut–brain axis remains intricate and currently challenging to fully comprehend. The decoding of the maternal–fetal interface and the application of various sequencing methods have gradually clarified the pathways by which the maternal microbiota communicates with the fetus. Through a comprehensive analysis of extensive publicly available data sets pertaining to microbiome, we can achieve an in‐depth understanding of the colonization and transformation dynamics of the infant gut microbiome. Moreover, to further enhance our understanding of this intricate biological event occurring in early life, it is imperative to differentiate the structure and functions of the microbiome. This requires future research to delve deeper into elucidating the specific mechanisms underlying maternal gut microbiome transmission and monobacterial colonization in offspring. Additionally, emphasis should be placed on investigating how these processes impact brain health and disease development in the offspring, thereby contributing to a more profound comprehension of basic metabolic, immune, and physiological processes facilitated by this integrative approach. Moreover, there remains a pressing need for more targeted research to develop a thorough understanding of the interactions among various microorganisms, including maternal gut fungal and viral communities, and their influence on the homeostatic functioning of the host system and pregnancy health. Such research should aim to elucidate the potential roles of these microbial networks in offspring neurodevelopment. Investigating variations in gene structure and adaptive evolution during bacterial transmission will facilitate the identification of novel targets derived from the intrinsic capabilities of bacterial communities, which can be leveraged to enhance health and prevent diseases [680, 681, 682]. This endeavor will require extensive longitudinal studies, comprehensive genomic analyses, and rigorous clinical intervention trials.

During pregnancy, the maternal gut microbiota and its associated microbial metabolites are highly dynamic and subject to multiple factors, and these signals serve as the basis for the balance of metabolic profiles in the offspring and the calibration of cellular transcriptional programs. Similar to the metabolic and physiological changes that occur throughout pregnancy, the maternal immune system adapts to the different stages of developmental changes in pregnancy to promote and indoctrinate the offspring's immune development. The neuro‐immune system interactions play an important role in contributing to the maintenance and development of normal neurological functions [462, 672]. Extensive studies using poly I:C and LPS to mimic viral and bacterial infections, respectively, have highlighted common mechanisms affecting offspring neurodevelopment at the maternal–fetal interface. However, the specific impact of particular bacteria, especially those prevalent regionally or in hospitals, on neurodevelopment and immune responses remains underexplored.

A large body of literature suggests that breastmilk has a greater impact on offspring's health and neurodevelopment than formula, controlling for environmental and social factors. This is particularly true for bioactive components [683], such as nutrients, energy metabolism‐related substances, hormones, and neurotransmitters, which are crucial for offspring's development and can be synthesized or metabolized by microorganisms present during the early stages of life. Recent studies suggest that microbiota‐derived metabolites possess the potential to modulate epigenetic inheritance [684, 685]. Consequently, it is proposed that these metabolites are likely to play a crucial role in the interplay between immune‐metabolic functions and neuronal cell fate in offspring. Addressing these research gaps may lead to the goal of improving offspring development and lifelong health during breastfeeding. It also suggests that there is value in determining the origin of substances in breast milk and the utilization of substances in the early life of the offspring. Methodologically, more targeted and untargeted spatial metabolomics, as well as spatial proteomics studies, are needed to discuss and correlate microbiome‐associated metabolites and metabolic pathways with neuroprotective or neurorestorative potential that play a planning role in offspring development. It should be clear that maternal oral and mucosal health, barrier health, autoimmune disease, and gut homeostasis during pregnancy and lactation may all influence the neurodevelopment of the offspring [686, 687, 688, 689].

Moreover, amidst the diversity of maternal gut microbiota and the intricacy of transgenerational impacts, contemporary methods—encompassing high‐throughput sequencing technologies, germ‐free mouse models, and culturomics—enable the gradual identification of pivotal maternal microbial species crucial for offspring's neurodevelopment. The progression in labeling and tracking imaging technologies has granted a more profound understanding of the growth, migration, and metabolic processes of microbes and their byproducts within living organisms. Leveraging integrated big data analytics and machine learning harbors substantial promise for enhancing our comprehension of these intricate biological interplays. Through the application of spatiotemporal mapping and multiomics integrations, such as genomics, metabolomics, and proteomics, researchers can elucidate the destinies of pertinent tissues and cells in impacted offspring and hunt for factors that elevate vulnerability to nerve damage. Merging these methodologies with neuroimaging techniques, like functional magnetic resonance imaging (fMRI), may furnish a novel vantage point on the communication and interaction between gut and brain cells, thereby affording deeper clinical insights into the quest for identifying widespread targets for neurodevelopmental disorders and the prophylaxis of brain afflictions.

Acknowledging the neonatal brain's susceptibility to injuries, it is equally important to highlight its extraordinary capacity for regeneration. Timely interventions and treatments are essential to mitigate neurodevelopmental risks. Furthermore, the emerging field of gut–brain axis research underscores the significant potential of the gut as a therapeutic target, especially in relation to neurodevelopment. FMT presents a promising avenue for the treatment of neurodevelopment disorder, such as autism spectrum disorder (ASD), grounded in the hypothesis that disruptions in the gut microbiota may play a pivotal role in the etiology of ASD. Preliminary investigations have reported favorable outcomes, suggesting that FMT may modulate gut microbiota and potentially ameliorate ASD‐related symptoms [690]. Nonetheless, comprehensive research is imperative to elucidate the underlying mechanisms, establish the efficacy, and assess the long‐term consequences of FMT in the context of ASD therapy. Probiotics and prebiotics, as safe and effective modulators of gut microbiota [691, 692], offer promising avenues for addressing chronic inflammatory conditions and enhancing overall health, thereby highlighting their potential as invaluable additions to the therapeutic repertoire specifically for promoting neurodevelopment.

## CONCLUSION

**Figure 15:**
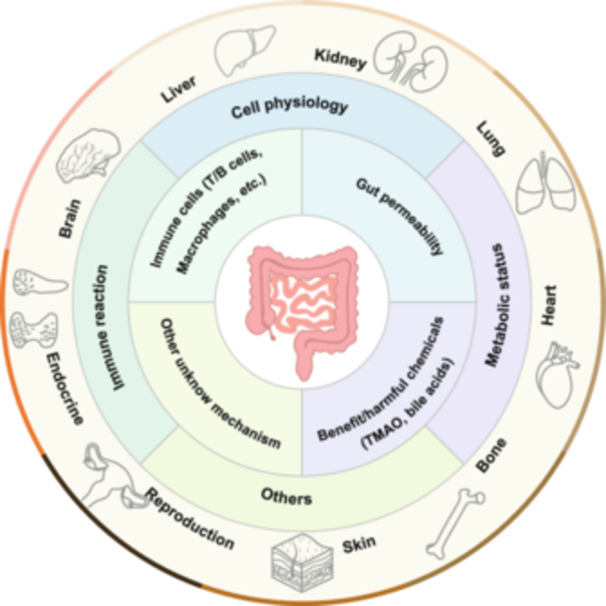
The general modulatory mechanism of “Gut‐X axis”. The intestine, intestinal microbiota and microbial‐derived chemicals could influence immune cell functions, gut permeability and other unknown mechanisms to regulate multiple organs' pathophysiology, including metabolic status, immune reactions, and so forth.

Intestinal microbiota could influence extraintestinal organs through multiple pathways, including immunomodulation, host cell death, metabolism, and so forth (Figure 15). In contrast, extraintestinal organs may also impact gut microbiota at both compositional and functional levels. Thus, all of the axes may be bidirectional. In addition, the promising strategy to precisely manipulate the gut microbiota may help us to combat the diseases of extraintestinal organs in the future.

## AUTHOR CONTRIBUTIONS

**Xu Lin:** Conceptualization; writing—original draft; writing—review and editing. **Zuxiang Yu:** Writing—original draft; writing—review and editing. **Yang Liu:** Writing—original draft; writing—review and editing. **Changzhou Li:** Writing—original draft; writing—review and editing. **Hui Hu:** Writing—original draft; writing—review and editing. **Jia‐Chun Hu:** Writing—original draft; writing—review and editing. **Mian Liu:** Writing—original draft; writing—review and editing. **Qin Yang:** Writing—original draft; writing—review and editing. **Peng Gu:** Writing—original draft; writing—review and editing. **Jiaxin Li:** Writing—original draft; writing—review and editing. **Kutty Selva Nandakumar:** Writing—original draft; writing—review and editing. **Gaofei Hu:** Writing—original draft; writing—review and editing. **Qi Zhang:** Writing—original draft; writing—review and editing. **Xinyu Chen:** Writing—original draft; writing—review and editing. **Huihui Ma:** Writing—original draft; writing—review and editing. **Wenye Huang:** Writing—original draft; writing—review and editing. **Gaofeng Wang:** Conceptualization; writing—original draft; writing—review and editing; funding acquisition. **Yan Wang:** Conceptualization; funding acquisition; writing—original draft; writing—review and editing. **Liping Huang:** Conceptualization; funding acquisition; writing—original draft; writing—review and editing. **Wenjuan Wu:** Conceptualization; funding acquisition; writing—original draft; writing—review and editing. **Ning‐Ning Liu:** Conceptualization; funding acquisition; writing—original draft; writing—review and editing. **Chenhong Zhang:** Conceptualization; funding acquisition; writing—original draft; writing—review and editing. **Xingyin Liu:** Conceptualization; funding acquisition; writing—review and editing; writing—original draft. **Leming Zheng:** Conceptualization; funding acquisition; writing—original draft; writing—review and editing. **Peng Chen:** Conceptualization; funding acquisition; writing—original draft; writing—review and editing.

## CONFLICT OF INTEREST STATEMENT

The authors declare no conflict of interest.

## ETHICS STATEMENT

No new animal or human experiments were involved in this study. All data were obtained from publicly available research and complied with ethical standards.

## Data Availability

Data sharing is not applicable to this article as no new data were created or analyzed in this study. Supplementary materials (graphical abstract, slides, videos, Chinese translated version and update materials) may be found in the online DOI or iMeta Science http://www.imeta.science/.
