# Correction to “Gut–X axis”

**DOI:** 10.1002/imt2.70057

**Published:** 2025-06-22

Lin, Xu, Zuxiang Yu, Yang Liu, Changzhou Li, Hui Hu, Jia‐Chun Hu, Mian Liu, et al. 2025. “Gut–X axis.” *iMeta* 4: e270. https://onlinelibrary.wiley.com/doi/abs/10.1002/imt2.270

1. In Figure 7 of the “Gut–Heart Axis” section, the word “myocarditis” was incorrect; this should be “myocardial injury.” The paragraph “myocardial injury” includes diseases or pathological injuries that fall under the category of myocardial injury and cannot be equated with the category of cardiomyopathy. This is a misuse of the noun here. Furthermore, in Figure 7 of the “Gut–Heart Axis” section, the word “PAGly” should be deleted. Because the paragraph “myocardial injury” didn't describe “PAGly.” The revised Figure 7 and figure legend are as follows.

**Figure 7:**
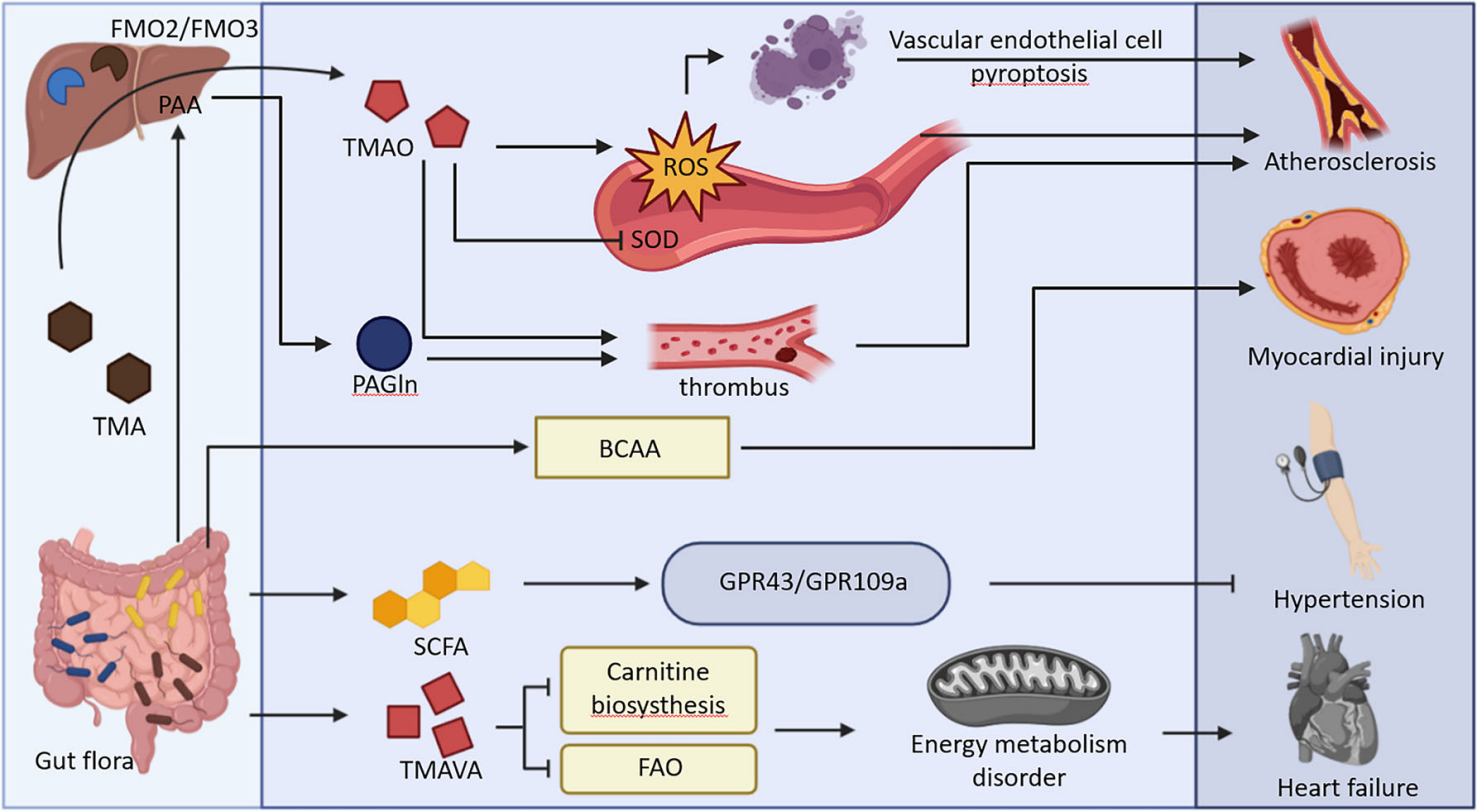
Gut microbiome metabolites (TMAO, PAGln, SCFA, and TMAVA) influence CVD development through various mechanisms. TMA produced by the gut microbiome is converted in the liver to produce TMAO, which aggravates AS by promoting vascular endothelial cell pyroptosis through the induction of oxidative stress and the production of NLRP3. PAGln is another gut metabolite that accelerates thrombosis and thus exacerbates AS. BCAA is a bacterial metabolite that is strongly associated with myocardial injury. In addition, SCFA produced by the gut microbiome was found to influence the progression of hypertension via GPR43/GPR109a. Finally, the bacterial metabolite TMAVA, which can lead to impaired energy metabolism and thus affect heart failure. Created with BioRender.com.

2. In the “Gut–Heart Axis” section, the word “PAGIn” was incorrect, it should be “PAGln.”

3. The corresponding author's name “Leming, Zheng” is misspelled, it should be “Lemin, Zheng.”

We apologize for these errors.

